# *Troy/Tnfrsf19* marks epidermal cells that govern interfollicular epidermal renewal and cornification

**DOI:** 10.1016/j.stemcr.2021.07.007

**Published:** 2021-08-05

**Authors:** Kai Kretzschmar, Kim E. Boonekamp, Margit Bleijs, Priyanca Asra, Mandy Koomen, Susana M. Chuva de Sousa Lopes, Barbara Giovannone, Hans Clevers

**Affiliations:** 1 Oncode Institute, Hubrecht Institute, Royal Netherlands Academy of Arts and Sciences (KNAW) and University Medical Centre (UMC) Utrecht, 3584 CT Utrecht, the Netherlands; 2 Mildred Scheel Early Career Centre (MSNZ) for Cancer Research Würzburg, University Hospital Würzburg, 97080 Würzburg, Germany; 3 German Cancer Research Centre (DKFZ), 69120 Heidelberg, Germany; 4 Princess Máxima Center for Pediatric Oncology, 3584 CS Utrecht, the Netherlands; 5 Leiden UMC, 2333 ZC Leiden, the Netherlands; 6 UMC Utrecht, 3584 CX Utrecht, the Netherlands

**Keywords:** *Troy*, *Tnfrsf19*, stem cells, epidermis, organoids, lineage tracing, single-cell transcriptomics

## Abstract

The skin epidermis is a highly compartmentalized tissue consisting of a cornifying epithelium called the interfollicular epidermis (IFE) and associated hair follicles (HFs). Several stem cell populations have been described that mark specific compartments in the skin but none of them is specific to the IFE. Here, we identify *Troy* as a marker of IFE and HF infundibulum basal layer cells in developing and adult human and mouse epidermis. Genetic lineage-tracing experiments demonstrate that *Troy*-expressing basal cells contribute to long-term renewal of all layers of the cornifying epithelium. Single-cell transcriptomics and organoid assays of Troy-expressing cells, as well as their progeny, confirmed stem cell identity as well as the ability to generate differentiating daughter cells. In conclusion, we define *Troy* as a marker of epidermal basal cells that govern interfollicular epidermal renewal and cornification.

## Highlights

• *TROY/Troy* marks basal layer cells of developing and adult human and mouse epidermis
• *Troy*marks stem cells that produce early differentiated cells in the basal layer
• *Troy*-expressing basal cells give rise to all layers of the cornified epidermis
• *Troy* marks cells that contribute to epidermal homeostasis in the long term

## Graphical abstract

**Figure:**
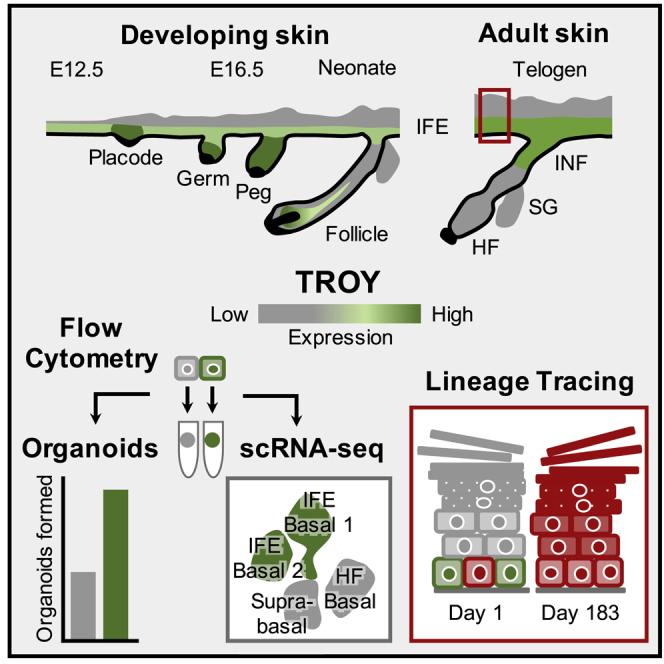

## Teaser

In this article, Clevers, Kretzschmar, and colleagues show that the Wnt target gene *Troy/Tnfrsf19* marks basal cells in the interfollicular epidermis of human and murine skin. Using whole-mount imaging, single-cell transcriptomics, lineage tracing, and organoid technology, the authors demonstrate in mice that *Troy*-expressing cells have stem cell capacity *in vitro* and *in vivo* for regenerating the cornifying epidermis in the long term.

## Introduction

Mammalian skin acts as a protective mechanical and biological barrier against injuries, foreign pathogens, and loss of heat and water. Critical to the skin’s main function is its outermost layer, the epidermis, which is comprised of a multi-layered epithelium, the interfollicular epidermis (IFE), and associated hair follicles (HFs), sebaceous glands (SGs), and sweat glands (in mice only in the paws). Directly exposed to the body’s outside is the cornified envelope of the epidermis, also known as the stratum corneum. This epidermal layer consists of enucleated, organelle-free cells, which are enriched in highly crosslinked filamentous keratins and other cytoskeletal proteins in their cytoplasm. The cornified envelope is mainly generated by the IFE. The stratified squamous epithelium of the IFE is heavily studied using cultured human and murine keratinocytes or mouse models to explore adult homeostasis and perturbations, such as wounding or diseases.

Pioneering work by Rheinwald and Green used single human epidermal cells, so-called keratinocytes, cultured on a layer of inactivated mouse fibroblasts acting as feeder cells. This approach established in the mid-1970s that human epidermis contains cells with the capacity to generate a stratified squamous epithelium *in vitro* and, hence, show stemness potential (Rheinwald and Green, 1975). Subsequently, starting from the—now contested—notion that adult stem cells are DNA-label-retaining cells, epidermal stem cells were thought to be located to a specialized niche in the HFs in mouse and human epidermis, called the bulge (Cotsarelis et al., 1990). Genetic lineage-tracing experiments performed in mice have since provided evidence for the presence of numerous stem cell populations in the adult epidermis contributing to tissue homeostasis and regeneration (Figures S1A and S1B). Stem cells in the lower HF (bulge and hair germ) are marked by *Axin2*, *Cd34*, *Gli1*, *Krt15*, *Krt19*, *Lgr5*, and *Sox9* (Brownell et al., 2011; Jaks et al., 2008; Kadaja et al., 2014; Kretzschmar et al., 2016; Lim et al., 2016; Morris et al., 2003; Nowak et al., 2008)*.* Stem cells in the upper HF (isthmus, junctional zone, and infundibulum) are marked by *Gli1*, *Lgr6*, *Lrig1*, and *Plet1*/MTS24 (Brownell et al., 2011; Füllgrabe et al., 2015; Jensen et al., 2009; Kretzschmar et al., 2016; Nijhof et al., 2006; Page et al., 2013; Raymond et al., 2010; Snippert et al., 2010). Stem cells in the SGs are marked by *Lgr6* and *Lrig1* and stem cells in the IFE are marked by *Axin2* and *Lgr6* (Füllgrabe et al., 2015; Kretzschmar et al., 2016; Lim et al., 2013; Page et al., 2013). Collectively, this suggests that marker genes are typically not specific to one epidermal compartment. Throughout the epidermis, stem cells are located to the bottom-most layer of the epithelium, the basal layer also known as stratum basale, which is marked by the basal keratins KRT5 and KRT14 and is in direct contact to the basement membrane (Fuchs and Weber, 1994). Extracellular matrix proteins, such as laminins and collagens, are highly enriched in the basement membrane and bind integrins, such as α6 integrin (ITGA6) and β1 integrin (ITGB1) that are highly expressed by epidermal basal layer cells in humans and mice (Jones et al., 1995; Jones and Watt, 1993; Kretzschmar and Watt, 2014). While many markers of human epidermal stem and progenitor cells have been proposed, a common marker shared by such cells contributing to IFE differentiation in both humans and mice is lacking.

Canonical Wnt signaling plays a critical role in epidermal development, homeostasis, and regeneration (Kretzschmar and Clevers, 2017). Interestingly, a great number of murine epidermal stem cell markers are bona fide Wnt/β-catenin target genes and also found to be specific to adult stem cells in other epithelia. *Lgr5*, for example, has initially been defined as a marker gene of intestinal epithelial stem cells and was later found to be expressed by adult stem cells in the lower HF and various other epithelial tissues throughout the body (Barker et al., 2007, 2010; Huch et al., 2013; Jaks et al., 2008). Based on this observation, we aimed to explore the potential role of TROY in embryonic and adult mouse epidermis. TROY is expressed by a Wnt/β-catenin target gene also known as tumor necrosis factor receptor superfamily, member 19 (*TNFRSF19* or *TROY* in humans and *Tnfrsf19* or *Troy* in mice) and has already been defined to mark adult stem cells in the intestinal and gastric epithelium as well as in adult neuronal stem cells (Basak et al., 2018; Fafilek et al., 2013; Stange et al., 2013).

Expression of TROY in skin has been described during skin (embryonal and neonatal) development, suggesting a potential role there (Kojima et al., 2000). In addition, *Troy* was found to be enriched in the basal layer of the infundibulum (INF) and IFE through single-cell transcriptomics on adult murine skin (Joost et al., 2016). However, no obvious skin phenotype has been demonstrated for TROY-deficient (*Troy*^+/–^) mice (Pispa et al., 2008). TROY shares homology with other TNF receptor members called EDAR and XEDAR, suggesting possible functional redundancy (Hashimoto et al., 2008; Kojima et al., 2000). *Troy*^–/–^*Eda*^–/–^ mice lacking expression of TROY as well as EDA, the ligand of EDAR and XEDAR, show strong defects in HF development (Pispa et al., 2008) revealing an important role for TROY/EDA signaling during skin development. Knowledge on the role of TROY^+^ cells in adult skin is missing. Here, we therefore map TROY expression in embryonic, neonatal, and adult skin, and assess the contribution of TROY^+^ cells to epidermal adult homeostasis.

## Results

### *TROY* marks interfollicular and infundibular epidermal cells in telogen skin

**Figure 1:**
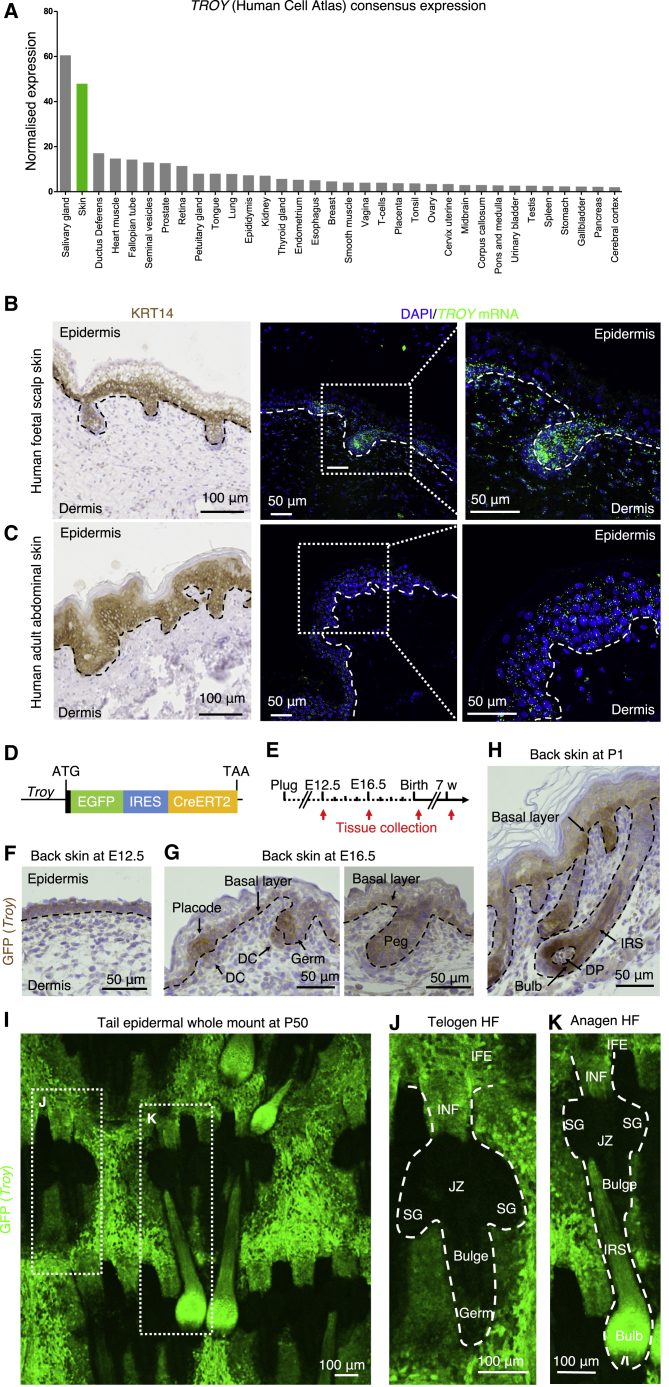
*TROY* expression in human and mouse embryonic, fetal, and adult skin (A) Normalized Human Protein Atlas consensus *TROY* mRNA expression. (B and C) Paraffin sections of human fetal scalp skin (B) and human adult abdominal skin (C) stained for K14 and RNAscope staining of human *TROY* transcripts. (D) Schematic representation of the *Troy*-EGFP knockin mouse model. (E) Experimental timeline of tissue collection. (F–H) Paraffin sections of E12.5 (F), E16.5 (G), and P1 (H) back skin stained for EGFP. DC, dermal condensate; DP, dermal papilla; IRS, inner root sheath. (I–K) Tail epidermal whole mounts (P50) of *Troy*-EGFP mice stained for EGFP. Outlined are a telogen hair follicle (J) and anagen hair follicle (K). IFE, interfollicular epidermis; INF, infundibulum; JZ, junctional zone; SG, sebaceous gland. See also Figures S1 and S2.

Based on consensus data generated by the Human Protein Atlas program (Uhlén et al., 2015), we found that the skin was the human tissue with the second highest normalized expression of *TROY* (Figure 1A). We next performed RNAscope analyis on paraffin sections of both fetal and adult human skin, which allows for the visualization of mRNA transcripts in intact cells (Wang et al., 2012). We found *TROY* mRNA transcripts in the epidermis of both human fetal scalp and adult abdominal skin (Figures 1B and 1C). In human fetal scalp skin, *TROY* was mostly confined to the KRT14^+^ epidermal basal layer and developing HFs with a notable enrichment of transcripts in the HFs (Figure 1B). In human adult abdominal skin, *TROY* mRNA transcripts were more widely detected across the stratified epithelium, including its KRT14^+^ cell layers (Figure 1C).

To determine the localization of TROY expression in murine skin, we used the *Troy*^EGFP-IRES-CreERT2^ mouse model (Stange et al., 2013). In this model, an expression cassette harboring enhanced green fluorescent protein (EGFP) and Cre recombinase fused to a tamoxifen inducible, mutated version of the human estrogen receptor (CreERT2) is knocked into the *Troy* locus replacing its protein-coding region (Figure 1D). We collected tissue from different skin regions, including back and tail at different time points of development and homeostasis and stained for EGFP as a proxy for TROY expression (Figure 1E). At embryonic day 12.5 (E12.5), when the epidermis is an undifferentiated layer between the periderm and dermis, EGFP expression in back skin was confined to epidermal cells (Figure 1F). As development progresses, at E16.5, we found EGFP immunoreactivity specific to the IFE basal layer and developing HFs (Figure 1G). EGFP expression was most prominent in the areas of the hair placode as well as in the hair shaft-forming regions of the hair germ and peg (Figure 1G), while EGFP positivity was also detectable in the dermal condensate (Figure 1G). In neonatal skin, at post-natal day 1 (P1), when the HF forms as a bulbous peg, EGFP expression remained present in the IFE basal layer as well as in the hair bulb and hair shaft-forming inner root sheath (IRS) (Figure 1H). However, EGFP expression was absent from the outer root sheath as well as the upper HF portion generating the isthmus, junctional zone and SG (Figure 1H). Next, we stained epidermal whole mounts (Braun et al., 2003) collected from the tail skin of *Troy*^EGFP-IRES-CreERT2^ mice at P50 and stained for EGFP (Figures 1I–1K). EGFP expression was confined to the IFE and INF (Figures 1J and 1K) as well as the hair bulb and IRS of anagen HFs (Figures 1I and 1K). Sections of various adult murine skin tissues stained for EGFP showed robust immunoreactivity in the basal layer of IFE and INF of back, ear, and tail, as well as the epidermis of paw skin, which is devoid of HFs (Figure S2). These results confirmed *Troy* expression throughout HF development and showed confined *Troy* expression in the IFE (and INF associated with telogen HFs) in adult skin.

### *Troy*-expressing basal layer cells are SCA1^+^ and highly proliferative

**Figure 2:**
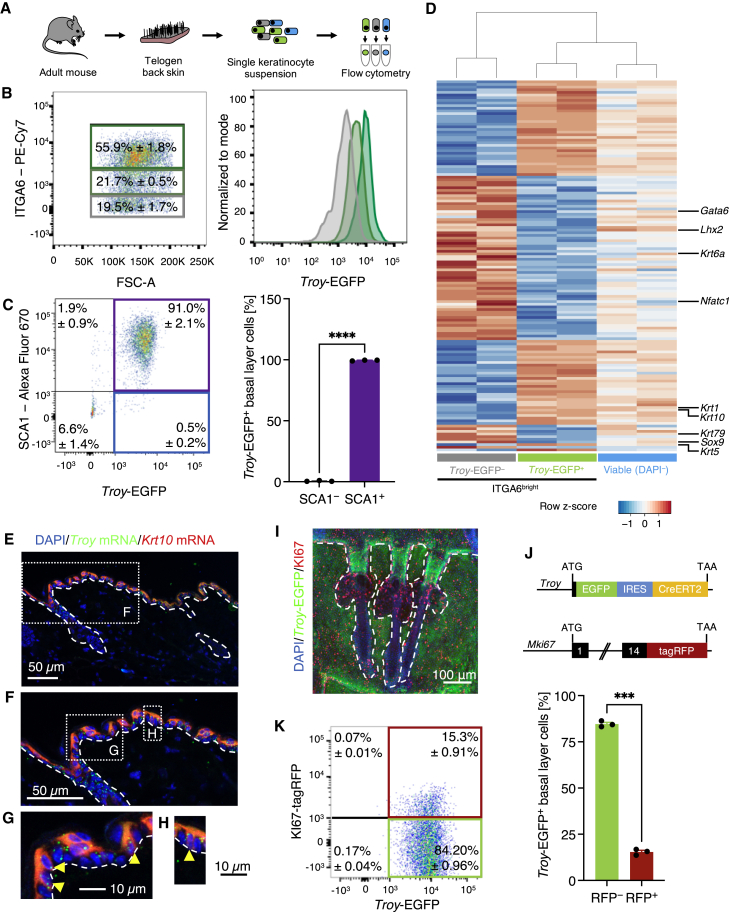
Characterization of *Troy*-expressing cells in adult murine telogen skin (A) Experimental setup. (B and C) Representative flow cytometry scatterplots of viable cells isolated from *Troy*-EGFP knockin mice stained for ITGA6 (CD49f) (B) and SCA1 (C). Histogram of *Troy*-EGFP expression normalized to mode (B). Dark green, ITGA6^bright^ cells; green, ITGA6^mid^ cells; gray, ITGA6^dim^ cells (B). Column chart indicating the percentage of SCA1^–^ and SCA1^+^ cells within the *Troy*-EGFP^+^ population (C). The data are presented as mean ± SEM (n = 3 mice). Student's t test, ^∗∗∗∗^p < 0.0001. (D) Heatmap of the bulk mRNA sequencing showing the differentially expressed genes comparing *Troy*-EGFP^+^ and *Troy*-EGFP^–^ ITGA6^bright^ cells. (E–H) Representative image of paraffin sections of telogen back skin stained RNAscope probes against *Troy/Tnfrsf19* and *Krt10*. Yellow arrows in (G) and (H) indicate basal layer cells positive for *Krt10* mRNA and *Troy* mRNA. (I) Tail epidermal whole mount of *Troy*-EGFP mice stained for EGFP and KI67. (J) Schematic representation of the genetic constructs. (K) Representative flow cytometry scatterplot of viable SCA1^+^ ITGA6^+^ cells assessed for expression of *Troy*-EGFP and KI67-tagRFP. Column chart indicating the percentage of KI67-tagRFP^–^ and KI67-tagRFP^+^ cells within the *Troy*-EGFP^+^ population. The data are presented as mean ± SEM (n = 3 mice). Student's t test, ^∗∗∗^p < 0.001. See also Figure S3 and Table S1.

To better characterize the properties of *Troy*-expressing cells, we collected the telogen skin of adult *Troy*^EGFP-IRES-CreERT2^ mice, isolated epidermal cells, and performed flow cytometry (Figure 2A). We separated the epidermal cells into three fractions based on their expression of ITGA6 (CD49f) and assessed EGFP expression (Figures 2B and S3A). Basal layer cells, marked by high levels of ITGA6, showed the highest expression intensity of EGFP (Figure 2B). With decreasing expression levels of ITGA6, the levels of EGFP expression decreased too, suggesting that TROY is highly associated with epidermal basal layer cells. In line with our observations above, we found that virtually all *Troy*-EGFP^+^ basal epidermal cells (>99%), were SCA1^+^, confirming their IFE/INF identity (Figure 2C). This was further supported by bulk messenger RNA (mRNA) sequencing performed on ITGA6^bright^ basal epidermal cells sorted based on EGFP expression (Figures 2D and S3B–S3E). We found 140 differentially expressed genes (adjusted p < 0.05) when comparing *Troy*-EGFP^+^ and *Troy*-EGFP^–^ ITGA6^bright^ basal layer cells (Figures 2D and S3B–S2E; Table S1). *Troy*-EGFP^+^ basal epidermal cells were enriched for genes associated with the IFE lineage, such as *Krt1* and *Krt10* (Figures 2D and S3D)*.* Among the significantly downregulated genes in the *Troy*-EGFP^+^ cell population were the HF bulge markers *Krt6a*, *Lhx2*, *Nfatc1*, and *Sox9*, as well as the hair canal marker *Krt79* and the sebaceous duct marker *Gata6* (Figures 2D and S3D). Expression of the differentiation marker KRT10 in the epidermal basal layer has previously been observed (Braun et al., 2003). To validate our *in silico* data, we performed RNAscope for *Troy* and *Krt10* on sections of back skin of wild-type mice (Figures 2E–2H). Indeed, several epidermal basal cells showed co-expression of both *Troy* and *Krt10* confirming our RNA sequencing data (Figures 2G and 2H). Since actively cycling cells can be found throughout the epidermal basal layer (Figure S3F), we next investigated the proliferative status of *Troy*-EGFP^+^ cells. We first stained tail epidermal whole mounts of adult *Troy*^EGFP-IRES-CreERT2^ mice for EGFP and KI67, a marker of actively cycling cells (Hutchins et al., 2010). We found co-expression of EGFP and KI67 in basal layer cells of IFE and INF (Figure 2I). To quantify the overlap of EGFP and KI67, we generated mice harboring both *Troy*^EGFP-IRES-CreERT2^ and *Mki67*^tagRFP^ expression cassettes (Basak et al., 2018) (Figure 2J) and isolated epidermal cells from adult telogen back skin. Using flow cytometry, we determined that virtually all KI67-tagRFP^+^ IFE/INF basal (SCA1^+^ ITGA6^bright^) epidermal cells were *Troy*-EGFP^+^ (>99%; Figure S3G) and about 15% of all *Troy*-EGFP^+^ basal epidermal cells were positive for KI67-tagRFP (Figure 2K). This is in line with previous studies showing that proliferation in the IFE basal layer ranges from about 9% (via DNA content measurement) and 17.7% (via bromodeoxyuridine incorporation) (Cianfarani et al., 2011; Mascré et al., 2012). Taken together, these data indicate that *Troy* marks IFE/INF basal cells and enriches for proliferative cells.

### *Troy*-expressing basal layer cells are highly clonogenic in organoid cultures

**Figure 3:**
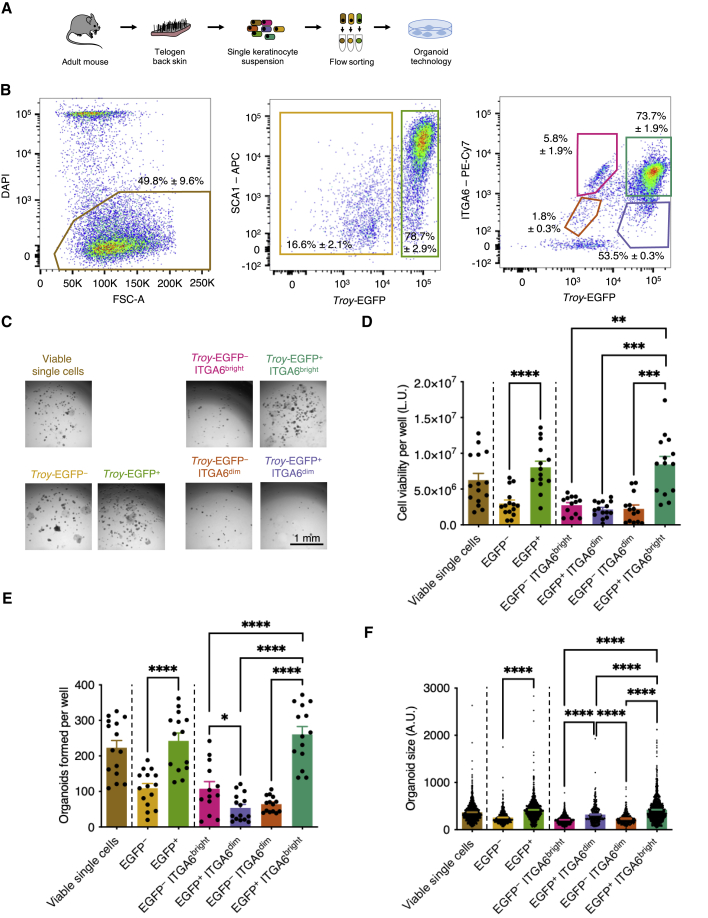
Characterization of *Troy*-expressing cells using organoid technology (A) Experimental setup. (B) Representative flow cytometry scatterplots of viable cells isolated from *Troy*-EGFP knockin mice stained for ITGA6 and SCA1 (n = 4). (C) Representative bright-field images of sorted cell populations grown as organoids for 7 days. (D–F) Quantification of the cell viability per well (D), number of organoids formed per well (E), and size of organoids formed (F) after 7 days of culture. The data are presented as mean ± SEM (two to four replicates/wells per mouse, n = 4 mice). Student's t test, ^∗∗^p < 0.01, ^∗∗∗^p < 0.001, ^∗∗∗∗^p < 0.0001. A.U., arbitrary units; L.U., light units.

To assess the clonogenic potential of *Troy*-EGFP^+^ cells, we performed organoid-forming efficiency (OFE) assays (Figure 3A). In this functional assay, organoids grown from a single cell serve as a proxy for stem cell capacity (Boonekamp et al., 2019). We purified different cell populations using flow cytometry (Figure 3B), plated single cells, and assessed organoid formation 7 days later (Figure 3C). Organoid cultures generated from *Troy*-EGFP^+^ cells had a significantly higher cell viability and contained more and larger organoids in comparison with cultures derived from *Troy*-EGFP^–^ cells (Figures 3C–3F). In a second step, we analyzed organoid formation from four different cell populations, which were sorted based on their expression of *Troy*-EGFP and the basal layer marker ITGA6 (Figure 3B). Firstly, ITGA6^bright^ basal layer cells positive for *Troy*-EGFP showed a higher OFE than those negative for the reporter (Figures 3C–3F). Secondly, organoid cultures generated from ITGA6^dim^ suprabasal cells had a lower cell viability and contained fewer organoids—irrespective of their level of *Troy*-EGFP expression—than those of *Troy*-EGFP^+^ ITGA6^bright^ cells (Figures 3C–3F). Thirdly, overall cell viability was comparable between organoid cultures of *Troy*-EGFP^–^ ITGA6^bright^ basal cells and those derived from *Troy*-EGFP^+^ ITGA6^dim^ suprabasal cells (Figure 3D). However, *Troy*-EGFP^–^ ITGA6^bright^ basal cells formed more organoids than *Troy*-EGFP^+^ ITGA6^dim^ cells (Figure 3E), while the organoids generated from *Troy*-EGFP^+^ ITGA6^dim^ suprabasal cells were significantly larger than organoid derived from either *Troy*-EGFP^–^ ITGA6^bright^ cells or *Troy*-EGFP^–^ ITGA6^dim^ cells (Figure 3F). Taken together, these data suggest that *Troy*-EGFP^+^ basal layer cells are highly clonogenic. In addition, *Troy*-EGFP^+^ differentiating cells in the suprabasal layer expectedly had a lower capacity to form organoids. However, some of these suprabasal *Troy*-EGFP^+^ cells appeared plastic and responsive to stemness-promoting growth factors, as the capacity to form larger organoids was increased in comparison with both *Troy*-EGFP^–^ basal and suprabasal cells (Figure 3F). This is line with previous observations showing that some suprabasal cells reacquire stem cell capacity under certain conditions (Donati et al., 2017; Kretzschmar et al., 2014).

### Single-cell transcriptomics reveals that *Troy* marks two distinct populations of epidermal basal layer cells

**Figure 4:**
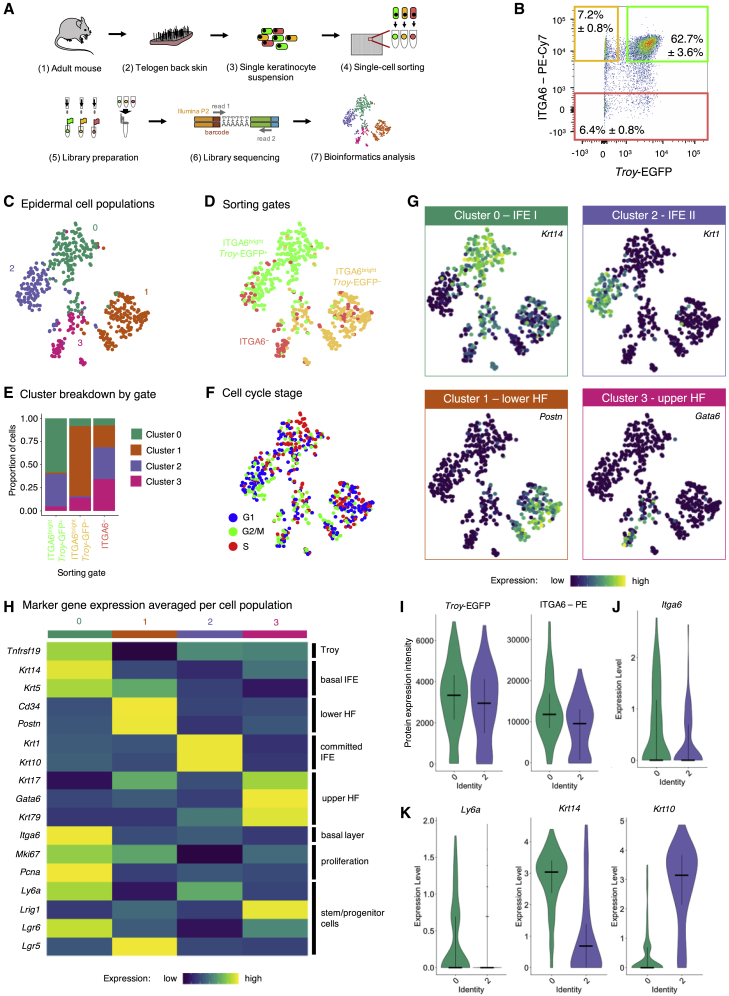
Single-cell transcriptomics of *Troy*-expressing cells and other epidermal cells (A) Experimental setup. (B) Representative flow cytometry scatterplots of viable cells isolated from *Troy*-EGFP knockin mice stained for ITGA6 (n = 4). Sorting gates are indicated in colors: ITGA6^–^ cells (red), ITGA6^bright^*Troy*-EGFP^–^ cells (yellow), and ITGA6^bright^*Troy*-EGFP^+^ cells (green). (C and D) *t*-SNE plot indicating the four different clusters identified within the epidermal cell populations (C) and the sorting gates (D). (E) Stacked column chart indicating cluster breakdown by gate. (F) Cell-cycle analysis. (G) Key marker gene expression per cluster. (H) Heatmap showing marker gene expression averaged per cell population for all identified epidermal clusters. (I–K) Violin plots comparing the EGFP and ITGA6 protein expression intensity (I), *Itga6* expression levels (J), and the expression levels of *Ly6a*, *Krt14*, and *Krt10* (K) between the cells in clusters 0 and 2. Horizontal bars indicate the median and cross bars indicate the quartiles. See also Figure S4.

To characterize the *Troy*-EGFP^+^ cells in more depth and gain insights into possible heterogeneity, we sorted cells into 384-well plates and performed single-cell mRNA sequencing (Figure 4A). We separated the cells based on expression of *Troy*-EGFP and ITGA6 (Figure 4B) and sorted three populations: (1) (ITGA6^–^) non-basal layer cells, (2) *Troy*-EGFP^–^ (ITGA6^bright^) basal layer cells, and (3) *Troy*-EGFP^+^ (ITGA6^bright^) basal layer cells and recorded flow cytometry parameters (Baron et al., 2019). Subsequently, samples were processed for mRNA sequencing using the SORT-seq method (Muraro et al., 2016) and data were analyzed using Seurat (Butler et al., 2018). After quality control and filtering of necrotic cells and non-epidermal cells (Figures S4A–S4E), remaining epidermal cells separated into four different clusters (Figures 4C–4E). Actively cycling cells were found in all clusters, with cluster 0 showing enrichment for cells in the S phase of the cell cycle (Figure 4F). In their comprehensive single-cell transcriptomics dataset of epidermal cells, Joost et al. (2016) defined different main populations of epidermal cells based on their markers. Based on these markers, we identified the four different populations (Figures 4G and 4H): We found that ITGA6^–^ cells contributed mainly to cluster 3, which—based on their high expression of *Gata6—*contained upper HF cells. ITGA6^bright^ *Troy*-EGFP^–^ basal layer cells were enriched in cluster 1 representing lower HF cells marked by *Postn*. ITGA6^bright^ *Troy*-EGFP^+^ basal layer cells contributed to clusters 0 and 2, marked by *Krt14* and *Krt1*, respectively. As ITGA6^bright^*Troy*-EGFP^+^ cells clustered into two different epidermal cell populations, we aimed to gain more insights into these two cell clusters. We therefore projected the flow cytometry data recorded during cell sorting (index sorting) onto the transcriptomic data of each cell. Clusters 0 and 2 showed similar intensities for EGFP and ITGA6 (Figure 4I). *Itga6* and *Ly6a* (encoding the IFE basal cell marker SCA1) gene expression was not altered between both clusters (Figures 4J and K). However, gene expression of *Krt14* were strongly reduced in cluster 2, while *Krt10* expression was robustly upregulated (Figure 4K). Interestingly, our data therefore imply the presence of two distinct IFE basal cell populations marked by ITGA6^bright^ and *Troy*-EGFP^+^: one population (IFE I) showing clear features of undifferentiated IFE basal cells (*Krt14*^+^/*Ly6a*^+^ and enriched for cells in S phase) and the other one (IFE II) appearing as (*Krt10*^+^) committed IFE basal cells, in line with our observations above (Figures 2E–2H).

### *Troy*-expressing cells contribute to long-term IFE and INF maintenance

**Figure 5:**
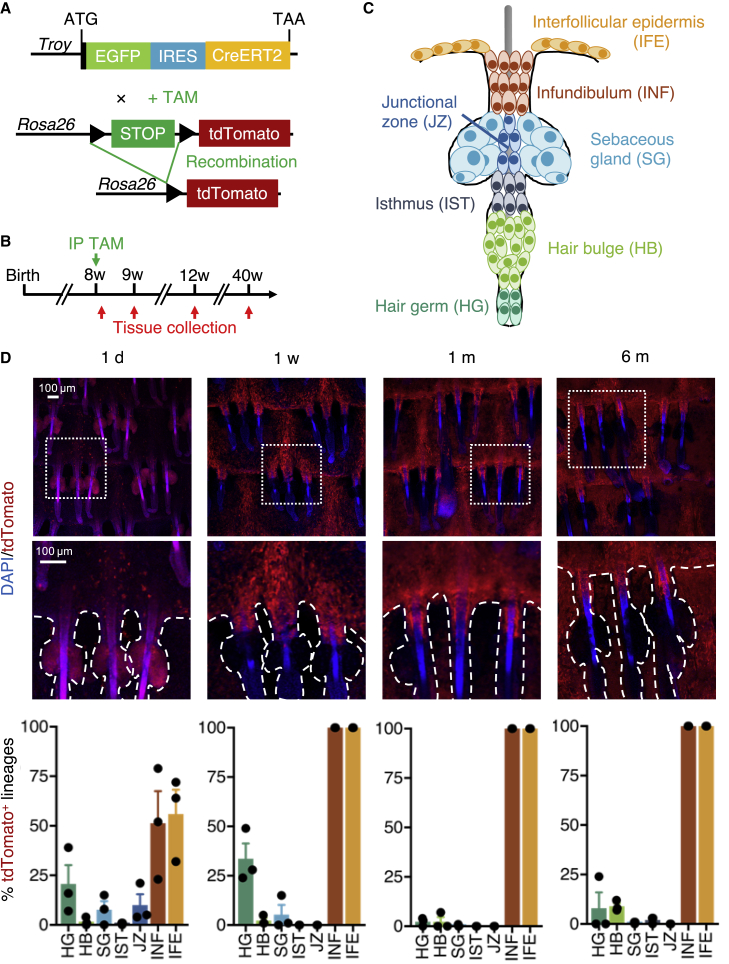
Genetic lineage tracing of *Troy*-expressing cells (A) Schematic representation of the genetic constructs. (B) Experimental timeline. (C) Schematic overview of a tail whole-mount hair follicle with indicated epidermal compartments. (D) Tail epidermal whole mounts following 1 day, 7 days, 1 month, or 6 months induction of lineage tracing stained for tdTomato (red) and counterstained with DAPI (blue). Column charts indicating quantification of lineage tracing over the time course. Twenty-five triplets containing hair follicles of different hair cycle stages were quantified for tdTomato clones (n = 2–3 mice). Data indicate mean ± SD. See also Figure S5.

To assess the long-term fate of *Troy*-expressing cells during homeostasis, we crossed *Troy*^EGFP-IRES-CreERT2^ mice with *Rosa26*-*loxP*-STOP-*loxP*-tdTomato (LSL-tdTomato) mice to allow for genetic lineage-tracing experiments (Kretzschmar and Watt, 2012) (Figure 5A). Double mutant mice received tamoxifen injections at the age of 7–9 weeks when back skin HFs are in telogen (Müller-Röver et al., 2001). Skin tissue of back, tail, ear, and paws was collected 1 day, 7 days, 1 month, and 6 months after tamoxifen injections (Figure 5B). We stained epidermal tail whole mounts for tdTomato and DAPI and scored the different epidermal compartments with tdTomato^+^ clones (Figure 5C). At 1 day and 1 week post tamoxifen, robust initial tdTomato labeling was found in the hair germ, IFE, and INF (Figure 5D). However, tracings for up to 6 months demonstrated that tdTomato^+^ clones primarly remained long term in IFE and INF only (Figure 5D). Next, we assessed whether *Troy*-expressing cells contributed to IFE homeostasis through generation of differentiated suprabasal progeny. We performed co-stainings for KRT14 and EGFP on sections of skin tissue collected from the tamoxifen-treated mice (Figure S5A). At 1-day post tamoxifen, an average of 2 KRT14^+^ IFE basal cells were tdTomato^+^, while no tdTomato labeling was found in the KRT14^–^ suprabasal layer (Figures S5A–S5C). After 1 week of lineage tracing, about 40 basal cells per field were tdTomato^+^, with additional tdTomato labeling of approximately 10 suprabasal keratinocytes per field (Figures S5A–S5C). Suprabasal tdTomato labeling increased further to 20 cells per field at 1 month post injection, while basal tdTomato labeling did not change significantly (Figures S5A–S5C). After 6 months of tracing, tdTomato labeling of both basal and suprabasal cells remained stable with no significant change compared with the labeling detected at 1 month post injection (Figures S5B and S5C). Similar long-term tracings were found in paw epidermis devoid of HFs, confirming that an epidermal stem cell population independent of the HF governs cellular input into the suprabasal layers of the cornifying epidermis (Figure S5D). In summary, these data demonstrate that *Troy*-expressing cells generate differentiating progeny long term, suggesting stem cell capacity of TROY^+^ cells *in vivo*.

### Characterization of the progeny of *Troy*-expressing cells using single-cell transcriptomics

**Figure 6:**
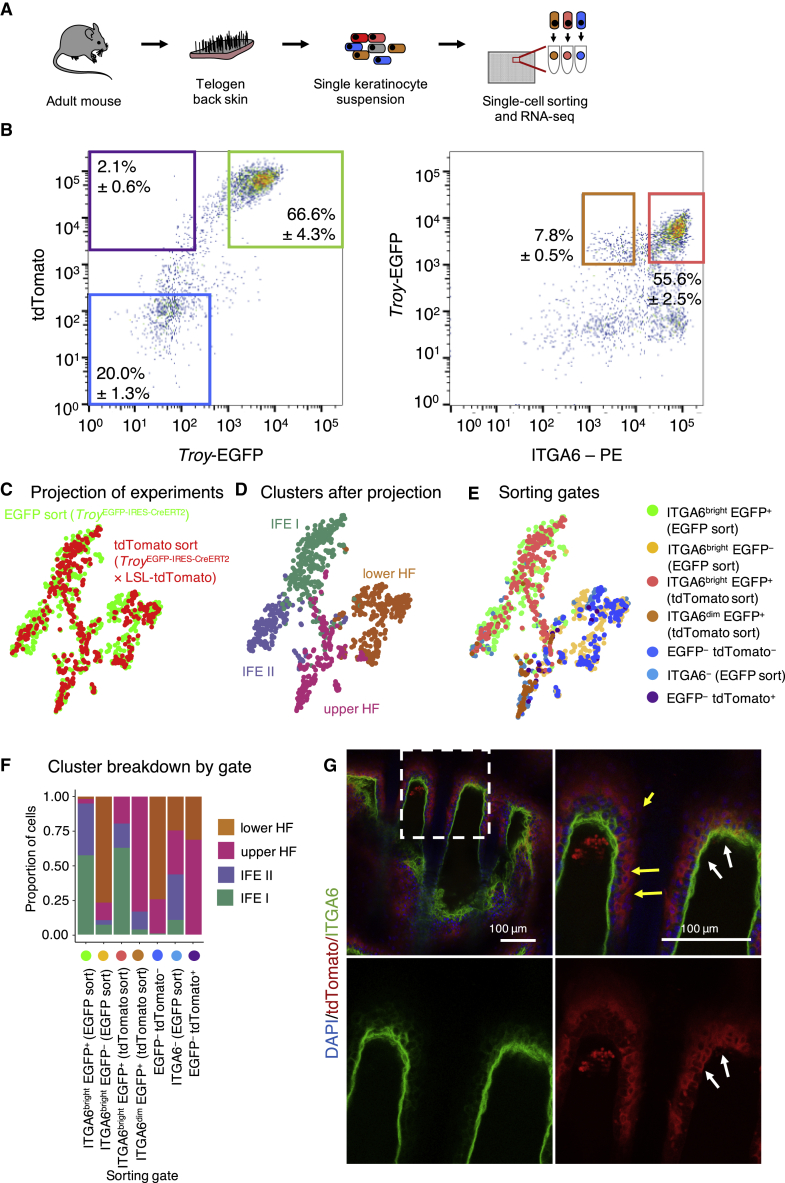
Characterization of progeny of *Troy*-expressing cells (A) Schematic overview of experimental outline. (B) Representative flow cytometry scatterplot of viable cells isolated from *Troy*^EGFP-IRES-CreERT2^ × LSL-tdTomato mice 7 days post induction of lineage tracing stained against ITGA6. Sorting gates are indicated in colors (for color coding, see (G)). (C–E) *t*-SNE map of single-cell sequencing data generated from 7 day induced mice (n = 2 mice) projected onto the dataset generated for Figure 4 C, with assigned clusters (D) and all sorting gates indicated (E). (F) Stacked column chart indicating cluster breakdown by gate. (G) Tail epidermal whole mount of a *Troy*^EGFP-IRES-CreERT2^ × LSL-tdTomato mouse 7 days post tamoxifen injection stained for tdTomato and ITGA6. Zoom-ins and arrows indicate suprabasal tracing (yellow arrows) or basal tracing (white arrows). See also Figure S6.

To further characterize the progeny of *Troy*-expressing cells at the transcriptional level, we first—for increased sensitivity to tamoxifen-induced Cre recombination—generated double homozygous *Troy*^EGFP-IRES-CreERT2^ × LSL-tdTomato knockin mice and then performed lineage tracing for 7 days (Figure 6A). We then prepared single-cell suspensions from adult telogen back skin and sorted the different cell populations based on their expression of ITGA6 (ITGA6^–^, ITGA6^dim^, and ITGA6^bright^), *Troy*-EGFP, as well as tdTomato (Figure 6B), and performed single-cell mRNA sequencing. Almost all *Troy*-EGFP^+^ cells also showed tdTomato^+^ expression, suggesting a labeling efficiency at saturating levels (>99%; Figure 6B). After quality control and filtering (Figure S6), we used the Seurat algorithm to project the newly generated single-cell dataset onto the initially analyzed *Troy*-EGFP dataset (Figures 4A–4K) based on matching cellular identities to the pre-defined clusters from our *Troy*-EGFP dataset (Figures 6C and 6D). Based on marker expression, the four clusters of undifferentiated *Krt14*^+^ IFE basal cells (IFE I), committed *Krt10*^+^ IFE basal cells (IFE II), *Postn*^+^ lower HF cells, and *Gata6*^+^ upper HF cells could be identified (Figure 6D). The majority of (tdTomato^+^) *Troy*-EGFP^+^ ITGA6^bright^ cells identified as undifferentiated *Krt14*^+^ IFE basal cells, while almost all (tdTomato^+^) *Troy*-EGFP^+^ ITGA6^dim^ cells were assigned to the cluster of *Gata6*^+^ upper HF cells (Figures 6D–6F). *Troy*-EGFP^–^ tdTomato^–^ cells were enriched in the cluster of *Postn*^+^ cells (Figures 6D–6F), suggesting that they originate from the lower HF.

These results are in line with the OFE assays (Figure 3), as both experiments demonstrated that *Troy*-EGFP^+^ ITGA6^bright^ cells have stem cell capacity, while tdTomato^+^ *Troy*-EGFP^–^ ITGA6^dim^ cells appear to be differentiating cells. To confirm these observations, we stained tail epidermal whole mounts collected from the mice sampled for these experiments for tdTomato and ITGA6 (Figure 6G). TdTomato^+^ cells were found in both the ITGA6^bright^ basal layer and the ITGA6^dim^ suprabasal layers of IFE and INF (Figure 6G), confirming the single-cell data. As *Troy*-EGFP^+^ cells were enriched in IFE and INF in telogen skin, tdTomato^+^ progeny of *Troy*-expressing cells was robustly identified as belonging to these lineages without significant contribution to other epidermal compartments (Figures 6D–6F), in line with the notion that epidermal stem cell niches are compartmentalized in adult homeostasis (Kretzschmar et al., 2016; Page et al., 2013). In conclusion, these data demonstrated the existence of a *Troy*-expressing cell population in the IFE/INF basal layer with stem cell capacity that readily contributes differentiating daugther cells to the cornifying layers of the epidermis.

## Discussion

Here, we identify *Troy* as a marker gene of epidermal cells that govern IFE and INF homeostasis. In telogen skin, *Troy*^+^ cells reside in the ITGA6^bright^ basal layer of IFE and INF. Genetic-tracing experiments demonstrate that progeny of *Troy*-expressing cells in these compartments of the so-called permanent portion of the epidermis contribute to cellular differentiation of cells and the cornified envelope long term, validating the stem cell capacity of *Troy*^+^ cells *in vivo*. Furthermore, *Troy*^+^ cells have a robust organoid-forming capacity, validating their stem cell potential *in vitro*.

We found human skin to be the tissue with the second highest expression levels of *TROY* among all human organs studied by the Human Protein Atlas. Using RNAscope technology, we also found that *TROY* transcripts were expressed by keratinocytes in the KRT14^+^ IFE basal layer and the developing HFs in fetal human skin, as well as in the KRT14^+^ IFE basal layer of adult human skin. Future studies may investigate the potential functional role of TROY^+^ epidermal cells in human skin. In murine telogen skin, *Troy* expression was restricted to the IFE and INF, the two cornifying epidermal compartments. However, during anagen, cells of the proliferative hair bulb were highly enriched for *Troy*, while low levels of *Troy* were also detected in the cells of the IRS that contribute to the keratinizing and hair shaft-producing layers of the HF. This suggests that the Wnt/β-catenin target gene *Troy* marks stem/progenitor cells collectively governing the cellular input into cornification and keratinization. A similar correlation was found in embryonic murine epidermis in line with previous studies demonstrating a functional (but redundant) role for TROY in HF morphogenesis (Kojima et al., 2000; Pispa et al., 2008).

Single-cell transcriptomics revealed that ITGA6^bright^ *Troy*-EGFP^bright^ IFE basal layer cells separate into two distinct cell populations. One population is defined by undifferentiated IFE basal cell markers genes, such as *Krt14*, *Krt5*, and *Ly6a* (IFE I), while the other population is enriched for transcripts of differentiation markers, such as *Krt1* and *Krt10* (IFE II), defining committed IFE basal cells. In line with our observations (Figures 2E–2H), a recent study found robust gene and protein expression of differentiation markers, such as KRT10, in the IFE basal layer (Cockburn et al., 2021). Using intravital imaging, the authors further demonstrated that almost all IFE basal cells (96%) expressing a KRT10 reporter exited the basal layer within 10 days of tracking. In addition, expression of differentiation markers has already been used to target committed cells in the epidermal basal layer by Mascré et al. (2012). In their paper, the authors demonstrate the existence of a population of committed epidermal basal layer cells that contribute to IFE homeostasis using a genetic lineage-tracing mouse model driven by terminal differentiation marker gene *Involucrin* (*Ivl*). Furthermore, the presence of committed epidermal cells in direct contact with the basement membrane through ITGA6—and therefore residing in the epidermal basal layer—is in line with the observations by Watt and colleagues showing that the basal layer of human epidermis is rather heterogeneous containing highly clonogenic cells marked by high levels of β1 integrin and cells with lower colony-forming efficiency and low β1 integrin expression (Jones et al., 1995; Jones and Watt, 1993; Tan et al., 2013).

Genetic lineage tracing confirmed that *Troy*-expressing cells in the IFE self-renew and produce differentiating progeny in the long term, independent of the HF, as demonstrated by tdTomato-labeling ranging from the basal layer to the cornified envelope of HF-free paw epidermis. These observations are in line with Lim et al. (2013), showing that stem cells located in the IFE act in a compartmentalized fashion separate from the influence of HFs in adult homeostasis. Interestingly, when an HF is attached to the IFE, such as in the epidermis of back, ear, and tail skin, *Troy* is expressed by both IFE and INF basal layer cells and long-term contribution to the cornified envelope can be found from both compartments. This finding is in line with Joost et al. (2016), showing that the basal cells of both epidermal compartments are transcriptionally rather similar.

Compartment-restricted activation of oncogenes, such as KRAS^G12D^, or inactivation of tumor suppressor mutations, such as p53^−/−^ in the IFE (and INF) as discussed previously (Blanpain, 2013), has been challenging due to lack of specific CreER mouse models (Lapouge et al., 2011). Although efforts were made to assess the ability of the IFE to form squamous cell carcinomas (SCCs) using *IVL*-CreERT2 transgenic mice to simultaneously ablate p53 and induce oncogenic KRAS, the results remained inconclusive, as *IVL*-CreERT2 targets IFE basal cells dedicated to differentiation and not long-term maintained stem cells (Mascré et al., 2012). Therefore, as *Troy* expression marks stem cells in the basal layer of the IFE and INF in telogen skin, the *Troy*-driven CreERT2 mouse model allows approaches like the testing of the origin of specific skin tumors, such as SCCs. Induction of CreER elsewhere in the mouse body can be avoided by local application of tamoxifen.

In addition, this mouse model could be applied in the study of basal cell dynamics in more depth during homeostasis and upon wounding. Epidermal stem cell populations have been shown to be highly plastic upon wounding, where stem cells from non-IFE or INF compartments replenish lost IFE stem cells and contribute to wound healing as well as long-term homeostasis after regeneration (Dekoninck and Blanpain, 2019). Despite the plastic behavior of stem cells upon wounding, it would be relevant to determine the proportion of IFE and INF stem cells contributing to wound healing and observe whether these dedicated IFE and INF stem cells can contribute to long-term tracing in newly formed HFs. Wounding studies in *Lgr6*^EGFP-IRES-CreERT2^ mice have shown the contribution of *Lgr6*-expressing cells to long-term maintenance of HFs; however, *Lgr6* is not exclusively expressed in the basal layer of the IFE and INF, but also in the HF junctional zone and SG (Füllgrabe et al., 2015; Kretzschmar et al, 2014, 2016; Page et al., 2013; Snippert et al., 2010). The *Troy* knockin mouse model therefore would allow a more refined tracing of IFE/INF basal cells in this context.

In conclusion, our study identifies epidermal basal cells in the IFE and INF with stem cell capacity marked by *Troy*. In contrast to other IFE-associated stem cell markers, such as *Axin2* and *Lgr6* (Füllgrabe et al., 2015; Lim et al., 2013), *Troy* is confined to the basal layer of the cornifying compartment and not robustly expressed in the SG or lower HF in telogen skin. With the characterization of *Troy*-expressing cells, opportunities arise to study disease and regenerative capacity specific to the basal layer of IFE and INF. Our data provide further evidence for cellular heterogeneity in the epidermal basal layer and show that *Troy* marks a lineage of basal cells including stem cells and those already committed to differentiation.

## Experimental procedures

### Human tissue

The use of human fetal scalp skin (16 weeks of gestation) was approved by the medial ethical committees of the LUMC (P08.087) and patient written informed consent was obtained beforehand. Human adult abdominal skin samples were obtained as discarded material after cosmetic surgery from anonymous donors who gave prior written informed consent for the use of material in research.

### Mouse lines

Mice were housed in the animal facility of the Hubrecht Institute and experiments were carried out under a Dutch government project license granted to Prof. Hans Clevers. The following experimental protocols were approved by the animal welfare committee of Utrecht University. Both male and female mice were used, except for experiments in Figure 6, where only male mice were used. Littermates were used as no-tamoxifen controls. Generation of *Mki67*^tagRFP^ expression cassettes (Basak et al., 2018), *Troy*^EGFP-IRES-CreERT2^ (Stange et al., 2013) and LSL-tdTomato mice (Madisen et al., 2010) was described elsewhere. Details are provided in the supplemental experimental procedures.

### Troy expression in human tissue

*TROY* consensus expression data were downloaded from v#19.proteinatlas.org (Human Protein Atlas) (Uhlén et al., 2015). Only tissues with normalized expression >2 were included.

### Murine epidermal keratinocyte isolation and flow cytometric purification

Isolation of keratinocytes from back skin was performed as described previously (Jensen et al., 2010). Isolated single cells were resuspended in FACS buffer (2 mM EDTA and 2% FBS in PBSO) at a density of 1 × 10^6^ cells per mL. Cells were stained on ice for 1 h with the following antibodies: rat anti-human/mouse ITGA6 (CD49f)-PE/Cy7 (555736, BD Biosciences, or 313621, BioLegend), rat anti-mouse Ly6A (SCA-1)-APC (17-5981-81, eBioscience) and rat anti-mouse CD34 (560230, BD Biosciences). Immediately before flow sorting, 4′,6-diamidino-2-phenylindole (DAPI) was added. Cells were either collected for bulk mRNA sequencing in TRIzol (Invitrogen) or for single-cell sequencing and sorted in 384-well format. All samples were stored at −80°C.

### Organoid experiments

For organoid culture experiments, cells were first isolated from back skin and sorted by flow cytometry based on several markers. Culture of murine epidermal organoids was performed as described previously (Boonekamp et al., 2019). Details are provided in the supplemental experimental procedures.

### RNA sequencing

RNA sequencing was performed using the CEL-Seq2 method (Hashimshony et al., 2016), as detailed in the supplemental experimental procedures.

### Histology

For paraffin sections, skin from the back, tails, ears, and paws was collected from *Troy*^EGFP-IRES-CreERT2^ mice and *Troy*^EGFP-IRES-CreERT2^ × LSL-TdTomato lineage-traced mice. Tissue was immediately fixed overnight in formalin at room temperature. Paw tissue was decalcified for at least 2 weeks in 10% EDTA after fixation. Procedures for paraffin embedding and stainings, tail whole-mount stainings, and RNAscope assays are described in the supplemental experimental procedures.

### Imaging

Tail whole-mount images and paraffin immunofluorescent images were acquired on a confocal microscope (Leica SP8X and SP8). Paraffin sections stained using immunohistochemistry were imaged on a Leica DM4000 microscope.

### Bioinformatics analysis

Sequencing, mapping to the mouse reference genome, and transcript counting of the DNA libraries were performed as described elsewhere (Kretzschmar et al., 2018). Bulk mRNA sequencing samples were analyzed using the DESeq2 package (Love et al., 2014). Single-cell mRNA sequencing libraries were analyzed using the Seurat v.3 package (Butler et al., 2018). All bioinformatics analyses were performed using R v.3.4.0 (R Foundation, https://www.r-project.org) and RStudio v.1.0.143 (https://www.rstudio.com). Details are provided in the supplemental experimental procedures.

### Data and code availability

The accession number for the sequencing data reported in this paper is Gene Expression Omnibus (GEO): GSE165379.

## Author contributions

K.K., K.E.B., and H.C. conceived the project, designed experiments, and interpreted results. K.K. and K.E.B. performed animal experiments. K.K., K.E.B., M.B., P.A., and M.K. performed all histology, imaging, and cell culture experiments. K.K. and K.E.B. performed sequencing experiments. K.K. performed bioinformatics analysis. S.M.C.d.S.L. and B.G. obtained human skin tissue. K.K. and H.C. acquired funding. K.K., K.E.B., and H.C. wrote the manuscript with input from all other authors.

## Conflicts of interest

The authors declare no competing interests. H.C. is the inventor on several patents related to organoid technology. He is cofounder of Surrozen, D1Med, and Xilis; board of directors-member of Roche/Genentech and SAB member of Volastra, Decibel, and Merus. His full disclosure is given at https://www.uu.nl/staff/JCClevers/.

## Supplemental information

Document S1. Supplemental experimental procedures and Figures S1–S6

Table S1. List of differentially expressed genes between *Troy*-EGFP^+^ and *Troy*-EGFP- ITGA6^bright^ epidermal basal layer cells, related to Figure 4

Document S2. Article plus supplemental information
