# Supplementary material for: Troy/Tnfrsf19 marks epidermal cells that govern interfollicular epidermal renewal and cornification
Source: Stem Cell Reports. 2021 Aug 5;16(9):2379–94. doi: 10.1016/j.stemcr.2021.07.007 (PMC8452520; doi:10.1016/j.stemcr.2021.07.007)
Supplement: Document S1. Supplemental experimental procedures and Figures S1–S6 [file mmc1.pdf]

Stem Cell Reports, Volume *16*

Supplemental Information

Troy/Tnfrsf19 marks epidermal cells that govern interfollicular epidermal renewal and cornification

Kai Kretzschmar, Kim E. Boonekamp, Margit Bleijs, Priyanca Asra, Mandy Koomen, Susana M. Chuva de Sousa Lopes, Barbara Giovannone, and Hans Clevers

Supplemental Information

Supplemental Figures

Figure S1, related to Figure 1

Figure S2, related to Figure 1

Figure S3, related to Figure 2

Figure S4, related to Figure 4

Figure S5, related to Figure 5

Figure S6, related to Figure 6

Supplemental Tables

Table S1, related to Figure 2

Supplemental Experimental Procedures

Supplemental References

Supplemental Figures

Figure S1

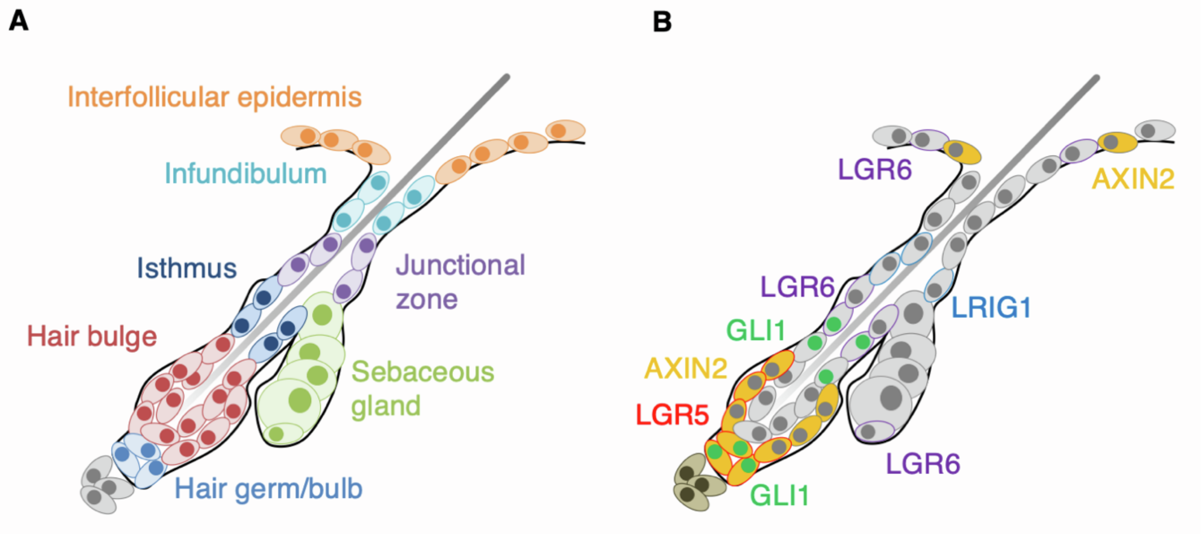

Figure S1. Epidermal compartments (A) and stem cell markers (B), related to Figure 1.

Figure S2

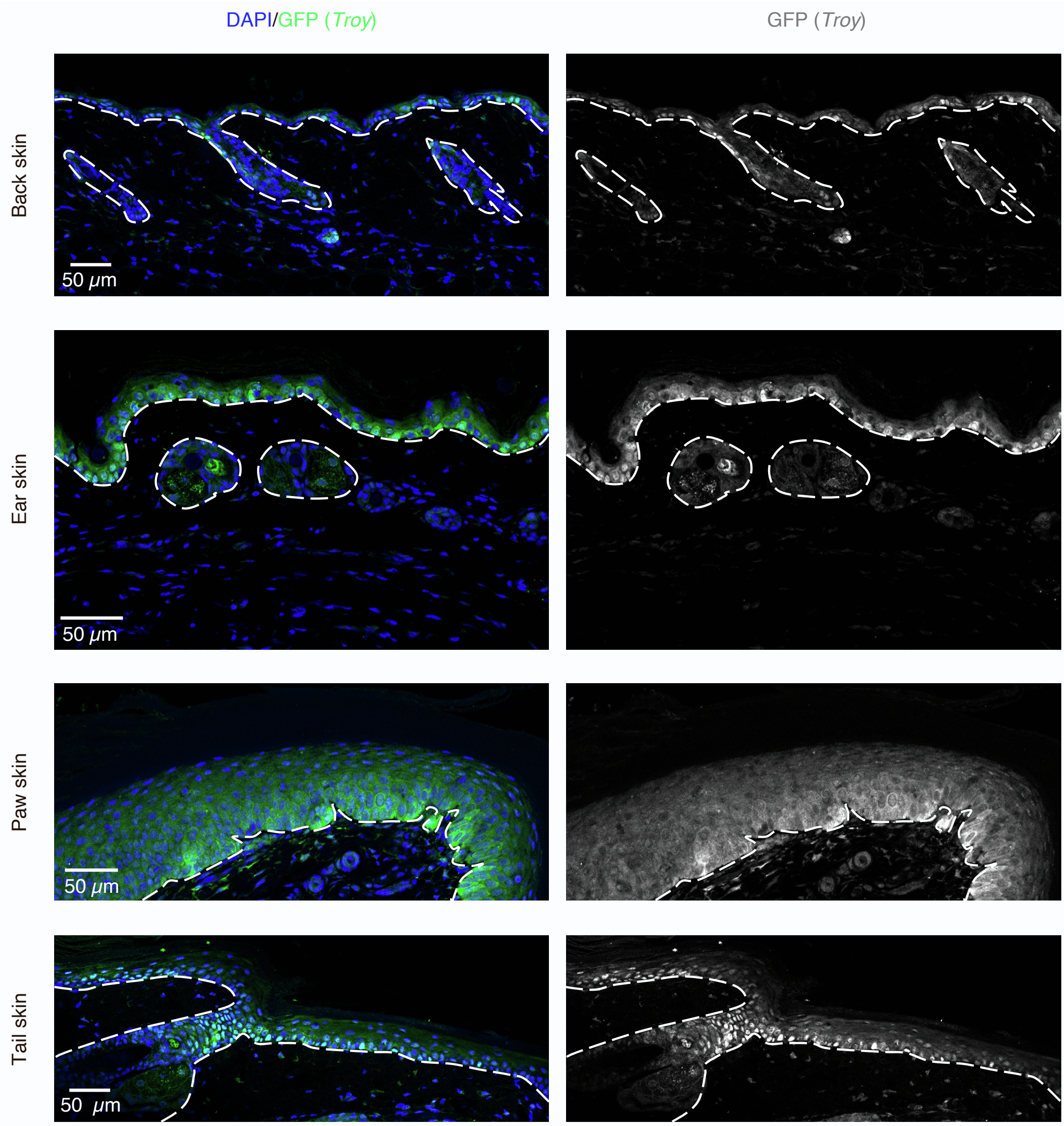

Figure S2. *Troy*-EGFP⁺ expression in adult murine skin, related to Figure 1.

Paraffin resections of mouse back, ear, paw and tail skin of adult (P50) *Troy*-EGFP⁺ mice. Sections were stained for EGFP (green/grey) and nuclei were counterstained with DAPI (blue).

Figure S3

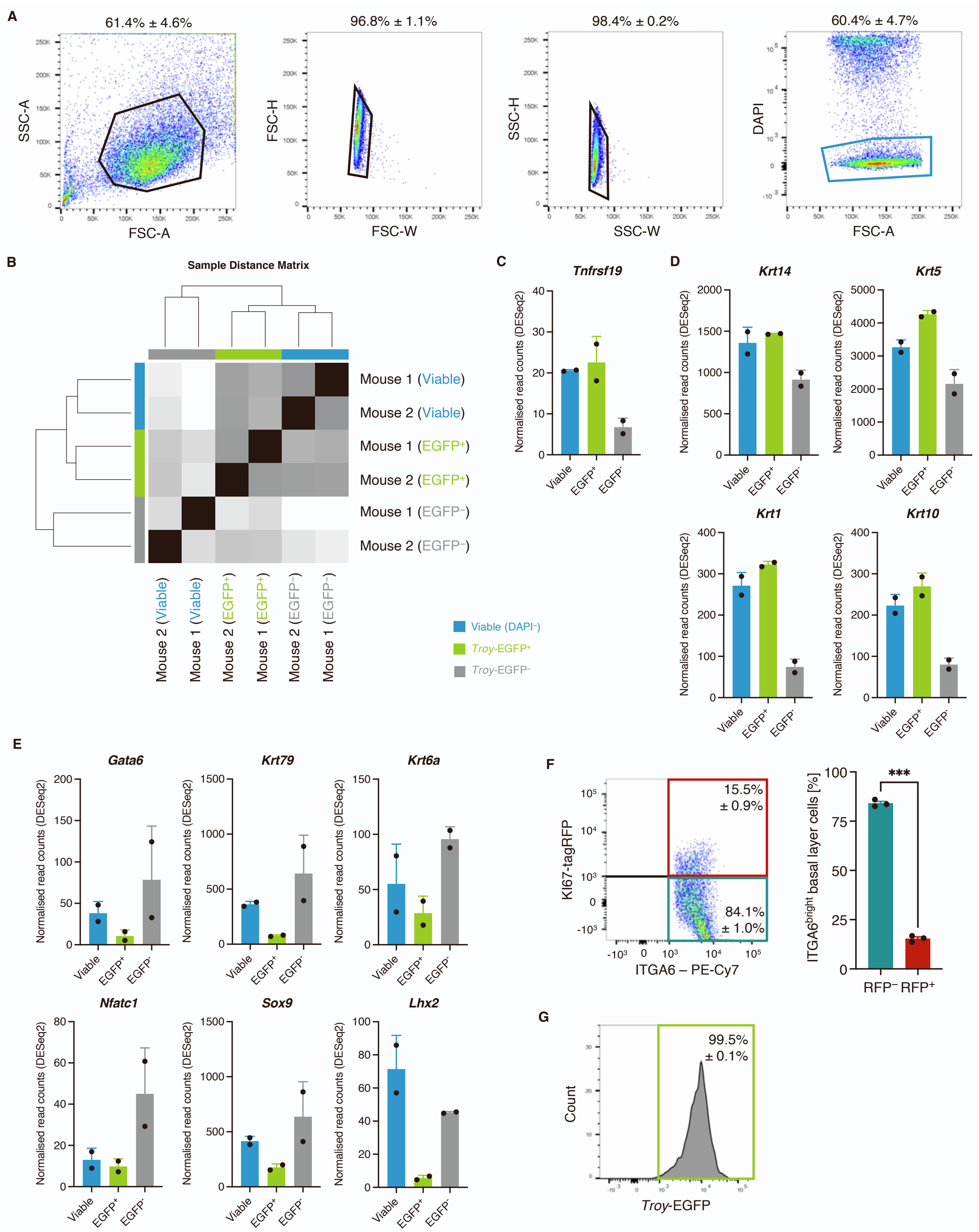

Figure S3. Sorting strategy and bulk mRNA-sequencing, related to Figure 2.

(A) Gating strategy.

(B) Sample distance matrix for the sorted populations used for bulk mRNA sequencing

(C-E) Column charts indicating normalised read counts (DESeq2) of *Troy* **(D),** IFE lineage markers (C) and HF lineage markers (E).

(F) Representative flow cytometry scatter plot of viable cells assessed for expression of ITGA6 and Ki67-tagRFP. Data in the gates indicate the percentage of cells per gate as mean average ± S.E.M. (*n* = 3 mice). Column chart indicating the percentage of Ki67-tagRFP^{-} and Ki67-tagRFP^{+} cells within the ITGA6^{bright} population. The data are presented as mean average ± S.E.M. (*n* = 3 mice). Dots represent the individual data points derived from each mouse.

(G) Histogram indicating the percentage of *Troy*-EGFP^{+} within the ITGA6^{high} Ki67-tagRFP^{+} cell population. The data are presented as mean ± S.E.M. (*n* = 3 mice).

Figure S4

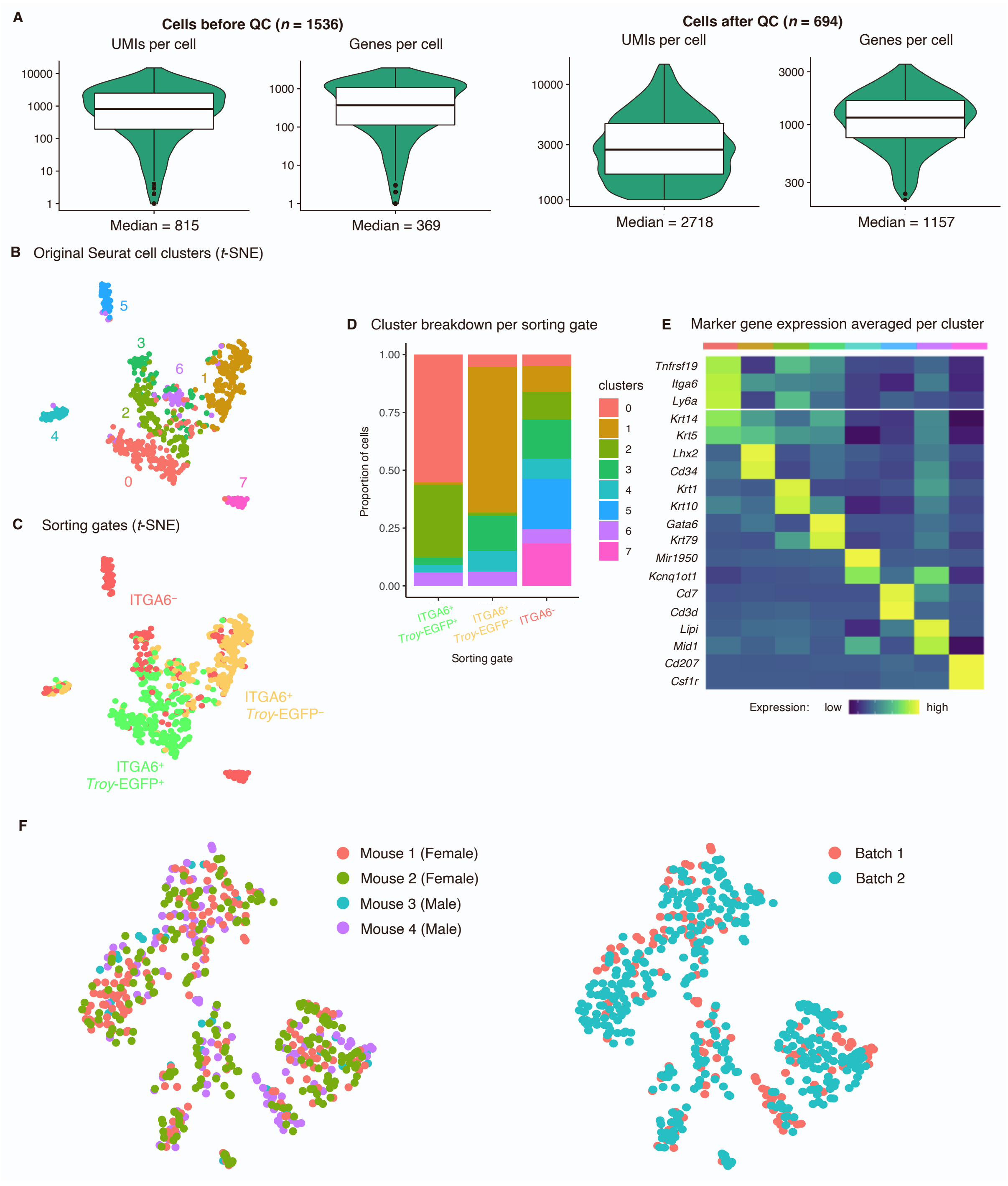

Figure S4. Quality metrics and initial clustering for scRNA-sequencing experiments using *Troy*$^{\text{EGFP-IRES-CreERT2}}$ mice, related to Figure 4.

(A) UMIs and genes per cell before and after quality control (QC).

(B) t-SNE plot indicating the 7 different clusters identified in the original clustering (clusters 0–7).

(C) *t*-SNE plot projecting the sorting gates on the original *t*-SNE map.

(D) Stacked column chart indicating cluster breakdown by gate.

(E) Heatmap implicating key marker gene expression averaged per cell population for all identified original clusters.

(F) *t*-SNE plot projecting different mice (left) and different sequencing batches (right).

Figure S5

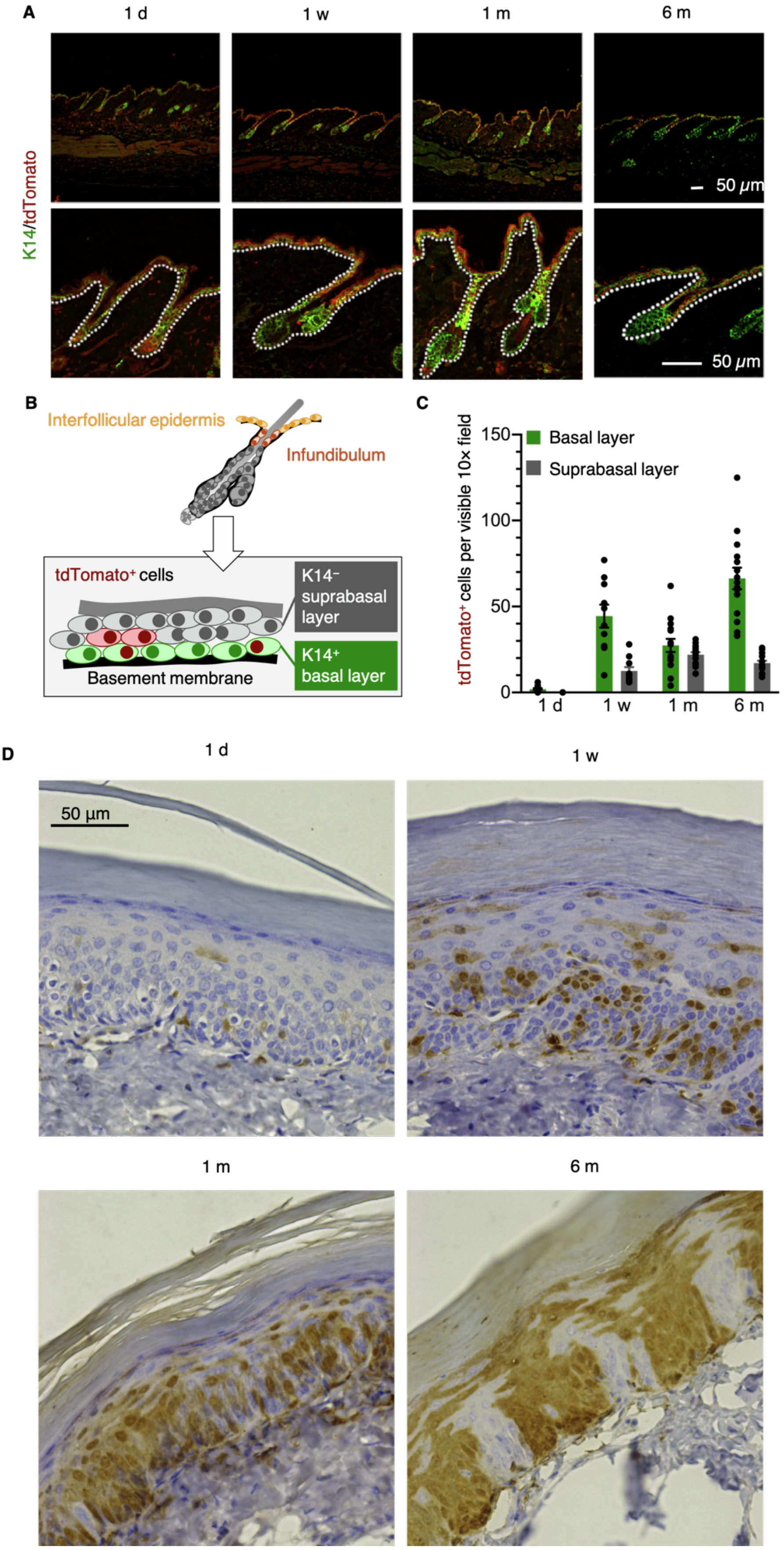
A
1 d
1 w
1 m
6 m
K14/tdTomato
50 μm
50 μm
B
Interfollicular epidermis
Infundibulum
tdTomato+ cells
K14− suprabasal layer
K14+ basal layer
Basement membrane
C
150
100
50
0
Basal layer
Suprabasal layer
tdTomato+ cells per visible 10× field
1 d
1 w
1 m
6 m
D
1 d
1 w
50 μm
1 m
6 m

Figure S5. Quantification of tdTomato labelling in the IFE basal and suprabasal layers, related to Figure 5.

(A) Paraffin slides of murine back skin of *Troy*$^{EGFP\text{-}IRES\text{-}CreERT2}$ x *Rosa26-LSL-tdTomato* mice induced with tamoxifen for indicated times stained against tdTomato (RFP; red) and keratin 14 (K14; green).

(B) Schematic overview of K14 expression in the IFE.

(C) Quantification of tdTomato cells in the basal layer (K14^{-}) or suprabasal layer (K14^{+})

(D) Paraffin slides of murine paw skin of *Troy*$^{EGFP\text{-}IRES\text{-}CreERT2}$ x *Rosa26-LSL-tdTomato* mice induced with tamoxifen for indicated times stained against tdTomato (RFP; brown) counter stained for nuclei with haematoxylin.

Figure S6

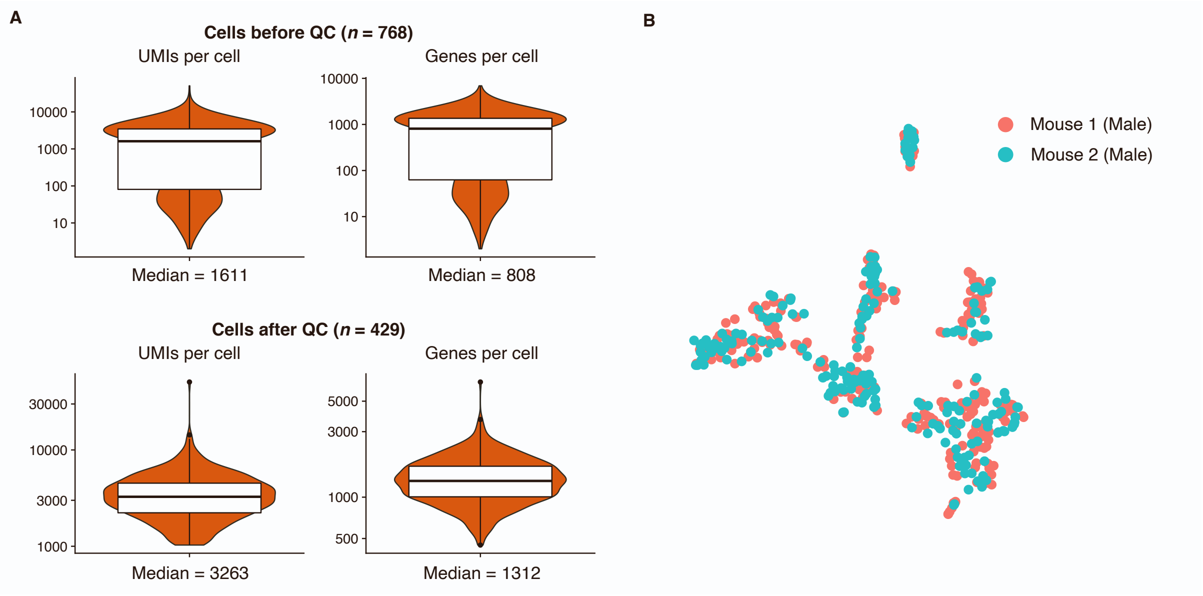

Figure S6. Quality metrics for scRNA-seq experiments using *Troy*$^{\text{EGFP-IRES-CreERT2}}$ x *Rosa26*$^{\text{LSL-tdTomato}}$ mice, related to Figure 6.

(A) UMIs and genes per cell before and after quality control (QC).

(B) *t*-SNE plot projecting different mice used in this experiment.

Supplemental Tables

Table S1. List of differentially expressed genes between *Troy*-EGFP^{+} and *Troy*-EGFP$^{–}$ ITGA6^{bright} epidermal basal layer cells, related to Figure 2.

Supplemental Experimental Procedures

Animal Experiments

In order to characterise *Troy* expression during epidermal development and HF cycling the following embryonic (E) and postnatal (P) days were collected: E12.5, E15.5, E17.5, P1, P2, P25, P42 and P49. For flow sorting experiments, both male and female mice of the age between 7 and 9 weeks were used.

For genetic lineage tracing experiments *Troy*$^{EGFP\text{-}IRES\text{-}CreERT2}$ mice were crossed to *Rosa26*$^{LSL\text{-}tdTomato}$ mice to label all progeny. All lineage tracing experiments were induced by a single injection of tamoxifen (Sigma) at 7–9 of weeks of age when the HFs were in telogen (resting phase). Tamoxifen was dissolved in sunflower oil (Sigma) and administered by IP (5 mg tamoxifen per mouse). Tissue was collected at 1 day, 7 days, 1 month and 6 months post tamoxifen injection.

Cell Lines

293T-HA-RspoI-Fc cells provided by Calvin Kuo, Standford University, Stanford, CA were grown at 5% CO_2 to generate R-spondin 1-conditioned medium (Drost et al., 2016). 293T-Noggin-Fc cells were grown at 5% CO_2 to generate Noggin-conditioned medium (Cattaneo et al., 2020; Heijmans et al., 2013).

Murine Epidermal Keratinocyte Isolation

Mice were sacrificed and their back skin was shaved. Back skin was isolated and washed in 70% EtOH twice and once in PBS without Ca^{2+} and Mg^{2+} (PBSO). Subsequently, the subcutaneous fat was removed from the dermal side by scraping with a scalpel. Post-cleaning, the tissue was incubated with the epidermal side up in freshly prepared 0.025% trypsin in Advanced Dulbecco's Modified Eagle Medium (DMEM)/F12 overnight at 4 ˚C. The following day, the tissue was transferred to a clean dish and the cells were scraped from the epidermal side using a scalpel. After mincing the tissue using scalpels, the cells were resuspended in 20 ml DMEM and pipetted vigorously up and down for 20 times in a 50 ml conical tube. After that cells were filtered through a 70-μm cell strainer and separated over 2 individual 15 ml conical tubes and centrifuged at 300×*g* for 5 minutes at 4 °C. Subsequently, cells were washed twice in DMEM.

Organoid Experiments

Flow cytometry purified cells were seeded in BME in a density of 2500 cells per 10 µl BME in a 48-well plate in triplicate. Organoids were grown for 7 days at 37 °C in epidermal expansion medium (EEM): Advanced DMEM/F12 supplemented with penicillin/streptomycin (100 U/l), Hepes, (10 mM), GlutaMAX (1×), B27 supplement (1×), N-Acetylcysteine-1 (1 mM), Noggin-conditioned medium (5%), R-spondin 1-conditioned medium (5%), acidic FGF1 (100 ng/ml), Heparin (0.0001%), Forskolin (10 ng/ml), Rho kinase inhibitor (Y-27632; 10 µM), and Primocin (1×). Subsequently, organoid cultures were imaged using a brightfield microscope (n = 3). Quantification of organoid number and size was performed in ImageJ using the "analyse particles" tool, all colonies and organoids were manually outlined and colony/organoid size and number was noted. Thereafter, imaged organoids were lysed with CellTiter-Glo® 3D Cell Viability Assay reagent (Promega, 1:1 diluted with Advanced DMEM/F12 +++) for 20 minutes at room temperature while shaking. Luminescence was detected using a Berthold reader.

RNA Sequencing

mRNA sequencing samples were processed according to the CEL-Seq2 method (Hashimshony et al., 2016). For bulk mRNA-sequencing, after storage at –80 °C, RNA was extracted from the bulk sorted samples by standard TRIzol extraction protocols followed by RNA precipitation with 2 μg GlycoBlue (Ambion) overnight at –80 °C as described elsewhere (Kretzschmar et al., 2018). Post overnight incubation, bulk RNA samples were labelled with UMI barcode primers in a reverse transcription reaction mix (Invitrogen). Subsequently, samples were pooled into specific libraries and processed for submission. For single-cell mRNA-sequencing following the SORT-seq method (Muraro et al., 2016),

cells were directly sorted in lysis buffer containing specific primers. After storage in –80 °C RNA the reactions were subjected to first strand and second strand synthesis. Subsequently, samples were pooled into plate-specific libraries and further processed for submission (Muraro et al., 2016). Amplified RNA for bulk sequencing and single-cell sequencing libraries were used to generate complementary (cDNA) libraries using Illumina TruSeq primers. All submitted libraries were sequenced on an Illumina NextSeq500 using 75bp pair-end sequencing with high output (150 million reads per run).

Histology

Fixed tissue was subjected to subsequent dehydration steps and embedded in paraffin blocks. Paraffin blocks were sectioned to generate sections of 4 μm. Sections were re-hydrated following conventional protocols and subjected to immunohistochemistry staining against either Keratin 14 (clone LL002, Thermo Fisher Scientific), EGFP (ab13970, Abcam) or tdTomato (600-401-379, Rockland). In short, antigen retrieval was performed by boiling slides in citrate buffer pH 6. Sections were stained with primary antibody overnight at 4 °C and incubated with secondary antibody for 2 hours at room temperature. For immunohistochemistry, secondary antibodies conjugated to HRP were used followed by development using 3,3'-diaminobenzidine (DAB). Slides were counterstained with haematoxylin, dehydrated and mounted using Pertex. For immunofluorescence, secondary antibodies conjugated to a fluorophore were used, followed by counterstaining using DAPI and mounting using Vectashield (Vectorlabs).

For tail whole mount stainings, tails were processed according to the protocol described by Braun and colleagues (2003). In short, tail epidermis was separated from the dermis following 4-hour incubation in 5 mM EDTA at 37 °C. Tail whole mounts were fixed in 4% paraformaldehyde for 15 minutes at room temperature and stored at 4 °C in PBSO + 0.02 % Azide. For tail whole mount staining, tails were first incubated in blocking buffer (1x TBS, 0.5 % (v/v) TritonX-100, 0.25% (v/v) fish skin gelatine, 0.5% (w/v) milk powder) for an hour at room temperature. All steps were performed protected from light. Primary antibody was incubated overnight at room temperature followed by washes in PBSO + 0.02 % Tween (PBS-T) the next day. Primary antibodies were: EGFP (ab13970, Abcam), mouse anti-Human Ki67 (Clone B56, 550609, BD Pharmingen), rat anti-human/mouse CD49f-PE (clone GoH3, 555736, BD Biosciences) and anti-tdTomato (600-401-379, Rockland). Secondary antibody (Thermo Scientific) was incubated overnight at room temperature followed by washes in PBSO the next day. Tail whole mounts were counterstained with DAPI for 15 minutes at room temperature followed by a wash with MilliQ water. Tail whole mounts were mounted on slides using ProLong gold (Invitrogen). Stained sections were stored at 4 °C in the dark until imaging.

In order to look at expression of human *TROY* we conducted RNAscope assays (Wang et al., 2012). RNA scope analysis was performed on paraffin-embedded human foetal and adult skin. Sectioning was performed maximum 1 week ahead of staining and slides where dried overnight at room temperature. RNAscope was performed according to the manufacture outlines. All paraffin sections of human skin where subjected to a 15-minute boiling step in target retrieval buffer and a 30-minute incubation at 40 °C with RNAscope protease plus reagent. Pre-treated slides were incubated for 2 hours at 40 °C with RNAscope Probe Hs TNFRSF19 (Channel 1) (441931, ACD Biotechne). Post washing, amplification trees were built according to manufactures guidelines and the *TROY*-specific signal was developed and visualized with Opal 570 fluorophores (FP14880001KT, Akoya). Slides were either co-stained with DAPI and mounted in ProLong Gold directly or subjected to a complementary immunostaining against keratin 14 (1:100; Thermo Scientific, LL002) according to manufactures guidelines followed by DAPI counter staining and mounting in ProLong Gold. Paraffin sections of P50 murine back skin were processed similarly to the human sections and were stained with RNAscope Probes Ms Tnfrs19 (Channel 1 – Opal 570) (420241 ACD Biotechne) and Ms Keratin 10 (Channel 2 – Opal 690) (457901-C2, ACD Biotechne).

Bioinformatics Analysis

Bulk mRNA sequencing samples were analysed using the DESeq2 package (Love et al., 2014) with standard parameters in R/Bioconductor environment. Raw read counts were normalised using the DESeq2 median of ratios method and these normalised read counts were used to generate the column charts depicted in Figure S3C-E. Differentially expressed (DE) genes (see Figure 2D and Table S1) were identified from the normalised read count table using DESeq2 with a cut-off of adjusted $p < 0.05$.

The sample distance matrix was generated after `rlog` transformation. Heatmaps in Figures 2D and S3B were generated using gplots' `heatmap.2` function.

Single-cell mRNA sequencing libraries were analysed using the Seurat v3 package (Butler et al., 2018) in R/Bioconductor environment. ERCC92 spike-ins and mitochondrial genes and cell transcriptomes with less than 1,000 transcript UMIs were removed from the dataset (Figures S4 and S6). The remaining transcriptomes were normalised using Seurat's `SCTransform` function using the `vars.to.regress` setting to remove confounding sources of variation such as total UMI counts, total gene counts and sequencing run. For initial cell type analysis, cell clusters were generated based on gene expression similarities using Seurat's `FindClusters` at a `resolution = 0.8` (Figure S4B). Marker gene expression per cluster was averaged using Seurat's `AverageExpression` function and plotted using Seurat's `DoHeatmap` function to generate Figures 4H and S4E. For the further analysis cell cluster enriched for necrotic cell markers such as *Mir1950* and *Kcqn1ot1* (Kretzschmar et al., 2018) or enriched for non-epidermal cells marked by *Cd7*, *Cd3d*, *Lipi*, *Mid1*, *Cd207* and/or *Csf1r* were removed (Figure 4). Cell cycle analysis (Figure 4F) was performed using Seurat's `CellCycleScoring` function. The transcriptome dataset collected after lineage tracing using *Troy*$^{EGFP\text{-}IRES\text{-}CreERT2}$ × *Rosa26*-LSL-tdTomato mice (Figure 6) was projected onto the initial *Troy*$^{EGFP\text{-}IRES\text{-}CreERT2}$ transcriptome dataset using Seurat's `FindTransferAnchors` function after quality control, filtering and removal of necrotic and non-epidermal cells.

Statistics

Statistically significant differences were determined using two-tailed Student's tests unless stated otherwise. Statistically analyses were performed using GraphPad Prism version 9.1.0, except for the statistics performed during bioinformatics analyses.

Supplemental References

Braun, K.M., Niemann, C., Jensen, U.B., Sundberg, J.P., Silva-Vargas, V., and Watt, F.M. (2003). Manipulation of stem cell proliferation and lineage commitment: visualisation of label-retaining cells in wholemounts of mouse epidermis. Development *130*, 5241–5255.

Butler, A., Hoffman, P., Smibert, P., Papalexi, E., and Satija, R. (2018). Integrating single-cell transcriptomic data across different conditions, technologies, and species. Nat. Biotechnol. *36*, 411–420.

Cattaneo, C.M., Dijkstra, K.K., Fanchi, L.F., Kelderman, S., Kaing, S., van Rooij, N., van den Brink, S., Schumacher, T.N., and Voest, E.E. (2020). Tumor organoid–T-cell coculture systems. Nat. Protoc. *15*, 15–39.

Drost, J., Karthaus, W.R., Gao, D., Driehuis, E., Sawyers, C.L., Chen, Y., and Clevers, H. (2016). Organoid culture systems for prostate epithelial and cancer tissue. Nat. Protoc. *11*, 347–358.

Hashimshony, T., Senderovich, N., Avital, G., Klochendler, A., de Leeuw, Y., Anavy, L., Gennert, D., Li, S., Livak, K.J., Rozenblatt-rosen, O., et al. (2016). CEL-Seq2 : sensitive highly-multiplexed single-cell RNA-Seq. Genome Biol. *17*, 1–7.

Heijmans, J., van Lidth de Jeude, J.F., Koo, B.K., Rosekrans, S.L., Wielenga, M.C.B., van de Wetering, M., Ferrante, M., Lee, A.S., Onderwater, J.J.M., Paton, J.C., et al. (2013). ER stress causes rapid loss of intestinal epithelial stemness through activation of the unfolded protein response. Cell Rep. *3*, 1128–1139.

Kretzschmar, K., Post, Y., Bannier-Hélaouët, M., Mattiotti, A., Drost, J., Basak, O., Li, V.S.W., van den Born, M., Gunst, Q.D., Versteeg, D., et al. (2018). Profiling proliferative cells and their progeny in damaged murine hearts. Proc. Natl. Acad. Sci. *115*, E12245 LP-E12254.

Love, M.I., Huber, W., and Anders, S. (2014). Moderated estimation of fold change and dispersion for RNA-seq data with DESeq2. Genome Biol. *15*, 550.

Muraro, M.J., Dharmadhikari, G., Grün, D., Groen, N., Dielen, T., Jansen, E., van Gurp, L., Engelse, M.A., Carlotti, F., de Koning, E.J.P., et al. (2016). A single-cell transcriptome atlas of the human pancreas. Cell Syst. *3*, 385-394.e3.

Wang, F., Flanagan, J., Su, N., Wang, L., Bui, S., Nielson, A., Wu, X., Vo, H.-T., Ma, X.-J., and Luo, Y. (2012). RNAscope: a novel in situ RNA analysis platform for formalin-fixed, paraffin-embedded tissues. J. Mol. Diagnostics *14*, 22–29.
