# Supplementary material for: Troy/Tnfrsf19 marks epidermal cells that govern interfollicular epidermal renewal and cornification
Source: Stem Cell Reports. 2021 Aug 5;16(9):2379–94. doi: 10.1016/j.stemcr.2021.07.007 (PMC8452520; doi:10.1016/j.stemcr.2021.07.007)
Supplement: Document S2. Article plus supplemental information [file mmc3.pdf]

Stem Cell Reports
Article
OPEN ACCESS

Troy/Tnfrsf19 marks epidermal cells that govern interfollicular epidermal renewal and cornification

Kai Kretzschmar,[1,2,7,*] Kim E. Boonekamp,[1,3,7] Margit Bleijs,[1,4] Priyanca Asra,[1] Mandy Koomen,[1] Susana M. Chuva de Sousa Lopes,[5] Barbara Giovannone,[6] and Hans Clevers[1,4,*]

[1]Oncode Institute, Hubrecht Institute, Royal Netherlands Academy of Arts and Sciences (KNAW) and University Medical Centre (UMC) Utrecht, 3584 CT Utrecht, the Netherlands
[2]Mildred Scheel Early Career Centre (MSNZ) for Cancer Research Würzburg, University Hospital Würzburg, 97080 Würzburg, Germany
[3]German Cancer Research Centre (DKFZ), 69120 Heidelberg, Germany
[4]Princess Máxima Center for Pediatric Oncology, 3584 CS Utrecht, the Netherlands
[5]Leiden UMC, 2333 ZC Leiden, the Netherlands
[6]UMC Utrecht, 3584 CX Utrecht, the Netherlands
[7]Co-first author
*Correspondence: kai.kretzschmar@uni-wuerzburg.de (K.K.), h.clevers@hubrecht.eu (H.C.)
https://doi.org/10.1016/j.stemcr.2021.07.007

SUMMARY

The skin epidermis is a highly compartmentalized tissue consisting of a cornifying epithelium called the interfollicular epidermis (IFE) and associated hair follicles (HFs). Several stem cell populations have been described that mark specific compartments in the skin but none of them is specific to the IFE. Here, we identify *Troy* as a marker of IFE and HF infundibulum basal layer cells in developing and adult human and mouse epidermis. Genetic lineage-tracing experiments demonstrate that *Troy*-expressing basal cells contribute to long-term renewal of all layers of the cornifying epithelium. Single-cell transcriptomics and organoid assays of Troy-expressing cells, as well as their progeny, confirmed stem cell identity as well as the ability to generate differentiating daughter cells. In conclusion, we define *Troy* as a marker of epidermal basal cells that govern interfollicular epidermal renewal and cornification.

INTRODUCTION

Mammalian skin acts as a protective mechanical and biological barrier against injuries, foreign pathogens, and loss of heat and water. Critical to the skin's main function is its outermost layer, the epidermis, which is comprised of a multi-layered epithelium, the interfollicular epidermis (IFE), and associated hair follicles (HFs), sebaceous glands (SGs), and sweat glands (in mice only in the paws). Directly exposed to the body's outside is the cornified envelope of the epidermis, also known as the stratum corneum. This epidermal layer consists of enucleated, organelle-free cells, which are enriched in highly crosslinked filamentous keratins and other cytoskeletal proteins in their cytoplasm. The cornified envelope is mainly generated by the IFE. The stratified squamous epithelium of the IFE is heavily studied using cultured human and murine keratinocytes or mouse models to explore adult homeostasis and perturbations, such as wounding or diseases.

Pioneering work by Rheinwald and Green used single human epidermal cells, so-called keratinocytes, cultured on a layer of inactivated mouse fibroblasts acting as feeder cells. This approach established in the mid-1970s that human epidermis contains cells with the capacity to generate a stratified squamous epithelium *in vitro* and, hence, show stemness potential (Rheinwald and Green, 1975). Subsequently, starting from the—now contested—notion that adult stem cells are DNA-label-retaining cells, epidermal stem cells were thought to be located to a specialized niche in the HFs in mouse and human epidermis, called the bulge (Cotsarelis et al., 1990). Genetic lineage-tracing experiments performed in mice have since provided evidence for the presence of numerous stem cell populations in the adult epidermis contributing to tissue homeostasis and regeneration (Figures S1A and S1B). Stem cells in the lower HF (bulge and hair germ) are marked by *Axin2*, *Cd34*, *Gli1*, *Krt15*, *Krt19*, *Lgr5*, and *Sox9* (Brownell et al., 2011; Jaks et al., 2008; Kadaja et al., 2014; Kretzschmar et al., 2016; Lim et al., 2016; Morris et al., 2003; Nowak et al., 2008). Stem cells in the upper HF (isthmus, junctional zone, and infundibulum) are marked by *Gli1*, *Lgr6*, *Lrig1*, and *Plet1*/MTS24 (Brownell et al., 2011; Füllgrabe et al., 2015; Jensen et al., 2009; Kretzschmar et al., 2016; Nijhof et al., 2006; Page et al., 2013; Raymond et al., 2010; Snippert et al., 2010). Stem cells in the SGs are marked by *Lgr6* and *Lrig1* and stem cells in the IFE are marked by *Axin2* and *Lgr6* (Füllgrabe et al., 2015; Kretzschmar et al., 2016; Lim et al., 2013; Page et al., 2013). Collectively, this suggests that marker genes are typically not specific to one epidermal compartment. Throughout the epidermis, stem cells are located to the bottom-most layer of the epithelium, the basal layer also known as stratum basale, which is marked by the basal keratins KRT5 and KRT14 and is in direct contact to the basement membrane (Fuchs and Weber, 1994). Extracellular matrix proteins, such as laminins and collagens, are highly enriched in the basement membrane and bind integrins, such as α6 integrin (ITGA6) and β1

© 2021 The Author(s).
This is an open access article under the CC BY license (http://creativecommons.org/licenses/by/4.0/).

integrin (ITGB1) that are highly expressed by epidermal basal layer cells in humans and mice (Jones et al., 1995; Jones and Watt, 1993; Kretzschmar and Watt, 2014). While many markers of human epidermal stem and progenitor cells have been proposed, a common marker shared by such cells contributing to IFE differentiation in both humans and mice is lacking.

Canonical Wnt signaling plays a critical role in epidermal development, homeostasis, and regeneration (Kretzschmar and Clevers, 2017). Interestingly, a great number of murine epidermal stem cell markers are bona fide Wnt/β-catenin target genes and also found to be specific to adult stem cells in other epithelia. *Lgr5*, for example, has initially been defined as a marker gene of intestinal epithelial stem cells and was later found to be expressed by adult stem cells in the lower HF and various other epithelial tissues throughout the body (Barker et al., 2007, 2010; Huch et al., 2013; Jaks et al., 2008). Based on this observation, we aimed to explore the potential role of TROY in embryonic and adult mouse epidermis. TROY is expressed by a Wnt/β-catenin target gene also known as tumor necrosis factor receptor superfamily, member 19 (*TNFRSF19* or *TROY* in humans and *Tnfrsf19* or *Troy* in mice) and has already been defined to mark adult stem cells in the intestinal and gastric epithelium as well as in adult neuronal stem cells (Basak et al., 2018; Fafilek et al., 2013; Stange et al., 2013).

Expression of TROY in skin has been described during skin (embryonal and neonatal) development, suggesting a potential role there (Kojima et al., 2000). In addition, *Troy* was found to be enriched in the basal layer of the infundibulum (INF) and IFE through single-cell transcriptomics on adult murine skin (Joost et al., 2016). However, no obvious skin phenotype has been demonstrated for TROY-deficient (*Troy*$^{+/-}$) mice (Pispa et al., 2008). TROY shares homology with other TNF receptor members called EDAR and XEDAR, suggesting possible functional redundancy (Hashimoto et al., 2008; Kojima et al., 2000). *Troy*$^{-/-}$*Eda*$^{-/-}$ mice lacking expression of TROY as well as EDA, the ligand of EDAR and XEDAR, show strong defects in HF development (Pispa et al., 2008) revealing an important role for TROY/EDA signaling during skin development. Knowledge on the role of TROY^{+} cells in adult skin is missing. Here, we therefore map TROY expression in embryonic, neonatal, and adult skin, and assess the contribution of TROY^{+} cells to epidermal adult homeostasis.

RESULTS

TROY marks interfollicular and infundibular epidermal cells in telogen skin

Based on consensus data generated by the Human Protein Atlas program (Uhlén et al., 2015), we found that the skin was the human tissue with the second highest normalized expression of *TROY* (Figure 1A). We next performed RNAscope analyis on paraffin sections of both fetal and adult human skin, which allows for the visualization of mRNA transcripts in intact cells (Wang et al., 2012). We found *TROY* mRNA transcripts in the epidermis of both human fetal scalp and adult abdominal skin (Figures 1B and 1C). In human fetal scalp skin, *TROY* was mostly confined to the KRT14^{+} epidermal basal layer and developing HFs with a notable enrichment of transcripts in the HFs (Figure 1B). In human adult abdominal skin, *TROY* mRNA transcripts were more widely detected across the stratified epithelium, including its KRT14^{+} cell layers (Figure 1C).

To determine the localization of TROY expression in murine skin, we used the *Troy*$^{\text{EGFP-IRES-CreERT2}}$ mouse model (Stange et al., 2013). In this model, an expression cassette harboring enhanced green fluorescent protein (EGFP) and Cre recombinase fused to a tamoxifen inducible, mutated version of the human estrogen receptor (CreERT2) is knocked into the *Troy* locus replacing its protein-coding region (Figure 1D). We collected tissue from different skin regions, including back and tail at different time points of development and homeostasis and stained for EGFP as a proxy for TROY expression (Figure 1E). At embryonic day 12.5 (E12.5), when the epidermis is an undifferentiated layer between the periderm and dermis, EGFP expression in back skin was confined to epidermal cells (Figure 1F). As development progresses, at E16.5, we found EGFP immunoreactivity specific to the IFE basal layer and developing HFs (Figure 1G). EGFP expression was most prominent in the areas of the hair placode as well as in the hair shaft-forming regions of the hair germ and peg (Figure 1G), while EGFP positivity was also detectable in the dermal condensate (Figure 1G). In neonatal skin, at post-natal day 1 (P1), when the HF forms as a bulbous peg, EGFP expression remained present in the IFE basal layer as well as in the hair bulb and hair shaft-forming inner root sheath (IRS) (Figure 1H). However, EGFP expression was absent from the outer root sheath as well as the upper HF portion generating the isthmus, junctional zone and SG (Figure 1H). Next, we stained epidermal whole mounts (Braun et al., 2003) collected from the tail skin of *Troy*$^{\text{EGFP-IRES-CreERT2}}$ mice at P50 and stained for EGFP (Figures 1I–1K). EGFP expression was confined to the IFE and INF (Figures 1J and 1K) as well as the hair bulb and IRS of anagen HFs (Figures 1I and 1K). Sections of various adult murine skin tissues stained for EGFP showed robust immunoreactivity in the basal layer of IFE and INF of back, ear, and tail, as well as the epidermis of paw skin, which is devoid of HFs (Figure S2). These results confirmed *Troy* expression throughout HF development and showed confined *Troy* expression in the IFE (and INF associated with telogen HFs) in adult skin.

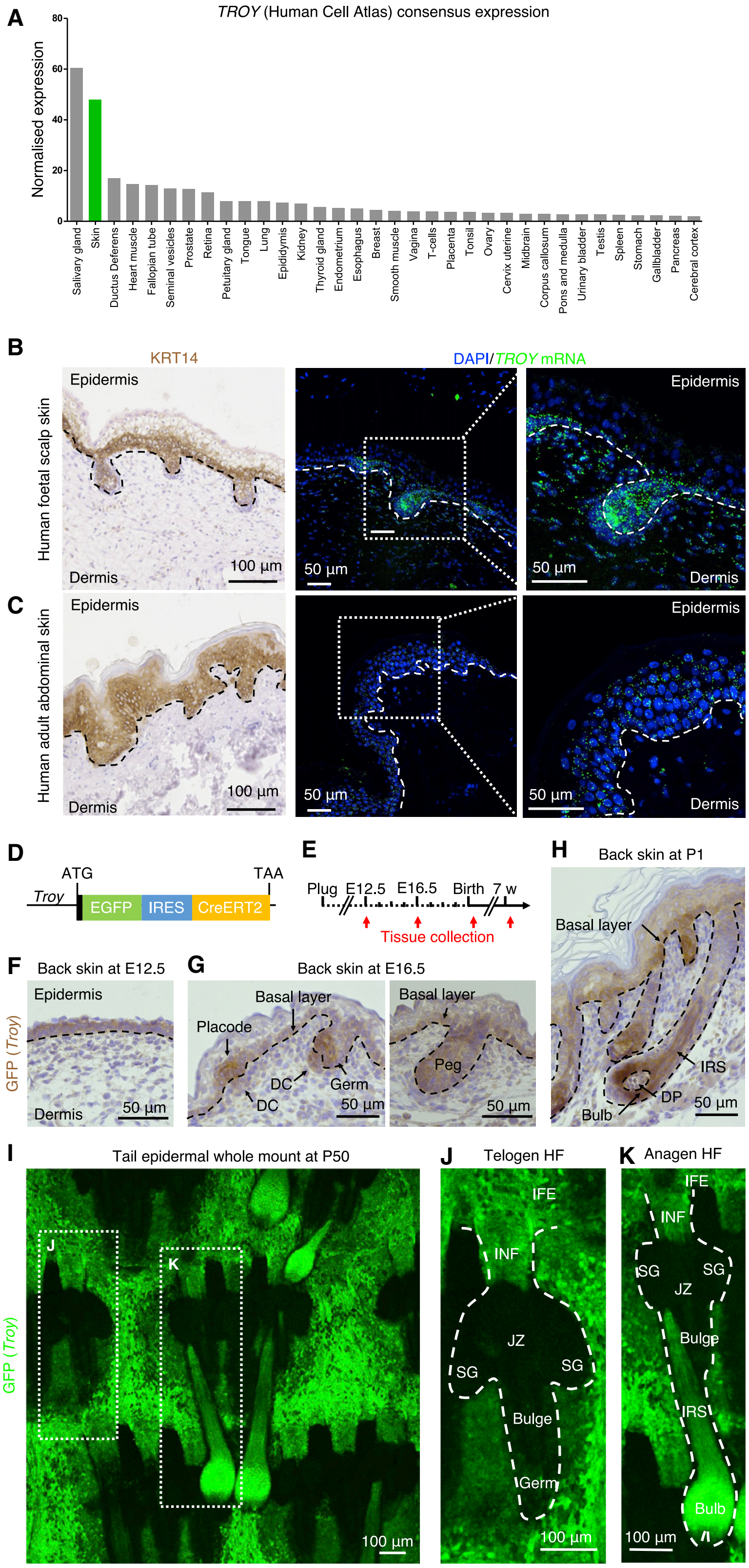

Figure 1. *TROY* expression in human and mouse embryonic, fetal, and adult skin

(A) Normalized Human Protein Atlas consensus *TROY* mRNA expression.

(B and C) Paraffin sections of human fetal scalp skin (B) and human adult abdominal skin (C) stained for K14 and RNAscope staining of human *TROY* transcripts.

(D) Schematic representation of the *Troy*-EGFP knockin mouse model.

(E) Experimental timeline of tissue collection.

(F–H) Paraffin sections of E12.5 (F), E16.5 (G), and P1 (H) back skin stained for EGFP. DC, dermal condensate; DP, dermal papilla; IRS, inner root sheath.

(I–K) Tail epidermal whole mounts (P50) of *Troy*-EGFP mice stained for EGFP. Outlined are a telogen hair follicle (J) and anagen hair follicle (K). IFE, interfollicular epidermis; INF, infundibulum; JZ, junctional zone; SG, sebaceous gland.

See also Figures S1 and S2.

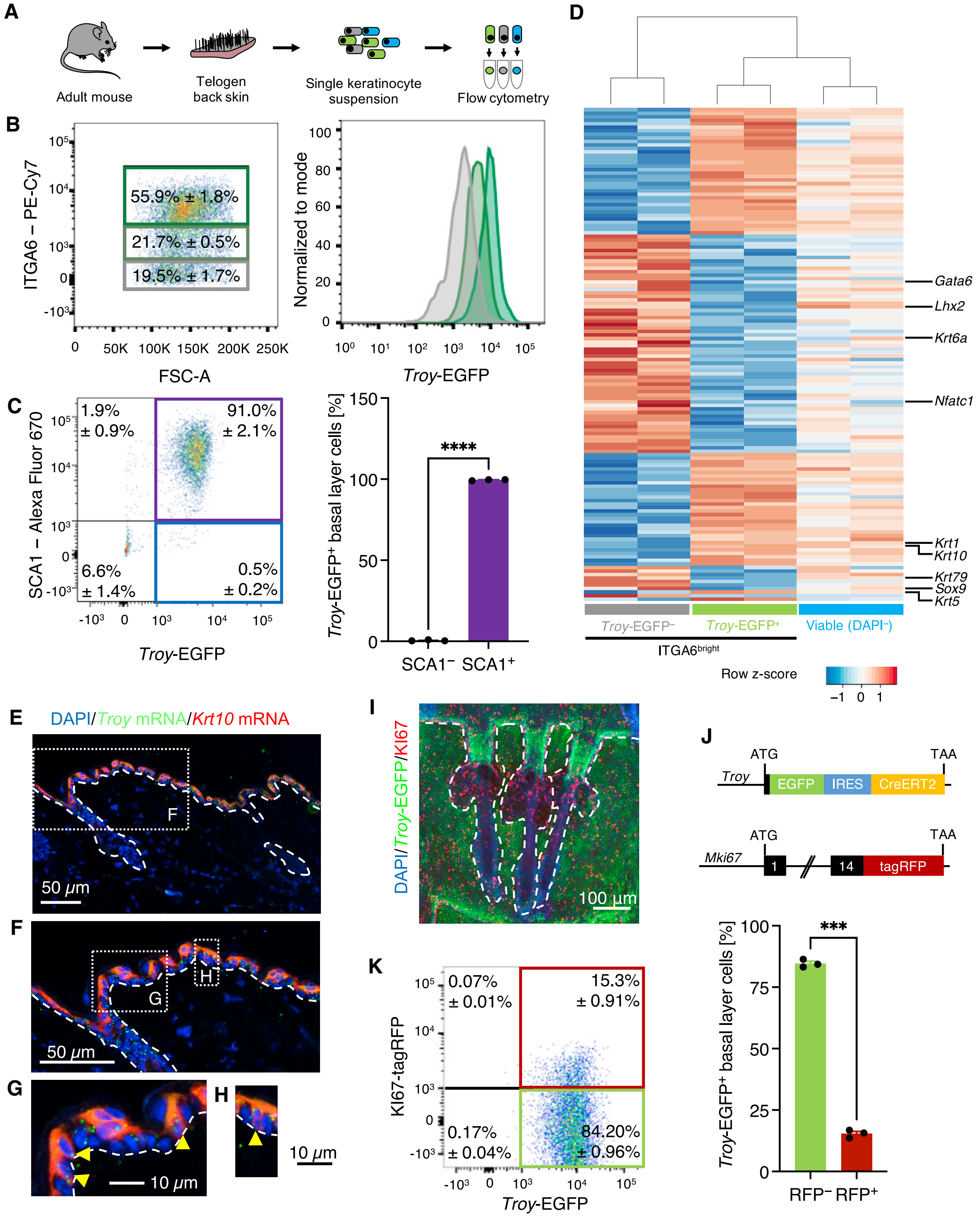

Figure 2. Characterization of *Troy*-expressing cells in adult murine telogen skin

(A) Experimental setup.

(B and C) Representative flow cytometry scatterplots of viable cells isolated from *Troy*-EGFP knockin mice stained for ITGA6 (CD49f) (B) and SCA1 (C). Histogram of *Troy*-EGFP expression normalized to mode (B). Dark green, ITGA6^{bright} cells; green, ITGA6^{mid} cells; gray, ITGA6^{dim} cells (B). Column chart indicating the percentage of SCA1^{-} and SCA1^{+} cells within the *Troy*-EGFP^{+} population (C). The data are presented as mean ± SEM (n = 3 mice). Student's t test, ****$p < 0.0001$.

(D) Heatmap of the bulk mRNA sequencing showing the differentially expressed genes comparing *Troy*-EGFP^{+} and *Troy*-EGFP^{-} ITGA6^{bright} cells.

(legend continued on next page)

Troy-expressing basal layer cells are SCA1$^+$ and highly proliferative

To better characterize the properties of *Troy*-expressing cells, we collected the telogen skin of adult *Troy*$^{EGFP\text{-}IRES\text{-}CreERT2}$ mice, isolated epidermal cells, and performed flow cytometry (Figure 2A). We separated the epidermal cells into three fractions based on their expression of ITGA6 (CD49f) and assessed EGFP expression (Figures 2B and S3A). Basal layer cells, marked by high levels of ITGA6, showed the highest expression intensity of EGFP (Figure 2B). With decreasing expression levels of ITGA6, the levels of EGFP expression decreased too, suggesting that TROY is highly associated with epidermal basal layer cells. In line with our observations above, we found that virtually all *Troy*-EGFP$^+$ basal epidermal cells (>99%), were SCA1$^+$, confirming their IFE/INF identity (Figure 2C). This was further supported by bulk messenger RNA (mRNA) sequencing performed on ITGA6^{bright} basal epidermal cells sorted based on EGFP expression (Figures 2D and S3B–S3E). We found 140 differentially expressed genes (adjusted $p < 0.05$) when comparing *Troy*-EGFP$^+$ and *Troy*-EGFP$^-$ ITGA6^{bright} basal layer cells (Figures 2D and S3B–S2E; Table S1). *Troy*-EGFP$^+$ basal epidermal cells were enriched for genes associated with the IFE lineage, such as *Krt1* and *Krt10* (Figures 2D and S3D). Among the significantly downregulated genes in the *Troy*-EGFP$^+$ cell population were the HF bulge markers *Krt6a*, *Lhx2*, *Nfatc1*, and *Sox9*, as well as the hair canal marker *Krt79* and the sebaceous duct marker *Gata6* (Figures 2D and S3D). Expression of the differentiation marker KRT10 in the epidermal basal layer has previously been observed (Braun et al., 2003). To validate our *in silico* data, we performed RNAscope for *Troy* and *Krt10* on sections of back skin of wild-type mice (Figures 2E–2H). Indeed, several epidermal basal cells showed co-expression of both *Troy* and *Krt10* confirming our RNA sequencing data (Figures 2G and 2H). Since actively cycling cells can be found throughout the epidermal basal layer (Figure S3F), we next investigated the proliferative status of *Troy*-EGFP$^+$ cells. We first stained tail epidermal whole mounts of adult *Troy*$^{EGFP\text{-}IRES\text{-}CreERT2}$ mice for EGFP and KI67, a marker of actively cycling cells (Hutchins et al., 2010). We found co-expression of EGFP and KI67 in basal layer cells of IFE and INF (Figure 2I). To quantify the overlap of EGFP and KI67, we generated mice harboring both *Troy*$^{EGFP\text{-}IRES\text{-}CreERT2}$ and *Mki67*^{tagRFP} expression cassettes (Basak et al., 2018) (Figure 2J) and isolated epidermal cells from adult telogen back skin. Using flow cytometry, we determined that virtually all KI67-tagRFP$^+$ IFE/INF basal (SCA1$^+$ ITGA6^{bright}) epidermal cells were *Troy*-EGFP$^+$ (>99%; Figure S3G) and about 15% of all *Troy*-EGFP$^+$ basal epidermal cells were positive for KI67-tagRFP (Figure 2K). This is in line with previous studies showing that proliferation in the IFE basal layer ranges from about 9% (via DNA content measurement) and 17.7% (via bromodeoxyuridine incorporation) (Cianfarani et al., 2011; Mascré et al., 2012). Taken together, these data indicate that *Troy* marks IFE/INF basal cells and enriches for proliferative cells.

Troy-expressing basal layer cells are highly clonogenic in organoid cultures

To assess the clonogenic potential of *Troy*-EGFP$^+$ cells, we performed organoid-forming efficiency (OFE) assays (Figure 3A). In this functional assay, organoids grown from a single cell serve as a proxy for stem cell capacity (Boonekamp et al., 2019). We purified different cell populations using flow cytometry (Figure 3B), plated single cells, and assessed organoid formation 7 days later (Figure 3C). Organoid cultures generated from *Troy*-EGFP$^+$ cells had a significantly higher cell viability and contained more and larger organoids in comparison with cultures derived from *Troy*-EGFP$^-$ cells (Figures 3C–3F). In a second step, we analyzed organoid formation from four different cell populations, which were sorted based on their expression of *Troy*-EGFP and the basal layer marker ITGA6 (Figure 3B). Firstly, ITGA6^{bright} basal layer cells positive for *Troy*-EGFP showed a higher OFE than those negative for the reporter (Figures 3C–3F). Secondly, organoid cultures generated from ITGA6^{dim} suprabasal cells had a lower cell viability and contained fewer organoids—irrespective of their level of *Troy*-EGFP expression—than those of *Troy*-EGFP$^+$ ITGA6^{bright} cells (Figures 3C–3F). Thirdly, overall cell viability was comparable between organoid cultures of *Troy*-EGFP$^-$ ITGA6^{bright} basal cells and those derived from *Troy*-EGFP$^+$ ITGA6^{dim} suprabasal cells (Figure 3D). However, *Troy*-EGFP$^-$ ITGA6^{bright} basal cells formed more organoids than *Troy*-EGFP$^+$ ITGA6^{dim} cells (Figure 3E), while the organoids generated from *Troy*-EGFP$^+$ ITGA6^{dim} suprabasal cells were significantly larger than organoid derived from either *Troy*-EGFP$^-$ ITGA6^{bright} cells or *Troy*-EGFP$^-$ ITGA6^{dim} cells

(E–H) Representative image of paraffin sections of telogen back skin stained RNAscope probes against *Troy/Tnfrsf19* and *Krt10*. Yellow arrows in (G) and (H) indicate basal layer cells positive for *Krt10* mRNA and *Troy* mRNA.
(I) Tail epidermal whole mount of *Troy*-EGFP mice stained for EGFP and KI67.
(J) Schematic representation of the genetic constructs.
(K) Representative flow cytometry scatterplot of viable SCA1$^+$ ITGA6$^+$ cells assessed for expression of *Troy*-EGFP and KI67-tagRFP. Column chart indicating the percentage of KI67-tagRFP$^-$ and KI67-tagRFP$^+$ cells within the *Troy*-EGFP$^+$ population. The data are presented as mean ± SEM (n = 3 mice). Student's t test, ***p < 0.001.
See also Figure S3 and Table S1.

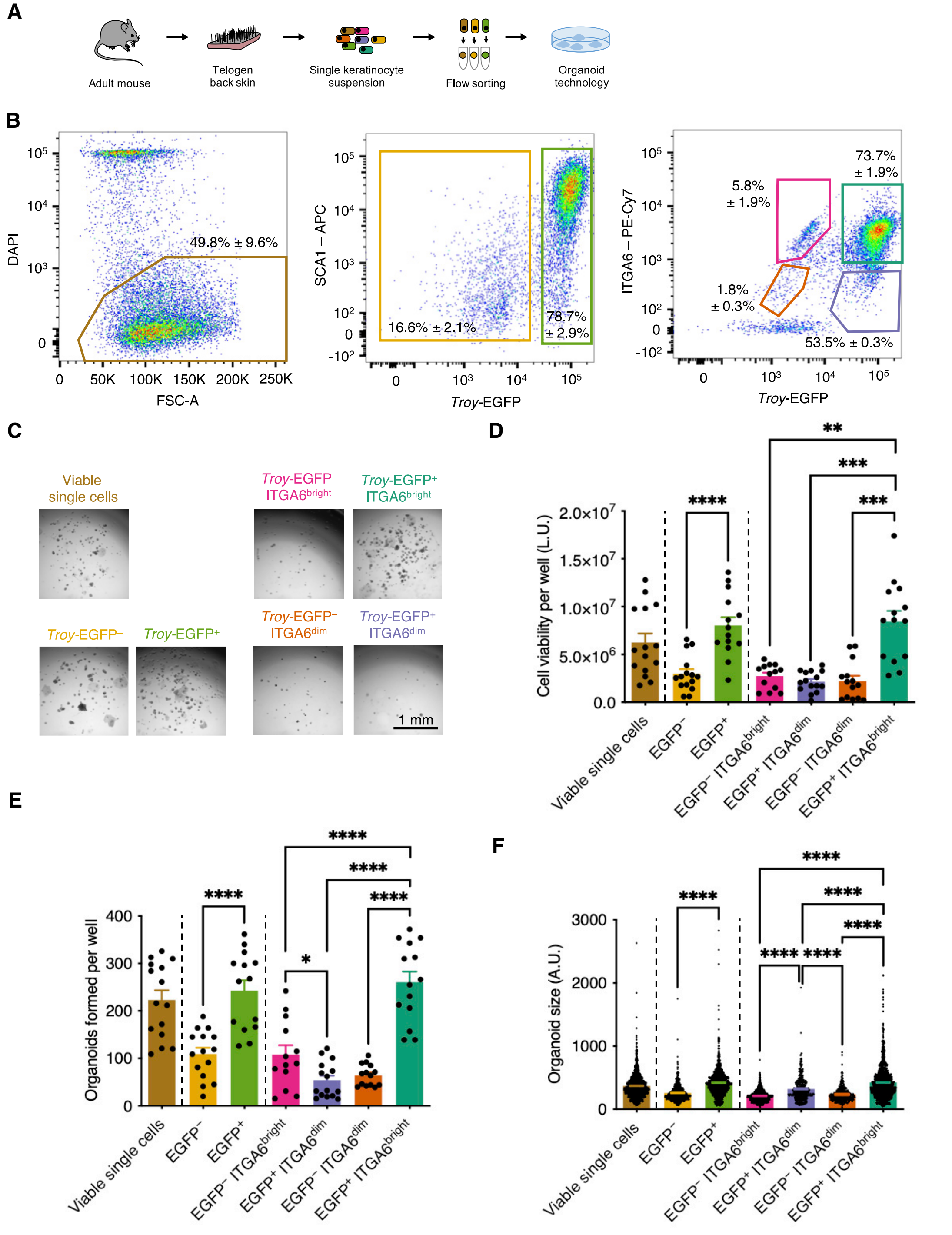

Figure 3. Characterization of *Troy*-expressing cells using organoid technology

(A) Experimental setup.

(B) Representative flow cytometry scatterplots of viable cells isolated from *Troy*-EGFP knockin mice stained for ITGA6 and SCA1 (n = 4).

(C) Representative bright-field images of sorted cell populations grown as organoids for 7 days.

(D–F) Quantification of the cell viability per well (D), number of organoids formed per well (E), and size of organoids formed (F) after 7 days of culture. The data are presented as mean ± SEM (two to four replicates/wells per mouse, n = 4 mice). Student's t test, $**p < 0.01$, $***p < 0.001$, $****p < 0.0001$. A.U., arbitrary units; L.U., light units.

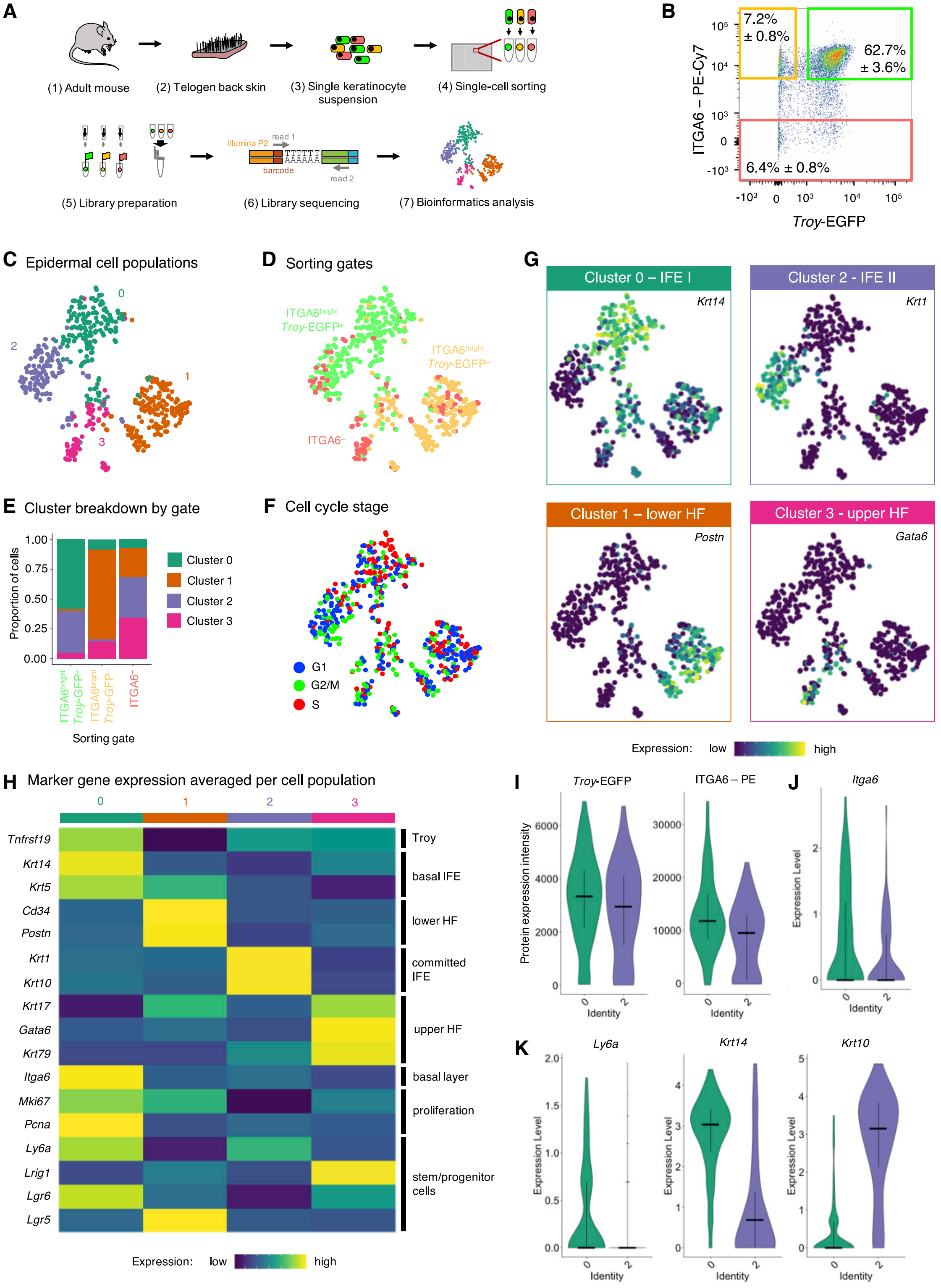

Figure 4. Single-cell transcriptomics of *Troy*-expressing cells and other epidermal cells

(A) Experimental setup.

(B) Representative flow cytometry scatterplots of viable cells isolated from *Troy*-EGFP knockin mice stained for ITGA6 (n = 4). Sorting gates are indicated in colors: ITGA6^{-} cells (red), ITGA6^{bright}*Troy*-EGFP^{-} cells (yellow), and ITGA6^{bright}*Troy*-EGFP^{+} cells (green).

(legend continued on next page)

(Figure 3F). Taken together, these data suggest that *Troy*-EGFP$^+$ basal layer cells are highly clonogenic. In addition, *Troy*-EGFP$^+$ differentiating cells in the suprabasal layer expectedly had a lower capacity to form organoids. However, some of these suprabasal *Troy*-EGFP$^+$ cells appeared plastic and responsive to stemness-promoting growth factors, as the capacity to form larger organoids was increased in comparison with both *Troy*-EGFP$^-$ basal and suprabasal cells (Figure 3F). This is line with previous observations showing that some suprabasal cells reacquire stem cell capacity under certain conditions (Donati et al., 2017; Kretzschmar et al., 2014).

Single-cell transcriptomics reveals that *Troy* marks two distinct populations of epidermal basal layer cells

To characterize the *Troy*-EGFP$^+$ cells in more depth and gain insights into possible heterogeneity, we sorted cells into 384-well plates and performed single-cell mRNA sequencing (Figure 4A). We separated the cells based on expression of *Troy*-EGFP and ITGA6 (Figure 4B) and sorted three populations: (1) (ITGA6$^-$) non-basal layer cells, (2) *Troy*-EGFP$^-$ (ITGA6^{bright}) basal layer cells, and (3) *Troy*-EGFP$^+$ (ITGA6^{bright}) basal layer cells and recorded flow cytometry parameters (Baron et al., 2019). Subsequently, samples were processed for mRNA sequencing using the SORT-seq method (Muraro et al., 2016) and data were analyzed using Seurat (Butler et al., 2018). After quality control and filtering of necrotic cells and non-epidermal cells (Figures S4A–S4E), remaining epidermal cells separated into four different clusters (Figures 4C–4E). Actively cycling cells were found in all clusters, with cluster 0 showing enrichment for cells in the S phase of the cell cycle (Figure 4F). In their comprehensive single-cell transcriptomics dataset of epidermal cells, Joost et al. (2016) defined different main populations of epidermal cells based on their markers. Based on these markers, we identified the four different populations (Figures 4G and 4H): We found that ITGA6$^-$ cells contributed mainly to cluster 3, which—based on their high expression of *Gata6*—contained upper HF cells. ITGA6^{bright} *Troy*-EGFP$^-$ basal layer cells were enriched in cluster 1 representing lower HF cells marked by *Postn*. ITGA6^{bright} *Troy*-EGFP$^+$ basal layer cells contributed to clusters 0 and 2, marked by *Krt14* and *Krt1*, respectively. As ITGA6^{bright}*Troy*-EGFP$^+$ cells clustered into two different epidermal cell populations, we aimed to gain more insights into these two cell clusters. We therefore projected the flow cytometry data recorded during cell sorting (index sorting) onto the transcriptomic data of each cell. Clusters 0 and 2 showed similar intensities for EGFP and ITGA6 (Figure 4I). *Itga6* and *Ly6a* (encoding the IFE basal cell marker SCA1) gene expression was not altered between both clusters (Figures 4J and K). However, gene expression of *Krt14* were strongly reduced in cluster 2, while *Krt10* expression was robustly upregulated (Figure 4K). Interestingly, our data therefore imply the presence of two distinct IFE basal cell populations marked by ITGA6^{bright} and *Troy*-EGFP$^+$: one population (IFE I) showing clear features of undifferentiated IFE basal cells (*Krt14*$^+$/*Ly6a*$^+$ and enriched for cells in S phase) and the other one (IFE II) appearing as (*Krt10*$^+$) committed IFE basal cells, in line with our observations above (Figures 2E–2H).

Troy-expressing cells contribute to long-term IFE and INF maintenance

To assess the long-term fate of *Troy*-expressing cells during homeostasis, we crossed *Troy*$^{EGFP\text{-}IRES\text{-}CreERT2}$ mice with *Rosa26-loxP*-STOP-*loxP*-tdTomato (LSL-tdTomato) mice to allow for genetic lineage-tracing experiments (Kretzschmar and Watt, 2012) (Figure 5A). Double mutant mice received tamoxifen injections at the age of 7–9 weeks when back skin HFs are in telogen (Müller-Röver et al., 2001). Skin tissue of back, tail, ear, and paws was collected 1 day, 7 days, 1 month, and 6 months after tamoxifen injections (Figure 5B). We stained epidermal tail whole mounts for tdTomato and DAPI and scored the different epidermal compartments with tdTomato$^+$ clones (Figure 5C). At 1 day and 1 week post tamoxifen, robust initial tdTomato labeling was found in the hair germ, IFE, and INF (Figure 5D). However, tracings for up to 6 months demonstrated that tdTomato$^+$ clones primarily remained long term in IFE and INF only (Figure 5D). Next, we assessed whether *Troy*-expressing cells contributed to IFE homeostasis through generation of differentiated suprabasal progeny. We performed co-stainings for KRT14 and EGFP on sections of skin tissue collected from the tamoxifen-treated mice (Figure S5A). At 1-day post tamoxifen, an average of 2 KRT14$^+$ IFE basal cells were tdTomato$^+$, while no tdTomato labeling was found in the KRT14$^-$ suprabasal layer (Figures S5A–S5C). After 1 week

(C and D) *t*-SNE plot indicating the four different clusters identified within the epidermal cell populations (C) and the sorting gates (D).
(E) Stacked column chart indicating cluster breakdown by gate.
(F) Cell-cycle analysis.
(G) Key marker gene expression per cluster.
(H) Heatmap showing marker gene expression averaged per cell population for all identified epidermal clusters.
(I–K) Violin plots comparing the EGFP and ITGA6 protein expression intensity (I), *Itga6* expression levels (J), and the expression levels of *Ly6a*, *Krt14*, and *Krt10* (K) between the cells in clusters 0 and 2. Horizontal bars indicate the median and cross bars indicate the quartiles. See also Figure S4.

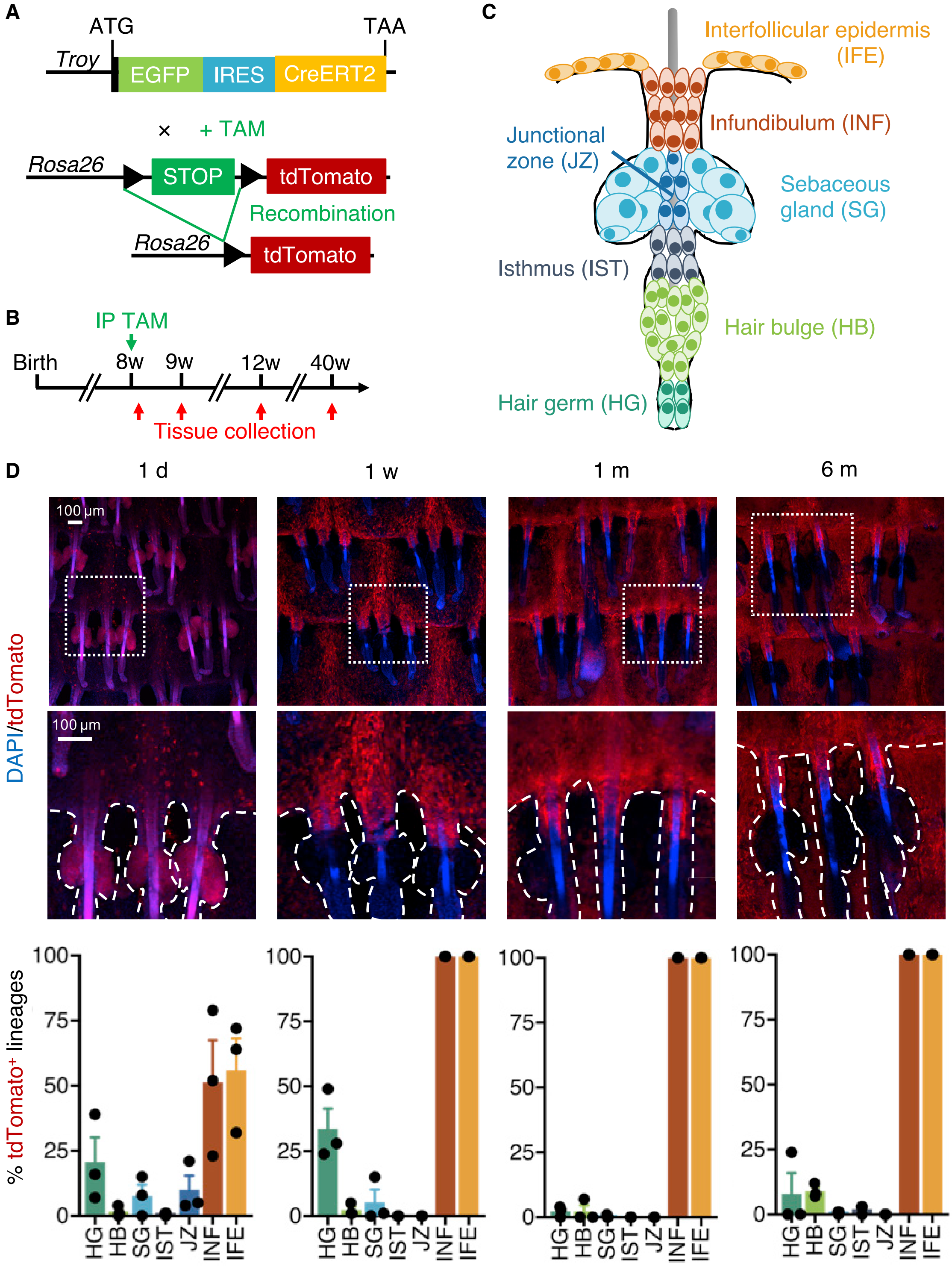

Figure 5. Genetic lineage tracing of *Troy*-expressing cells

(A) Schematic representation of the genetic constructs.

(B) Experimental timeline.

(C) Schematic overview of a tail whole-mount hair follicle with indicated epidermal compartments.

(legend continued on next page)

of lineage tracing, about 40 basal cells per field were tdTomato$^+$, with additional tdTomato labeling of approximately 10 suprabasal keratinocytes per field (Figures S5A–S5C). Suprabasal tdTomato labeling increased further to 20 cells per field at 1 month post injection, while basal tdTomato labeling did not change significantly (Figures S5A–S5C). After 6 months of tracing, tdTomato labeling of both basal and suprabasal cells remained stable with no significant change compared with the labeling detected at 1 month post injection (Figures S5B and S5C). Similar long-term tracings were found in paw epidermis devoid of HFs, confirming that an epidermal stem cell population independent of the HF governs cellular input into the suprabasal layers of the cornifying epidermis (Figure S5D). In summary, these data demonstrate that *Troy*-expressing cells generate differentiating progeny long term, suggesting stem cell capacity of TROY$^+$ cells *in vivo*.

Characterization of the progeny of *Troy*-expressing cells using single-cell transcriptomics

To further characterize the progeny of *Troy*-expressing cells at the transcriptional level, we first—for increased sensitivity to tamoxifen-induced Cre recombination—generated double homozygous *Troy*$^{EGFP\text{-}IRES\text{-}CreERT2}$ × LSL-tdTomato knockin mice and then performed lineage tracing for 7 days (Figure 6A). We then prepared single-cell suspensions from adult telogen back skin and sorted the different cell populations based on their expression of ITGA6 (ITGA6$^-$, ITGA6^{dim}, and ITGA6^{bright}), *Troy*-EGFP, as well as tdTomato (Figure 6B), and performed single-cell mRNA sequencing. Almost all *Troy*-EGFP$^+$ cells also showed tdTomato$^+$ expression, suggesting a labeling efficiency at saturating levels (>99%; Figure 6B). After quality control and filtering (Figure S6), we used the Seurat algorithm to project the newly generated single-cell dataset onto the initially analyzed *Troy*-EGFP dataset (Figures 4A–4K) based on matching cellular identities to the predefined clusters from our *Troy*-EGFP dataset (Figures 6C and 6D). Based on marker expression, the four clusters of undifferentiated *Krt14*$^+$ IFE basal cells (IFE I), committed *Krt10*$^+$ IFE basal cells (IFE II), *Postn*$^+$ lower HF cells, and *Gata6*$^+$ upper HF cells could be identified (Figure 6D). The majority of (tdTomato$^+$) *Troy*-EGFP$^+$ ITGA6^{bright} cells identified as undifferentiated *Krt14*$^+$ IFE basal cells, while almost all (tdTomato$^+$) *Troy*-EGFP$^+$ ITGA6^{dim} cells were assigned to the cluster of *Gata6*$^+$ upper HF cells (Figures 6D–6F). *Troy*-EGFP$^-$ tdTomato$^-$ cells were enriched in the cluster of *Postn*$^+$ cells (Figures 6D–6F), suggesting that they originate from the lower HF.

These results are in line with the OFE assays (Figure 3), as both experiments demonstrated that *Troy*-EGFP$^+$ ITGA6^{bright} cells have stem cell capacity, while tdTomato$^+$ *Troy*-EGFP$^-$ ITGA6^{dim} cells appear to be differentiating cells. To confirm these observations, we stained tail epidermal whole mounts collected from the mice sampled for these experiments for tdTomato and ITGA6 (Figure 6G). TdTomato$^+$ cells were found in both the ITGA6^{bright} basal layer and the ITGA6^{dim} suprabasal layers of IFE and INF (Figure 6G), confirming the single-cell data. As *Troy*-EGFP$^+$ cells were enriched in IFE and INF in telogen skin, tdTomato$^+$ progeny of *Troy*-expressing cells was robustly identified as belonging to these lineages without significant contribution to other epidermal compartments (Figures 6D–6F), in line with the notion that epidermal stem cell niches are compartmentalized in adult homeostasis (Kretzschmar et al., 2016; Page et al., 2013). In conclusion, these data demonstrated the existence of a *Troy*-expressing cell population in the IFE/INF basal layer with stem cell capacity that readily contributes differentiating daugther cells to the cornifying layers of the epidermis.

DISCUSSION

Here, we identify *Troy* as a marker gene of epidermal cells that govern IFE and INF homeostasis. In telogen skin, *Troy*$^+$ cells reside in the ITGA6^{bright} basal layer of IFE and INF. Genetic-tracing experiments demonstrate that progeny of *Troy*-expressing cells in these compartments of the so-called permanent portion of the epidermis contribute to cellular differentiation of cells and the cornified envelope long term, validating the stem cell capacity of *Troy*$^+$ cells *in vivo*. Furthermore, *Troy*$^+$ cells have a robust organoid-forming capacity, validating their stem cell potential *in vitro*.

We found human skin to be the tissue with the second highest expression levels of *TROY* among all human organs studied by the Human Protein Atlas. Using RNAscope technology, we also found that *TROY* transcripts were expressed by keratinocytes in the KRT14$^+$ IFE basal layer and the developing HFs in fetal human skin, as well as in the KRT14$^+$ IFE basal layer of adult human skin. Future studies may investigate the potential functional role of TROY$^+$ epidermal cells in human skin. In murine telogen skin, *Troy* expression was restricted to the IFE and INF, the two cornifying epidermal compartments. However, during anagen, cells of the proliferative hair bulb were highly

(D) Tail epidermal whole mounts following 1 day, 7 days, 1 month, or 6 months induction of lineage tracing stained for tdTomato (red) and counterstained with DAPI (blue). Column charts indicating quantification of lineage tracing over the time course. Twenty-five triplets containing hair follicles of different hair cycle stages were quantified for tdTomato clones (n = 2–3 mice). Data indicate mean ± SD. See also Figure S5.

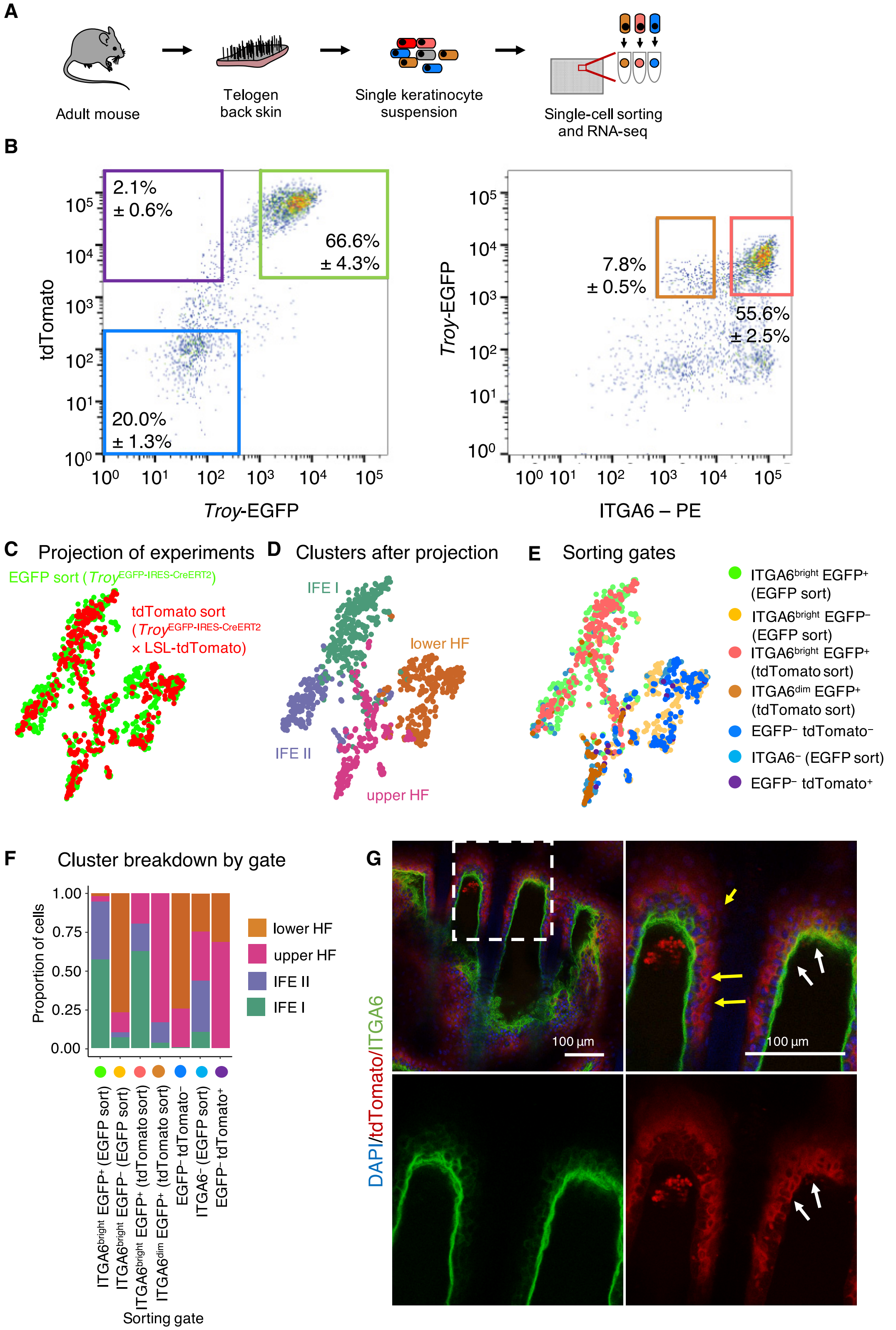

(legend on next page)

enriched for *Troy*, while low levels of *Troy* were also detected in the cells of the IRS that contribute to the keratinizing and hair shaft-producing layers of the HF. This suggests that the Wnt/β-catenin target gene *Troy* marks stem/progenitor cells collectively governing the cellular input into cornification and keratinization. A similar correlation was found in embryonic murine epidermis in line with previous studies demonstrating a functional (but redundant) role for TROY in HF morphogenesis (Kojima et al., 2000; Pispa et al., 2008).

Single-cell transcriptomics revealed that ITGA6^{bright} *Troy*-EGFP^{bright} IFE basal layer cells separate into two distinct cell populations. One population is defined by undifferentiated IFE basal cell markers genes, such as *Krt14*, *Krt5*, and *Ly6a* (IFE I), while the other population is enriched for transcripts of differentiation markers, such as *Krt1* and *Krt10* (IFE II), defining committed IFE basal cells. In line with our observations (Figures 2E–2H), a recent study found robust gene and protein expression of differentiation markers, such as KRT10, in the IFE basal layer (Cockburn et al., 2021). Using intravital imaging, the authors further demonstrated that almost all IFE basal cells (96%) expressing a KRT10 reporter exited the basal layer within 10 days of tracking. In addition, expression of differentiation markers has already been used to target committed cells in the epidermal basal layer by Mascré et al. (2012). In their paper, the authors demonstrate the existence of a population of committed epidermal basal layer cells that contribute to IFE homeostasis using a genetic lineage-tracing mouse model driven by terminal differentiation marker gene *Involucrin* (*Ivl*). Furthermore, the presence of committed epidermal cells in direct contact with the basement membrane through ITGA6—and therefore residing in the epidermal basal layer—is in line with the observations by Watt and colleagues showing that the basal layer of human epidermis is rather heterogeneous containing highly clonogenic cells marked by high levels of β1 integrin and cells with lower colony-forming efficiency and low β1 integrin expression (Jones et al., 1995; Jones and Watt, 1993; Tan et al., 2013).

Genetic lineage tracing confirmed that *Troy*-expressing cells in the IFE self-renew and produce differentiating progeny in the long term, independent of the HF, as demonstrated by tdTomato-labeling ranging from the basal layer to the cornified envelope of HF-free paw epidermis. These observations are in line with Lim et al. (2013), showing that stem cells located in the IFE act in a compartmentalized fashion separate from the influence of HFs in adult homeostasis. Interestingly, when an HF is attached to the IFE, such as in the epidermis of back, ear, and tail skin, *Troy* is expressed by both IFE and INF basal layer cells and long-term contribution to the cornified envelope can be found from both compartments. This finding is in line with Joost et al. (2016), showing that the basal cells of both epidermal compartments are transcriptionally rather similar.

Compartment-restricted activation of oncogenes, such as KRAS^{G12D}, or inactivation of tumor suppressor mutations, such as p53$^{-/-}$ in the IFE (and INF) as discussed previously (Blanpain, 2013), has been challenging due to lack of specific CreER mouse models (Lapouge et al., 2011). Although efforts were made to assess the ability of the IFE to form squamous cell carcinomas (SCCs) using *IVL*-CreERT2 transgenic mice to simultaneously ablate p53 and induce oncogenic KRAS, the results remained inconclusive, as *IVL*-CreERT2 targets IFE basal cells dedicated to differentiation and not long-term maintained stem cells (Mascré et al., 2012). Therefore, as *Troy* expression marks stem cells in the basal layer of the IFE and INF in telogen skin, the *Troy*-driven CreERT2 mouse model allows approaches like the testing of the origin of specific skin tumors, such as SCCs. Induction of CreER elsewhere in the mouse body can be avoided by local application of tamoxifen.

In addition, this mouse model could be applied in the study of basal cell dynamics in more depth during homeostasis and upon wounding. Epidermal stem cell populations have been shown to be highly plastic upon wounding, where stem cells from non-IFE or INF compartments replenish lost IFE stem cells and contribute to wound healing as well as long-term homeostasis after regeneration (Dekoninck and Blanpain, 2019). Despite the plastic behavior of stem cells upon wounding, it would be relevant to determine the proportion of IFE and INF stem cells contributing to wound healing and observe

Figure 6. Characterization of progeny of *Troy*-expressing cells
(A) Schematic overview of experimental outline.
(B) Representative flow cytometry scatterplot of viable cells isolated from *Troy*$^{EGFP\text{-}IRES\text{-}CreERT2}$ × LSL-tdTomato mice 7 days post induction of lineage tracing stained against ITGA6. Sorting gates are indicated in colors (for color coding, see (G)).
(C–E) *t*-SNE map of single-cell sequencing data generated from 7 day induced mice (n = 2 mice) projected onto the dataset generated for Figure 4 C, with assigned clusters (D) and all sorting gates indicated (E).
(F) Stacked column chart indicating cluster breakdown by gate.
(G) Tail epidermal whole mount of a *Troy*$^{EGFP\text{-}IRES\text{-}CreERT2}$ × LSL-tdTomato mouse 7 days post tamoxifen injection stained for tdTomato and ITGA6. Zoom-ins and arrows indicate suprabasal tracing (yellow arrows) or basal tracing (white arrows).
See also Figure S6.

whether these dedicated IFE and INF stem cells can contribute to long-term tracing in newly formed HFs. Wounding studies in *Lgr6*$^{EGFP-IRES-CreERT2}$ mice have shown the contribution of *Lgr6*-expressing cells to long-term maintenance of HFs; however, *Lgr6* is not exclusively expressed in the basal layer of the IFE and INF, but also in the HF junctional zone and SG (Füllgrabe et al., 2015; Kretzschmar et al, 2014, 2016; Page et al., 2013; Snippert et al., 2010). The *Troy* knockin mouse model therefore would allow a more refined tracing of IFE/INF basal cells in this context.

In conclusion, our study identifies epidermal basal cells in the IFE and INF with stem cell capacity marked by *Troy*. In contrast to other IFE-associated stem cell markers, such as *Axin2* and *Lgr6* (Füllgrabe et al., 2015; Lim et al., 2013), *Troy* is confined to the basal layer of the cornifying compartment and not robustly expressed in the SG or lower HF in telogen skin. With the characterization of *Troy*-expressing cells, opportunities arise to study disease and regenerative capacity specific to the basal layer of IFE and INF. Our data provide further evidence for cellular heterogeneity in the epidermal basal layer and show that *Troy* marks a lineage of basal cells including stem cells and those already committed to differentiation.

EXPERIMENTAL PROCEDURES

Human tissue

The use of human fetal scalp skin (16 weeks of gestation) was approved by the medial ethical committees of the LUMC (P08.087) and patient written informed consent was obtained beforehand. Human adult abdominal skin samples were obtained as discarded material after cosmetic surgery from anonymous donors who gave prior written informed consent for the use of material in research.

Mouse lines

Mice were housed in the animal facility of the Hubrecht Institute and experiments were carried out under a Dutch government project license granted to Prof. Hans Clevers. The following experimental protocols were approved by the animal welfare committee of Utrecht University. Both male and female mice were used, except for experiments in Figure 6, where only male mice were used. Littermates were used as no-tamoxifen controls. Generation of *Mki67*^{tagRFP} expression cassettes (Basak et al., 2018), *Troy*$^{EGFP-IRES-CreERT2}$ (Stange et al., 2013) and LSL-tdTomato mice (Madisen et al., 2010) was described elsewhere. Details are provided in the supplemental experimental procedures.

Troy expression in human tissue

TROY consensus expression data were downloaded from v#19.proteinatlas.org (Human Protein Atlas) (Uhlén et al., 2015). Only tissues with normalized expression >2 were included.

Murine epidermal keratinocyte isolation and flow cytometric purification

Isolation of keratinocytes from back skin was performed as described previously (Jensen et al., 2010). Isolated single cells were resuspended in FACS buffer (2 mM EDTA and 2% FBS in PBSO) at a density of 1 × 10^6 cells per mL. Cells were stained on ice for 1 h with the following antibodies: rat anti-human/mouse ITGA6 (CD49f)-PE/Cy7 (555736, BD Biosciences, or 313621, BioLegend), rat anti-mouse Ly6A (SCA-1)-APC (17-5981-81, eBioscience) and rat anti-mouse CD34 (560230, BD Biosciences). Immediately before flow sorting, 4′,6-diamidino-2-phenylindole (DAPI) was added. Cells were either collected for bulk mRNA sequencing in TRIzol (Invitrogen) or for single-cell sequencing and sorted in 384-well format. All samples were stored at −80°C.

Organoid experiments

For organoid culture experiments, cells were first isolated from back skin and sorted by flow cytometry based on several markers. Culture of murine epidermal organoids was performed as described previously (Boonekamp et al., 2019). Details are provided in the supplemental experimental procedures.

RNA sequencing

RNA sequencing was performed using the CEL-Seq2 method (Hashimshony et al., 2016), as detailed in the supplemental experimental procedures.

Histology

For paraffin sections, skin from the back, tails, ears, and paws was collected from *Troy*$^{EGFP-IRES-CreERT2}$ mice and *Troy*$^{EGFP-IRES-CreERT2}$ × LSL-TdTomato lineage-traced mice. Tissue was immediately fixed overnight in formalin at room temperature. Paw tissue was decalcified for at least 2 weeks in 10% EDTA after fixation. Procedures for paraffin embedding and stainings, tail whole-mount stainings, and RNAscope assays are described in the supplemental experimental procedures.

Imaging

Tail whole-mount images and paraffin immunofluorescent images were acquired on a confocal microscope (Leica SP8X and SP8). Paraffin sections stained using immunohistochemistry were imaged on a Leica DM4000 microscope.

Bioinformatics analysis

Sequencing, mapping to the mouse reference genome, and transcript counting of the DNA libraries were performed as described elsewhere (Kretzschmar et al., 2018). Bulk mRNA sequencing samples were analyzed using the DESeq2 package (Love et al., 2014). Single-cell mRNA sequencing libraries were analyzed using the Seurat v.3 package (Butler et al., 2018). All bioinformatics analyses were performed using R v.3.4.0 (R Foundation, https://www.r-project.org) and RStudio v.1.0.143 (https://www.rstudio.com). Details are provided in the supplemental experimental procedures.

Data and code availability

The accession number for the sequencing data reported in this paper is Gene Expression Omnibus (GEO): GSE165379.

SUPPLEMENTAL INFORMATION

Supplemental information can be found online at https://doi.org/10.1016/j.stemcr.2021.07.007.

AUTHOR CONTRIBUTIONS

K.K., K.E.B., and H.C. conceived the project, designed experiments, and interpreted results. K.K. and K.E.B. performed animal experiments. K.K., K.E.B., M.B., P.A., and M.K. performed all histology, imaging, and cell culture experiments. K.K. and K.E.B. performed sequencing experiments. K.K. performed bioinformatics analysis. S.M.C.d.S.L. and B.G. obtained human skin tissue. K.K. and H.C. acquired funding. K.K., K.E.B., and H.C. wrote the manuscript with input from all other authors.

CONFLICTS OF INTEREST

The authors declare no competing interests. H.C. is the inventor on several patents related to organoid technology. He is cofounder of Surrozen, D1Med, and Xilis; board of directors-member of Roche/Genentech and SAB member of Volastra, Decibel, and Merus. His full disclosure is given at https://www.uu.nl/staff/JCClevers/.

ACKNOWLEDGMENTS

We thank Onur Basak, Johan van Es and Maaike van den Born for experimental assistance; Stefan van der Elst and Reinier van den Linden for help with flow cytometry; the Utrecht Sequencing Facility (USEQ) for sequencing; Anna Alemany Arias for help with bioinformatics analysis and Anko de Graaff and the Hubrecht Imaging Centre (HIC) for assistance with microscopy. We thank the Gynaikon Clinic in Rotterdam for the efforts in providing the fetal material. This work was supported by a Sinergia grant from the Swiss National Science Foundation (SNSF/CRSII3 160738-1, to H.C.) and by a German Cancer Aid grant (via MSNZ Würzburg, to K.K.). K.K. was recipient of a VENI grant from the Netherlands Organisation for Scientific Research (NWO-ZonMW, 016.166.140) and was a long-term fellow of the Human Frontier Science Program Organization (HFSPO, LT771/2015).

Received: April 15, 2020
Revised: July 6, 2021
Accepted: July 7, 2021
Published: August 5, 2021

REFERENCES

Barker, N., van Es, J.H., Kuipers, J., Kujala, P., van den Born, M., Cozijnsen, M., Haegebarth, A., Korving, J., Begthel, H., Peters, P.J., et al. (2007). Identification of stem cells in small intestine and colon by marker gene Lgr5. Nature *449*, 1003–1007.

Barker, N., Huch, M., Kujala, P., van de Wetering, M., Snippert, H.J., van Es, J.H., Sato, T., Stange, D.E., Begthel, H., van den Born, M., et al. (2010). Lgr5(+ve) stem cells drive self-renewal in the stomach and build long-lived gastric units in vitro. Cell Stem Cell *6*, 25–36.

Baron, C.S., Barve, A., Muraro, M.J., van der Linden, R., Dharmadhikari, G., Lyubimova, A., de Koning, E.J.P., and van Oudenaarden, A. (2019). Cell type purification by single-cell transcriptome-trained sorting. Cell *179*, 527–542.

Basak, O., Krieger, T.G., Muraro, M.J., Wiebrands, K., Stange, D.E., Frias-aldeguer, J., Rivron, N.C., van de Wetering, M., van Es, J.H., van Oudenaarden, A., et al. (2018). Troy+ brain stem cells cycle through quiescence and regulate their number by sensing niche occupancy. Proc. Natl. Acad. Sci. *115*, E610–E619.

Blanpain, C. (2013). Tracing the cellular origin of cancer. Nat. Cell Biol. *15*, 126–134.

Boonekamp, K.E., Kretzschmar, K., Wiener, D.J., Asra, P., Derakhshan, S., Puschof, J., Lopez-Iglesias, C., Peters, P.J., Basak, O., and Clevers, H. (2019). Long-term expansion and differentiation of adult murine epidermal stem cells in 3D organoid cultures. Proc. Natl. Acad. Sci. *116*, 14630–14638.

Braun, K.M., Niemann, C., Jensen, U.B., Sundberg, J.P., Silva-Vargas, V., and Watt, F.M. (2003). Manipulation of stem cell proliferation and lineage commitment: visualisation of label-retaining cells in wholemounts of mouse epidermis. Development *130*, 5241–5255.

Brownell, I., Guevara, E., Bai, C.B., Loomis, C.A., and Joyner, A.L. (2011). Article nerve-derived sonic hedgehog defines a niche for hair follicle stem cells capable of becoming epidermal stem cells. Stem Cell *8*, 552–565.

Butler, A., Hoffman, P., Smibert, P., Papalexi, E., and Satija, R. (2018). Integrating single-cell transcriptomic data across different conditions, technologies, and species. Nat. Biotechnol. *36*, 411–420.

Cianfarani, F., Bernardini, S., De Luca, N., Dellambra, E., Tatangelo, L., Tiveron, C., Niessen, C.M., Zambruno, G., Castiglia, D., and Odorisio, T. (2011). Impaired keratinocyte proliferative and clonogenic potential in transgenic mice overexpressing 14-3-3σ in the epidermis. J. Invest. Dermatol. *131*, 1821–1829.

Cockburn, K., Annusver, K., Ganesan, S., Mesa, K.R., Kawaguchi, K., Kasper, M., and Greco, V. (2021). Gradual differentiation uncoupled from cell cycle exit generates heterogeneity in the epidermal stem cell layer. bioRxiv https://doi.org/10.1101/2021.01.07.425777.

Cotsarelis, G., Sun, T., and Lavker, R.M. (1990). Label-retaining cells reside in the bulge area of pilosebaceous unit: implications for follicular stem cells, hair cycle, and skin carcinogenesis. Cell *61*, 1329–1337.

Dekoninck, S., and Blanpain, C. (2019). Stem cell dynamics, migration and plasticity during wound healing. Nat. Cell Biol. *21*, 40–43.

Donati, G., Rognoni, E., Hiratsuka, T., Liakath-Ali, K., Hoste, E., Kar, G., Kayikci, M., Russell, R., Kretzschmar, K., Mulder, K.W., et al. (2017). Wounding induces dedifferentiation of epidermal Gata6+ cells and acquisition of stem cell properties. Nat. Cell Biol. *19*, 603–613.

Fafilek, B., Krausova, M., Vojtechova, M., Pospichalova, V., Tumova, L., Sloncova, E., Huranova, M., Stancikova, J., Hlavata, A., Svec, J., et al. (2013). Troy, a tumor necrosis factor receptor family

member, interacts with Lgr5 to inhibit Wnt signaling in intestinal stem cells. Gastroenterology *144*, 381–391.

Fuchs, E., and Weber, K. (1994). Intermediate FILAMENTS: structure, dynamics, function and disease. Annu. Rev. Biochem. *63*, 345–382.

Füllgrabe, A., Joost, S., Are, A., Jacob, T., Sivan, U., Haegebarth, A., Linnarsson, S., Simons, B.D., Clevers, H., Toftgard, R., et al. (2015). Dynamics of Lgr6+ progenitor cells in the hair follicle, sebaceous gland, and interfollicular epidermis. Stem Cell Reports *5*, 843–855.

Hashimoto, T., Schlessinger, D., and Cui, C. (2008). Troy binding to lymphotoxin-α activates NFκB mediated transcription. Cell Cycle *7*, 106–111.

Hashimshony, T., Senderovich, N., Avital, G., Klochendler, A., de Leeuw, Y., Anavy, L., Gennert, D., Li, S., Livak, K.J., Rozenblatt-rosen, O., et al. (2016). CEL-Seq2: sensitive highly-multiplexed single-cell RNA-seq. Genome Biol. *17*, 1–7.

Huch, M., Dorrell, C., Boj, S.F., van Es, J.H., Li, V.S., van de Wetering, M., Sato, T., Hamer, K., Sasaki, N., Finegold, M.J., et al. (2013). In vitro expansion of single Lgr5+ liver stem cells induced by Wnt-driven regeneration. Nature *494*, 247–250.

Hutchins, J.R.A., Toyoda, Y., Hegemann, B., Poser, I., Hériché, J.-K., Sykora, M.M., Augsburg, M., Hudecz, O., Buschhorn, B.A., Bulkescher, J., et al. (2010). Systematic analysis of human protein complexes identifies chromosome segregation proteins. Science *328*, 593–599.

Jaks, V., Barker, N., Kasper, M., van Es, J.H., Snippert, H.J., Clevers, H., and Toftgård, R. (2008). Lgr5 marks cycling, yet long-lived, hair follicle stem cells. Nat. Genet. *40*, 1291–1299.

Jensen, K.B., Collins, C.a., Nascimento, E., Tan, D.W., Frye, M., Itami, S., and Watt, F.M. (2009). Lrig1 expression defines a distinct multipotent stem cell population in mammalian epidermis. Cell Stem Cell *4*, 427–439.

Jensen, K.B., Driskell, R.R., and Watt, F.M. (2010). Assaying proliferation and differentiation capacity of stem cells using disaggregated adult mouse epidermis. Nat. Protoc. *5*, 898–911.

Jones, P.H., and Watt, F.M. (1993). Separation of human epidermal stem cells from transit amplifying cells on the basis of differences in integrin function and expression. Cell *73*, 713–724.

Jones, P.H., Harper, S., and Watt, F.M. (1995). Stem cell patterning and fate in human epidermis. Cell *80*, 83–93.

Joost, S., Zeisel, A., Jacob, T., Sun, X., La Manno, G., Lonnerberg, P., Linnarsson, S., and Kasper, M. (2016). Single-cell transcriptomics reveals that differentiation and spatial signatures shape epidermal and hair follicle heterogeneity. Cell Syst *3*, 221–237.e9.

Kadaja, M., Keyes, B.E., Lin, M., Amalia Pasolli, H., Genander, M., Polak, L., Stokes, N., Zheng, D., and Fuchs, E. (2014). SOX9: a stem cell transcriptional regulator of secreted niche signaling factors. Genes Dev. *28*, 328–341.

Kojima, T., Morikawa, Y., Copeland, N.G., Gilbert, D.J., Jenkins, N.A., Senba, E., and Kitamura, T. (2000). TROY, a newly identified member of the tumor necrosis factor receptor superfamily, exhibits a homology with Edar and is expressed in embryonic skin and hair follicles. J. Biol. Chem. *275*, 20742–20747.

Kretzschmar, K., and Clevers, H. (2017). Wnt/β-catenin signaling in adult mammalian epithelial stem cells. Dev. Biol. *428*, 273–282.

Kretzschmar, K., and Watt, F.M. (2012). Lineage tracing. Cell *148*, 33–45.

Kretzschmar, K., and Watt, F.M. (2014). Markers of epidermal stem cell subpopulations in adult mammalian skin. Cold Spring Harb Perspect. Med. *4*, 1–14.

Kretzschmar, K., Cottle, D.L., Donati, G., Chiang, M.-F., Quist, S.R., Gollnick, H.P., Natsuga, K., Lin, K.-I., and Watt, F.M. (2014). BLIMP1 is required for postnatal epidermal homeostasis but does not define a sebaceous gland progenitor under steady-state conditions. Stem Cell Reports *3*, 620–633.

Kretzschmar, K., Weber, C., Driskell, R.R., Calonje, E., and Watt, F.M. (2016). Compartmentalized epidermal activation of β-catenin differentially affects lineage reprogramming and underlies tumor heterogeneity. Cell Rep *14*, 269–281.

Kretzschmar, K., Post, Y., Bannier-Hélaouët, M., Mattiotti, A., Drost, J., Basak, O., Li, V.S.W., van den Born, M., Gunst, Q.D., Versteeg, D., et al. (2018). Profiling proliferative cells and their progeny in damaged murine hearts. Proc. Natl. Acad. Sci. *115*, E12245–E12254.

Lapouge, G., Youssef, K.K., Vokaer, B., Achouri, Y., Michaux, C., Sotiropoulou, P.a., and Blanpain, C. (2011). Identifying the cellular origin of squamous skin tumors. Proc. Natl. Acad. Sci. U. S. A. *108*, 7431–7436.

Lim, X., Tan, S.H., Koh, W.L.C., Chau, R.M.W., Yan, K.S., Kuo, C.J., van Amerongen, R., Klein, A.M., and Nusse, R. (2013). Interfollicular epidermal stem cells self-renew via autocrine Wnt signaling. Science *342*, 1226–1230.

Lim, X., Tan, S.H., Yu, K., Lou, Lim, S.B.H., and Nusse, R. (2016). *Axin2* marks quiescent hair follicle bulge stem cells that are maintained by autocrine Wnt/β-catenin signaling. Proc. Natl. Acad. Sci. *113*, E1498–E1505.

Love, M.I., Huber, W., and Anders, S. (2014). Moderated estimation of fold change and dispersion for RNA-seq data with DESeq2. Genome Biol. *15*, 550.

Madisen, L., Zwingman, T.A., Sunkin, S.M., Oh, S.W., Zariwala, H.A., Gu, H., Ng, L.L., Palmiter, R.D., Hawrylycz, M.J., Jones, A.R., et al. (2010). A robust and high-throughput Cre reporting and characterization system for the whole mouse brain. Nat. Neurosci. *13*, 133–140.

Mascré, G., Dekoninck, S., Drogat, B., Youssef, K.K., Brohée, S., Sotiropoulou, P.A., Simons, B.D., and Blanpain, C. (2012). Distinct contribution of stem and progenitor cells to epidermal maintenance. Nature *489*, 257–262.

Morris, R.J., Bortner, C.D., Cotsarelis, G., Reece, J.M., Trempus, C.S., Faircloth, R.S., and Tennant, R.W. (2003). Enrichment for living murine keratinocytes from the hair follicle bulge with the cell surface marker CD34. J. Invest. Dermatol. *120*, 501–511.

Müller-Röver, S., Handjiski, B., van der Veen, C., Eichmu, S., Foitzik, K., Mckay, I.A., Stenn, K.S., and Paus, R. (2001). A comprehensive guide for the accurate classification of murine hair follicles in distinct hair cycle stages. J. Invest Dermatol. *117*, 3–15.

Muraro, M.J., Dharmadhikari, G., Grün, D., Groen, N., Dielen, T., Jansen, E., van Gurp, L., Engelse, M.A., Carlotti, F., de Koning, E.J.P., et al. (2016). A single-cell transcriptome atlas of the human pancreas. Cell Syst *3*, 385–394.e3.

Nijhof, J.G.W., Braun, K.M., Giangreco, A., van Pelt, C., Kawamoto, H., Boyd, R.L., Willemze, R., Mullenders, L.H.F., Watt, F.M., de Gruijl, F.R., et al. (2006). The cell-surface marker MTS24 identifies a novel population of follicular keratinocytes with characteristics of progenitor cells. Development *133*, 3027–3037.

Nowak, J., Polak, L., Pasolli, H.A., and Fuchs, E. (2008). Hair follicle stem cells are specified and function in early skin morphogenesis. Cell Stem Cell *3*, 33–43.

Page, M.E., Lombard, P., Ng, F., Göttgens, B., and Jensen, K.B. (2013). The epidermis comprises autonomous compartments maintained by distinct stem cell populations. Cell Stem Cell *13*, 471–482.

Pispa, J., Pummila, M., Barker, P.A., Thesleff, I., and Mikkola, M.L. (2008). Edar and Troy signalling pathways act redundantly to regulate initiation of hair follicle development. Hum. Mol. Genet. *17*, 3380–3391.

Raymond, K., Richter, A., Kreft, M., Frijns, E., Janssen, H., Slijper, M., Praetzel-Wunder, S., Langbein, L., and Sonnenberg, A. (2010). Expression of the orphan protein Plet-1 during trichilemmal differentiation of anagen hair follicles. J. Invest. Dermatol. *130*, 1500–1513.

Rheinwald, J.G., and Green, H. (1975). Serial cultivation of strains of human epidermal keratinocytes: the formation of keratizing colonies from single cells. Cell *6*, 331–344.

Snippert, H.J., Haegebarth, A., Kasper, M., Jaks, V., van Es, J.H., Barker, N., van de Wetering, M., van den Born, M., Begthel, H., Vries, R.R.G., et al. (2010). Lgr6 marks stem cells in the hair follicle that generate all cell lineages of the skin. Science *327*, 1385–1390.

Stange, D.E., Koo, B., Huch, M., Sibbel, G., Basak, O., Lyubimova, A., Kujala, P., Bartfeld, S., Koster, J., Geahlen, J.H., et al. (2013). Differentiated Troy+ chief cells act as reserve stem cells to generate all lineages of the stomach epithelium. Cell *155*, 357–368.

Tan, D.W.M., Jensen, K.B., Trotter, M.W.B., Connelly, J.T., Broad, S., and Watt, F.M. (2013). Single-cell gene expression profiling reveals functional heterogeneity of undifferentiated human epidermal cells. Development *140*, 1433–1444.

Uhlén, M., Fagerberg, L., Hallström, B.M., Lindskog, C., Oksvold, P., Mardinoglu, A., Sivertsson, Å., Kampf, C., Sjöstedt, E., Asplund, A., et al. (2015). Tissue-based map of the human proteome. Science *347*, 1260419.

Wang, F., Flanagan, J., Su, N., Wang, L., Bui, S., Nielson, A., Wu, X., Vo, H.-T., Ma, X.-J., and Luo, Y. (2012). RNAscope: a novel in situ RNA analysis platform for formalin-fixed, paraffin-embedded tissues. J. Mol. Diagn. *14*, 22–29.

Stem Cell Reports, Volume *16*

Supplemental Information

Troy/Tnfrsf19 marks epidermal cells that govern interfollicular epidermal renewal and cornification

Kai Kretzschmar, Kim E. Boonekamp, Margit Bleijs, Priyanca Asra, Mandy Koomen, Susana M. Chuva de Sousa Lopes, Barbara Giovannone, and Hans Clevers

Supplemental Information

Supplemental Figures

Figure S1, related to Figure 1

Figure S2, related to Figure 1

Figure S3, related to Figure 2

Figure S4, related to Figure 4

Figure S5, related to Figure 5

Figure S6, related to Figure 6

Supplemental Tables

Table S1, related to Figure 2

Supplemental Experimental Procedures

Supplemental References

Supplemental Figures

Figure S1

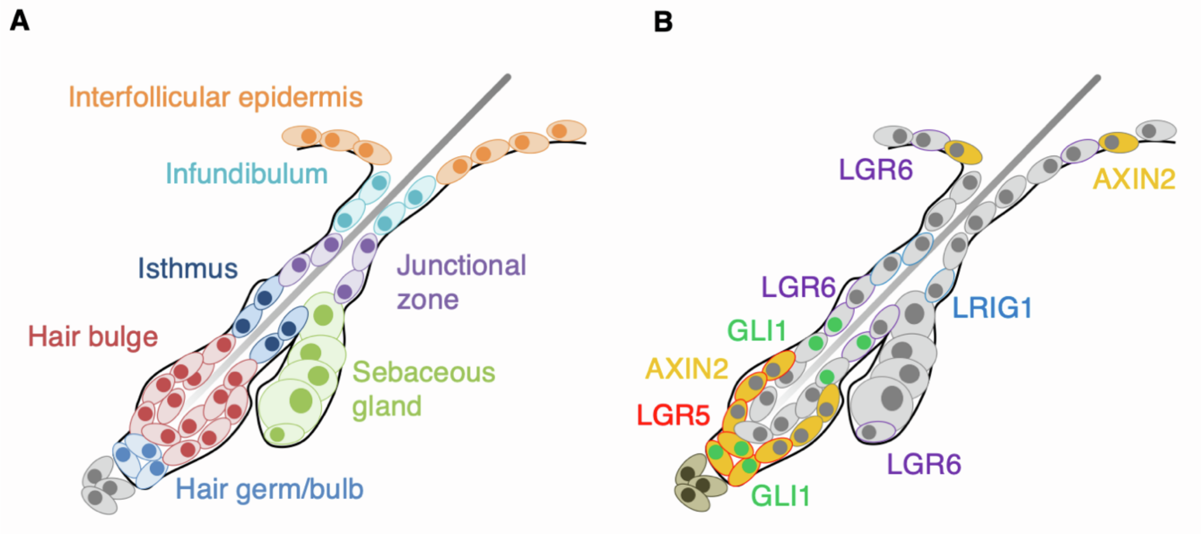

Figure S1. Epidermal compartments (A) and stem cell markers (B), related to Figure 1.

Figure S2

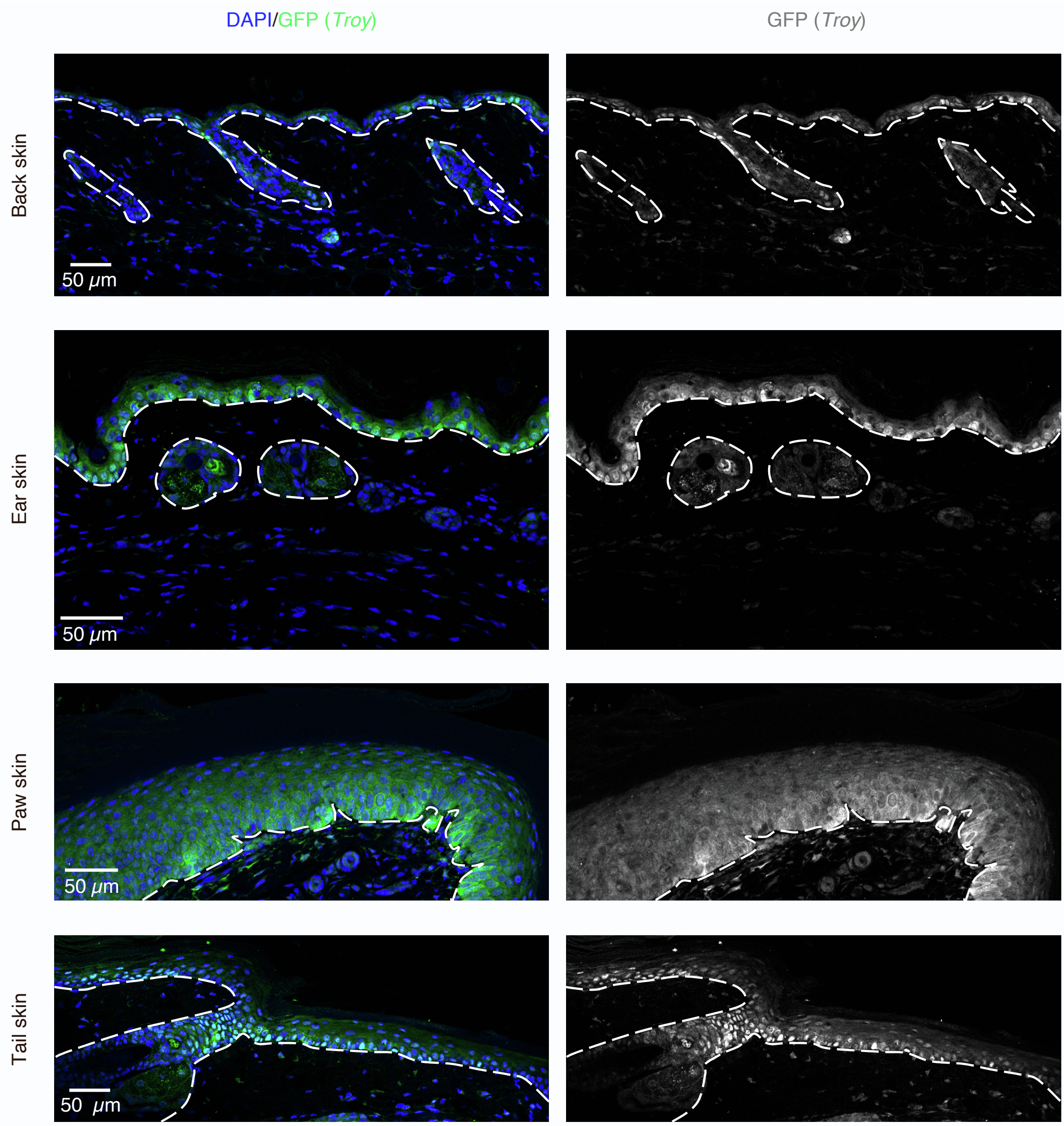

Figure S2. *Troy*-EGFP+ expression in adult murine skin, related to Figure 1.

Paraffin resections of mouse back, ear, paw and tail skin of adult (P50) *Troy*-EGFP+ mice. Sections were stained for EGFP (green/grey) and nuclei were counterstained with DAPI (blue).

Figure S3

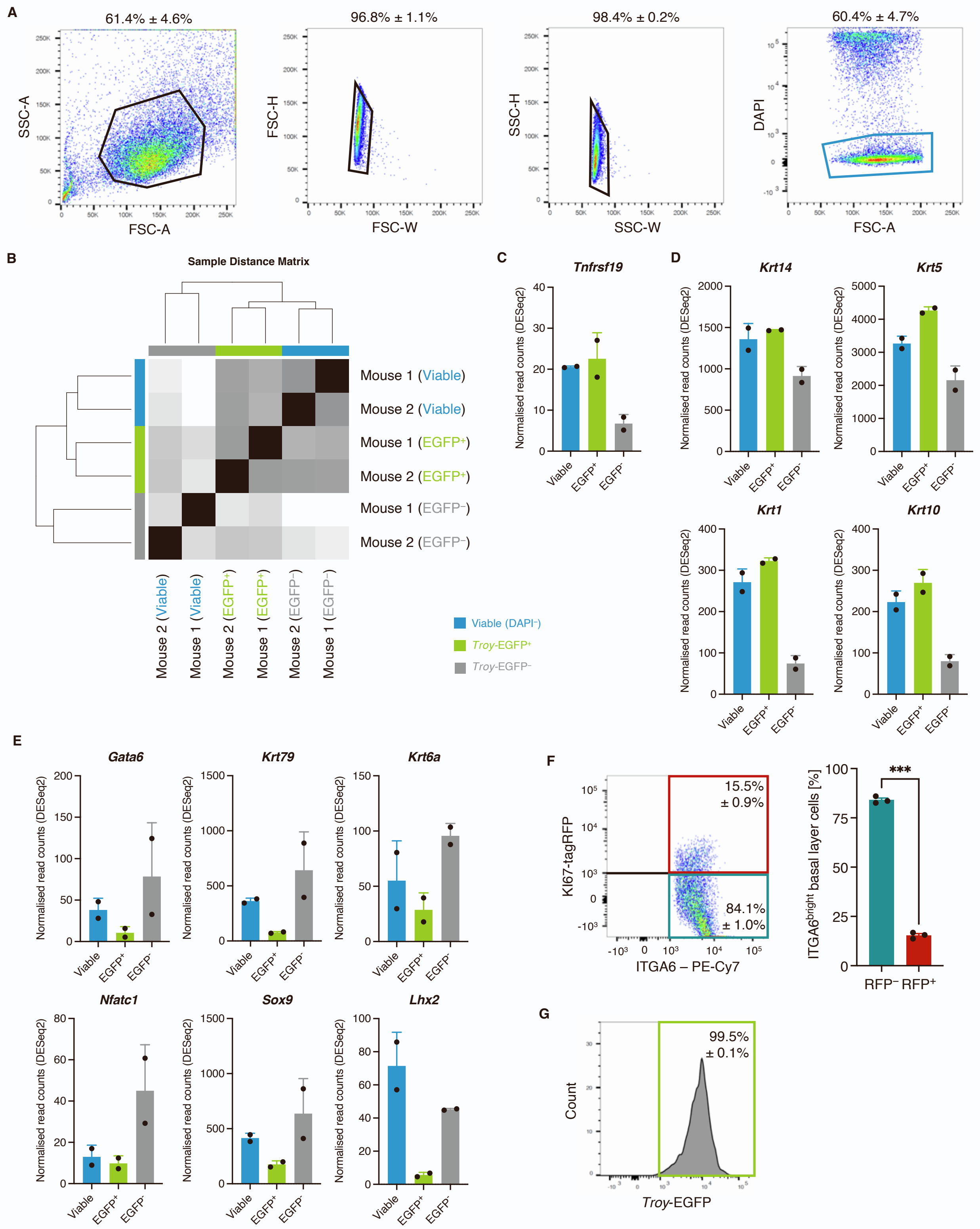

Figure S3. Sorting strategy and bulk mRNA-sequencing, related to Figure 2.

(A) Gating strategy.

(B) Sample distance matrix for the sorted populations used for bulk mRNA sequencing

(C-E) Column charts indicating normalised read counts (DESeq2) of *Troy* **(D),** IFE lineage markers (C) and HF lineage markers (E).

(F) Representative flow cytometry scatter plot of viable cells assessed for expression of ITGA6 and Ki67-tagRFP. Data in the gates indicate the percentage of cells per gate as mean average ± S.E.M. (*n* = 3 mice). Column chart indicating the percentage of Ki67-tagRFP$^{–}$ and Ki67-tagRFP^{+} cells within the ITGA6^{bright} population. The data are presented as mean average ± S.E.M. (*n* = 3 mice). Dots represent the individual data points derived from each mouse.

(G) Histogram indicating the percentage of *Troy*-EGFP^{+} within the ITGA6^{high} Ki67-tagRFP^{+} cell population. The data are presented as mean ± S.E.M. (*n* = 3 mice).

Figure S4

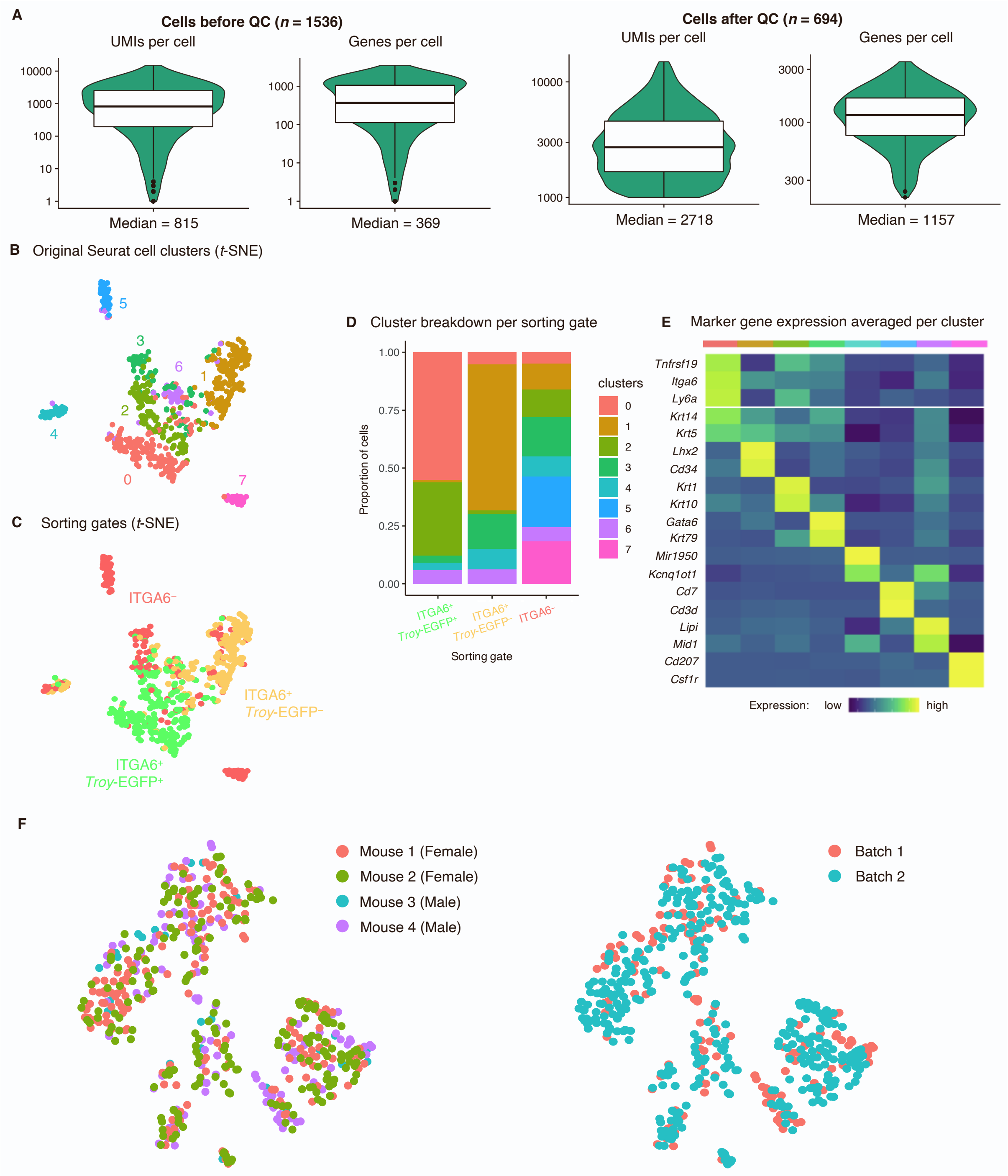

Figure S4. Quality metrics and initial clustering for scRNA-sequencing experiments using *Troy*$^{EGFP\text{-}IRES\text{-}CreERT2}$ mice, related to Figure 4.

(A) UMIs and genes per cell before and after quality control (QC).

(B) t-SNE plot indicating the 7 different clusters identified in the original clustering (clusters 0–7).

(C) *t*-SNE plot projecting the sorting gates on the original *t*-SNE map.

(D) Stacked column chart indicating cluster breakdown by gate.

(E) Heatmap implicating key marker gene expression averaged per cell population for all identified original clusters.

(F) *t*-SNE plot projecting different mice (left) and different sequencing batches (right).

Figure S5

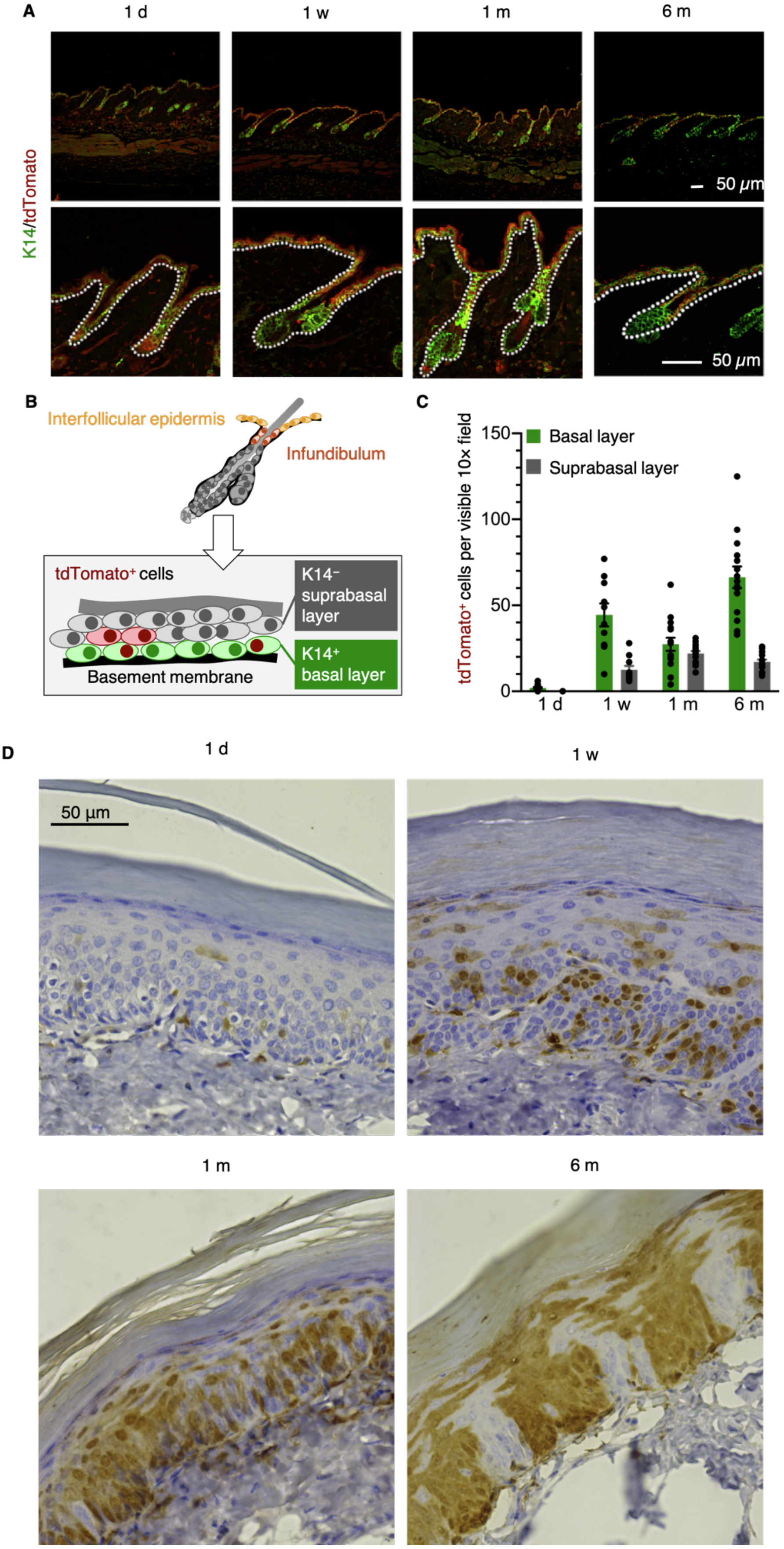
A
1 d
1 w
1 m
6 m
K14/tdTomato
50 μm
50 μm
B
Interfollicular epidermis
Infundibulum
tdTomato+ cells
K14− suprabasal layer
K14+ basal layer
Basement membrane
C
Basal layer
Suprabasal layer
tdTomato+ cells per visible 10× field
150
100
50
0
1 d
1 w
1 m
6 m
D
1 d
1 w
50 µm
1 m
6 m

Figure S5. Quantification of tdTomato labelling in the IFE basal and suprabasal layers, related to Figure 5.

(A) Paraffin slides of murine back skin of *Troy*$^{EGFP\text{-}IRES\text{-}CreERT2}$ x *Rosa26-LSL-tdTomato* mice induced with tamoxifen for indicated times stained against tdTomato (RFP; red) and keratin 14 (K14; green).

(B) Schematic overview of K14 expression in the IFE.

(C) Quantification of tdTomato cells in the basal layer (K14^{-}) or suprabasal layer (K14^{+})

(D) Paraffin slides of murine paw skin of *Troy*$^{EGFP\text{-}IRES\text{-}CreERT2}$ x *Rosa26-LSL-tdTomato* mice induced with tamoxifen for indicated times stained against tdTomato (RFP; brown) counter stained for nuclei with haematoxylin.

Figure S6

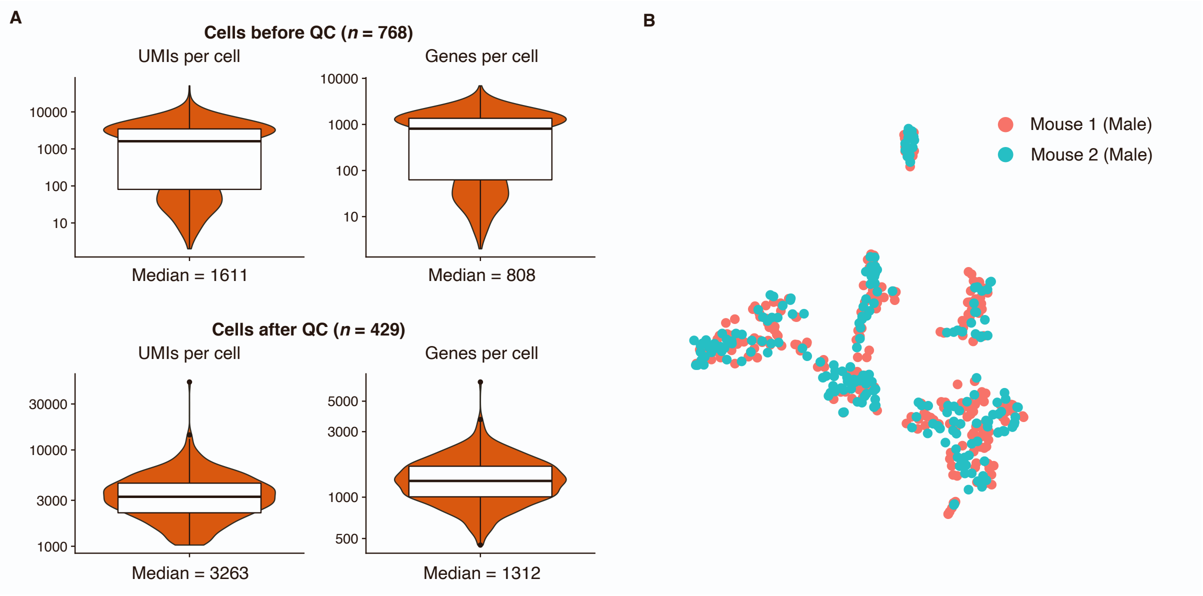

Figure S6. Quality metrics for scRNA-seq experiments using *Troy*$^{\text{EGFP-IRES-CreERT2}}$ x *Rosa26*$^{\text{LSL-tdTomato}}$ mice, related to Figure 6.

(A) UMIs and genes per cell before and after quality control (QC).

(B) *t*-SNE plot projecting different mice used in this experiment.

Supplemental Tables

Table S1. List of differentially expressed genes between *Troy*-EGFP^{+} and *Troy*-EGFP$^{–}$ ITGA6^{bright} epidermal basal layer cells, related to Figure 2.

Supplemental Experimental Procedures

Animal Experiments

In order to characterise *Troy* expression during epidermal development and HF cycling the following embryonic (E) and postnatal (P) days were collected: E12.5, E15.5, E17.5, P1, P2, P25, P42 and P49. For flow sorting experiments, both male and female mice of the age between 7 and 9 weeks were used.

For genetic lineage tracing experiments *Troy*$^{EGFP\text{-}IRES\text{-}CreERT2}$ mice were crossed to *Rosa26*$^{LSL\text{-}tdTomato}$ mice to label all progeny. All lineage tracing experiments were induced by a single injection of tamoxifen (Sigma) at 7–9 of weeks of age when the HFs were in telogen (resting phase). Tamoxifen was dissolved in sunflower oil (Sigma) and administered by IP (5 mg tamoxifen per mouse). Tissue was collected at 1 day, 7 days, 1 month and 6 months post tamoxifen injection.

Cell Lines

293T-HA-RspoI-Fc cells provided by Calvin Kuo, Standford University, Stanford, CA were grown at 5% CO_2 to generate R-spondin 1-conditioned medium (Drost et al., 2016). 293T-Noggin-Fc cells were grown at 5% CO_2 to generate Noggin-conditioned medium (Cattaneo et al., 2020; Heijmans et al., 2013).

Murine Epidermal Keratinocyte Isolation

Mice were sacrificed and their back skin was shaved. Back skin was isolated and washed in 70% EtOH twice and once in PBS without Ca^{2+} and Mg^{2+} (PBSO). Subsequently, the subcutaneous fat was removed from the dermal side by scraping with a scalpel. Post-cleaning, the tissue was incubated with the epidermal side up in freshly prepared 0.025% trypsin in Advanced Dulbecco's Modified Eagle Medium (DMEM)/F12 overnight at 4 ˚C. The following day, the tissue was transferred to a clean dish and the cells were scraped from the epidermal side using a scalpel. After mincing the tissue using scalpels, the cells were resuspended in 20 ml DMEM and pipetted vigorously up and down for 20 times in a 50 ml conical tube. After that cells were filtered through a 70-μm cell strainer and separated over 2 individual 15 ml conical tubes and centrifuged at 300×*g* for 5 minutes at 4 °C. Subsequently, cells were washed twice in DMEM.

Organoid Experiments

Flow cytometry purified cells were seeded in BME in a density of 2500 cells per 10 µl BME in a 48-well plate in triplicate. Organoids were grown for 7 days at 37 °C in epidermal expansion medium (EEM): Advanced DMEM/F12 supplemented with penicillin/streptomycin (100 U/l), Hepes, (10 mM), GlutaMAX (1×), B27 supplement (1×), N-Acetylcysteine-1 (1 mM), Noggin-conditioned medium (5%), R-spondin 1-conditioned medium (5%), acidic FGF1 (100 ng/ml), Heparin (0.0001%), Forskolin (10 ng/ml), Rho kinase inhibitor (Y-27632; 10 µM), and Primocin (1×). Subsequently, organoid cultures were imaged using a brightfield microscope (n = 3). Quantification of organoid number and size was performed in ImageJ using the "analyse particles" tool, all colonies and organoids were manually outlined and colony/organoid size and number was noted. Thereafter, imaged organoids were lysed with CellTiter-Glo® 3D Cell Viability Assay reagent (Promega, 1:1 diluted with Advanced DMEM/F12 +++) for 20 minutes at room temperature while shaking. Luminescence was detected using a Berthold reader.

RNA Sequencing

mRNA sequencing samples were processed according to the CEL-Seq2 method (Hashimshony et al., 2016). For bulk mRNA-sequencing, after storage at –80 °C, RNA was extracted from the bulk sorted samples by standard TRIzol extraction protocols followed by RNA precipitation with 2 μg GlycoBlue (Ambion) overnight at –80 °C as described elsewhere (Kretzschmar et al., 2018). Post overnight incubation, bulk RNA samples were labelled with UMI barcode primers in a reverse transcription reaction mix (Invitrogen). Subsequently, samples were pooled into specific libraries and processed for submission. For single-cell mRNA-sequencing following the SORT-seq method (Muraro et al., 2016),

cells were directly sorted in lysis buffer containing specific primers. After storage in –80 °C RNA the reactions were subjected to first strand and second strand synthesis. Subsequently, samples were pooled into plate-specific libraries and further processed for submission (Muraro et al., 2016). Amplified RNA for bulk sequencing and single-cell sequencing libraries were used to generate complementary (cDNA) libraries using Illumina TruSeq primers. All submitted libraries were sequenced on an Illumina NextSeq500 using 75bp pair-end sequencing with high output (150 million reads per run).

Histology

Fixed tissue was subjected to subsequent dehydration steps and embedded in paraffin blocks. Paraffin blocks were sectioned to generate sections of 4 μm. Sections were re-hydrated following conventional protocols and subjected to immunohistochemistry staining against either Keratin 14 (clone LL002, Thermo Fisher Scientific), EGFP (ab13970, Abcam) or tdTomato (600-401-379, Rockland). In short, antigen retrieval was performed by boiling slides in citrate buffer pH 6. Sections were stained with primary antibody overnight at 4 °C and incubated with secondary antibody for 2 hours at room temperature. For immunohistochemistry, secondary antibodies conjugated to HRP were used followed by development using 3,3'-diaminobenzidine (DAB). Slides were counterstained with haematoxylin, dehydrated and mounted using Pertex. For immunofluorescence, secondary antibodies conjugated to a fluorophore were used, followed by counterstaining using DAPI and mounting using Vectashield (Vectorlabs).

For tail whole mount stainings, tails were processed according to the protocol described by Braun and colleagues (2003). In short, tail epidermis was separated from the dermis following 4-hour incubation in 5 mM EDTA at 37 °C. Tail whole mounts were fixed in 4% paraformaldehyde for 15 minutes at room temperature and stored at 4 °C in PBSO + 0.02 % Azide. For tail whole mount staining, tails were first incubated in blocking buffer (1x TBS, 0.5 % (v/v) TritonX-100, 0.25% (v/v) fish skin gelatine, 0.5% (w/v) milk powder) for an hour at room temperature. All steps were performed protected from light. Primary antibody was incubated overnight at room temperature followed by washes in PBSO + 0.02 % Tween (PBS-T) the next day. Primary antibodies were: EGFP (ab13970, Abcam), mouse anti-Human Ki67 (Clone B56, 550609, BD Pharmingen), rat anti-human/mouse CD49f-PE (clone GoH3, 555736, BD Biosciences) and anti-tdTomato (600-401-379, Rockland). Secondary antibody (Thermo Scientific) was incubated overnight at room temperature followed by washes in PBSO the next day. Tail whole mounts were counterstained with DAPI for 15 minutes at room temperature followed by a wash with MilliQ water. Tail whole mounts were mounted on slides using ProLong gold (Invitrogen). Stained sections were stored at 4 °C in the dark until imaging.

In order to look at expression of human *TROY* we conducted RNAscope assays (Wang et al., 2012). RNA scope analysis was performed on paraffin-embedded human foetal and adult skin. Sectioning was performed maximum 1 week ahead of staining and slides where dried overnight at room temperature. RNAscope was performed according to the manufacture outlines. All paraffin sections of human skin where subjected to a 15-minute boiling step in target retrieval buffer and a 30-minute incubation at 40 °C with RNAscope protease plus reagent. Pre-treated slides were incubated for 2 hours at 40 °C with RNAscope Probe Hs TNFRSF19 (Channel 1) (441931, ACD Biotechne). Post washing, amplification trees were built according to manufactures guidelines and the *TROY*-specific signal was developed and visualized with Opal 570 fluorophores (FP14880001KT, Akoya). Slides were either co-stained with DAPI and mounted in ProLong Gold directly or subjected to a complementary immunostaining against keratin 14 (1:100; Thermo Scientific, LL002) according to manufactures guidelines followed by DAPI counter staining and mounting in ProLong Gold. Paraffin sections of P50 murine back skin were processed similarly to the human sections and were stained with RNAscope Probes Ms Tnfrs19 (Channel 1 – Opal 570) (420241 ACD Biotechne) and Ms Keratin 10 (Channel 2 – Opal 690) (457901-C2, ACD Biotechne).

Bioinformatics Analysis

Bulk mRNA sequencing samples were analysed using the DESeq2 package (Love et al., 2014) with standard parameters in R/Bioconductor environment. Raw read counts were normalised using the DESeq2 median of ratios method and these normalised read counts were used to generate the column charts depicted in Figure S3C-E. Differentially expressed (DE) genes (see Figure 2D and Table S1) were identified from the normalised read count table using DESeq2 with a cut-off of adjusted $p < 0.05$.

The sample distance matrix was generated after `rlog` transformation. Heatmaps in Figures 2D and S3B were generated using gplots' `heatmap.2` function.

Single-cell mRNA sequencing libraries were analysed using the Seurat v3 package (Butler et al., 2018) in R/Bioconductor environment. ERCC92 spike-ins and mitochondrial genes and cell transcriptomes with less than 1,000 transcript UMIs were removed from the dataset (Figures S4 and S6). The remaining transcriptomes were normalised using Seurat's `SCTransform` function using the `vars.to.regress` setting to remove confounding sources of variation such as total UMI counts, total gene counts and sequencing run. For initial cell type analysis, cell clusters were generated based on gene expression similarities using Seurat's `FindClusters` at a `resolution = 0.8` (Figure S4B). Marker gene expression per cluster was averaged using Seurat's `AverageExpression` function and plotted using Seurat's `DoHeatmap` function to generate Figures 4H and S4E. For the further analysis cell cluster enriched for necrotic cell markers such as *Mir1950* and *Kcqn1ot1* (Kretzschmar et al., 2018) or enriched for non-epidermal cells marked by *Cd7*, *Cd3d*, *Lipi*, *Mid1*, *Cd207* and/or *Csf1r* were removed (Figure 4). Cell cycle analysis (Figure 4F) was performed using Seurat's `CellCycleScoring` function. The transcriptome dataset collected after lineage tracing using *Troy*$^{EGFP\text{-}IRES\text{-}CreERT2}$ × *Rosa26*-LSL-tdTomato mice (Figure 6) was projected onto the initial *Troy*$^{EGFP\text{-}IRES\text{-}CreERT2}$ transcriptome dataset using Seurat's `FindTransferAnchors` function after quality control, filtering and removal of necrotic and non-epidermal cells.

Statistics

Statistically significant differences were determined using two-tailed Student's tests unless stated otherwise. Statistically analyses were performed using GraphPad Prism version 9.1.0, except for the statistics performed during bioinformatics analyses.

Supplemental References

Braun, K.M., Niemann, C., Jensen, U.B., Sundberg, J.P., Silva-Vargas, V., and Watt, F.M. (2003). Manipulation of stem cell proliferation and lineage commitment: visualisation of label-retaining cells in wholemounts of mouse epidermis. Development *130*, 5241–5255.

Butler, A., Hoffman, P., Smibert, P., Papalexi, E., and Satija, R. (2018). Integrating single-cell transcriptomic data across different conditions, technologies, and species. Nat. Biotechnol. *36*, 411–420.

Cattaneo, C.M., Dijkstra, K.K., Fanchi, L.F., Kelderman, S., Kaing, S., van Rooij, N., van den Brink, S., Schumacher, T.N., and Voest, E.E. (2020). Tumor organoid–T-cell coculture systems. Nat. Protoc. *15*, 15–39.

Drost, J., Karthaus, W.R., Gao, D., Driehuis, E., Sawyers, C.L., Chen, Y., and Clevers, H. (2016). Organoid culture systems for prostate epithelial and cancer tissue. Nat. Protoc. *11*, 347–358.

Hashimshony, T., Senderovich, N., Avital, G., Klochendler, A., de Leeuw, Y., Anavy, L., Gennert, D., Li, S., Livak, K.J., Rozenblatt-rosen, O., et al. (2016). CEL-Seq2 : sensitive highly-multiplexed single-cell RNA-Seq. Genome Biol. *17*, 1–7.

Heijmans, J., van Lidth de Jeude, J.F., Koo, B.K., Rosekrans, S.L., Wielenga, M.C.B., van de Wetering, M., Ferrante, M., Lee, A.S., Onderwater, J.J.M., Paton, J.C., et al. (2013). ER stress causes rapid loss of intestinal epithelial stemness through activation of the unfolded protein response. Cell Rep. *3*, 1128–1139.

Kretzschmar, K., Post, Y., Bannier-Hélaouët, M., Mattiotti, A., Drost, J., Basak, O., Li, V.S.W., van den Born, M., Gunst, Q.D., Versteeg, D., et al. (2018). Profiling proliferative cells and their progeny in damaged murine hearts. Proc. Natl. Acad. Sci. *115*, E12245 LP-E12254.

Love, M.I., Huber, W., and Anders, S. (2014). Moderated estimation of fold change and dispersion for RNA-seq data with DESeq2. Genome Biol. *15*, 550.

Muraro, M.J., Dharmadhikari, G., Grün, D., Groen, N., Dielen, T., Jansen, E., van Gurp, L., Engelse, M.A., Carlotti, F., de Koning, E.J.P., et al. (2016). A single-cell transcriptome atlas of the human pancreas. Cell Syst. *3*, 385-394.e3.

Wang, F., Flanagan, J., Su, N., Wang, L., Bui, S., Nielson, A., Wu, X., Vo, H.-T., Ma, X.-J., and Luo, Y. (2012). RNAscope: a novel in situ RNA analysis platform for formalin-fixed, paraffin-embedded tissues. J. Mol. Diagnostics *14*, 22–29.
